# Small molecule-nanobody conjugate induced proximity controls intracellular processes and modulates endogenous unligandable targets

**DOI:** 10.1038/s41467-023-37237-x

**Published:** 2023-03-24

**Authors:** Xiaofeng Sun, Chengjian Zhou, Simin Xia, Xi Chen

**Affiliations:** 1 Laboratory of Chemical Biology & Frontier Biotechnologies, The HIT Center for Life Sciences (HCLS), Harbin Institute of Technology (HIT), Harbin, 150001 PR China; 2 School of Life Science and Technology, HIT, Harbin, 150001 PR China

**Keywords:** Chemical tools, Proteins, Chemical synthesis, Nanobiotechnology, Molecular engineering

## Abstract

Chemically induced proximity (CIP) is a powerful tool to study cellular functions. However with current CIP inducers it is difficult to directly modulate unligandable and endogenous targets, and therapeutic translational potential is also restricted. Herein, we combine CIP and chemical nanobody engineering and create cell-permeable small molecule-nanobody conjugate inducers of proximity (SNACIPs). The SNACIP inducer cRGT carrying a cyclic cell-penetrating peptide rapidly enters live cells and dimerizes eDHFR and GFP-variants. cRGT enables minute-scale, reversible, no-wash and dose-dependent control of cellular processes including signaling cascade, cargo transport and ferroptosis. Small-molecule motifs can also be installed via post-translational modifications. Therefore, latent-type SNACIPs including cRTC are designed that are functionally assembled inside living cells. cRTC contains a nanobody against an intrinsically disordered protein TPX2, a microtubule nucleation factor overexpressed in various cancers. Cancer cell proliferation is inhibited and tumor growth is suppressed in vivo. Hence, SNACIPs are valuable proximity inducers for regulating cellular functions.

## Abstract (web summary)

Induced proximity can be used to control diverse cellular processes. Here, the authors develop nanobody-based proximity inducers called SNACIPs, which can be used to regulate either tagged or endogenous proteins, and demonstrate their use in blocking microtubule nucleation for tumour growth inhibition in vivo.

## Introduction

Proximity-inducing mechanism orchestrates the proceeding of diverse cellular processes. Chemically induced proximity or dimerization (CIP or CID)^1–6^ that uses cell-permeable bivalent small molecules to bring two proteins in close proximity is a powerful tool in regulating many biological processes, such as signal transduction^4^, selective autophagy^7^, conditional protein degradation^8^, axonal transport^9^, cell therapeutic applications^10^, and many others^11–15^. However, CIP typically requires genetic fusion of an additional binding tags to a protein of interest (POI). Therefore, CIPs are difficult to directly modulate unligandable and endogenous targets, suffer from background activities from endogenous POIs, and the potential in therapeutic translation is restricted due to the requirement of genetic modification.

**Fig. 1:**
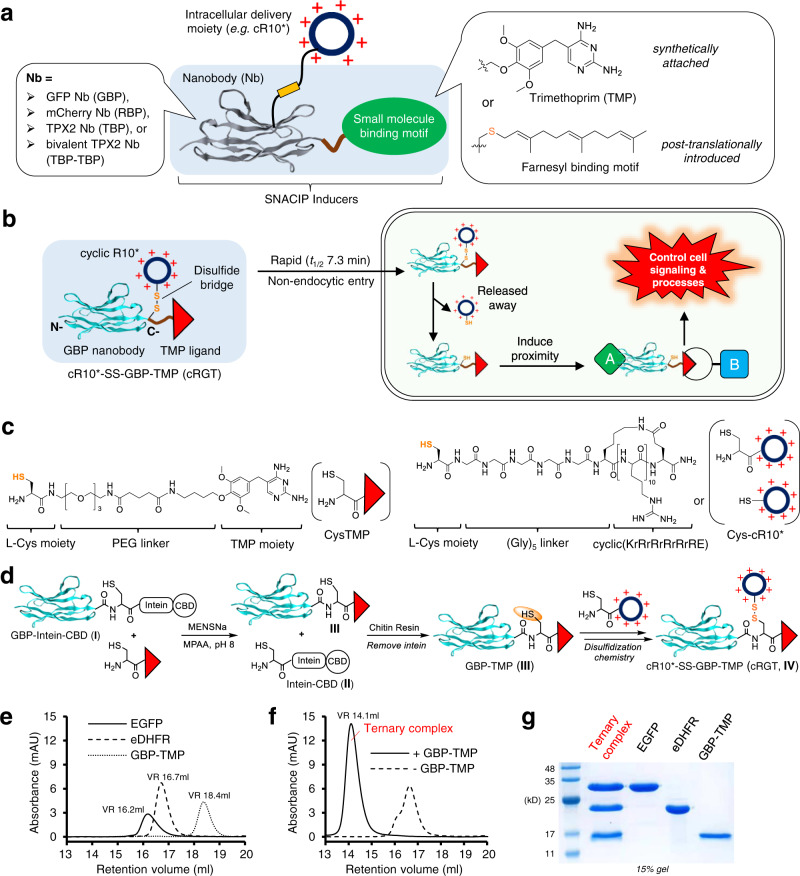
Design and two-step assembly of the cRGT inducer via C-terminal nanobody functionalization and related biochemical characterizations. **a** General structural elements of SNACIP inducers in which the small molecule binding motif can be introduced either synthetically or posttranslationally. **b** Schematic view of the structure and working mechanisms of cRGT. **c** Structural elements of CysTMP (left) and Cys-cR10* (r: D-Arg, R: L-Arg) (right). **d** Two-step assembly of cRGT (**IV**) via EPL followed by disulfidization chemistry. **e** Representative size-exclusion chromatographic (SEC) analysis of 1 nmol EGFP, eDHFR or GBP-TMP (**III**) using *Superdex 200 Increase 10/300 GL* column at a flow rate of 0.4 ml·min^-1^ revealed retention volumes (VR) of 16.2 ml, 16.7 ml and 18.4 ml, respectively. **f** SEC analysis of 1 nmol of EGFP/GBP-TMP/eDHFR ternary complex (solid line) which revealed a single peak at VR 14.1 ml while SEC analysis of a mixture of 1 nmol EGFP and 1 nmol eDHFR in the absence of GBP-TMP (dashed line) revealed no complex formation; same SEC instrumentation and parameters were applied as in **e**. **g** Denaturing SDS-PAGE analysis of EGFP, eDHFR and GBP-TMP used in **e** as well as the EGFP/GBP-TMP/eDHFR ternary complex corresponding to the VR 14.1 ml peak in **f**.

A nanobody is a camelid-derived single-chain V_HH_ antibody fragment with a substantially reduced size (~15 kD) than traditional antibodies (~150 kD)^16^. Compared to small molecules, nanobodies can be generated with high specificities and nanomolar, or even picomolar level^17^ high affinities towards their binding partners. Therefore, the possibility to use nanobody as a proximity-inducing module could significantly expand the application potential of CIPs and broaden the utility of nanobodies to modulate intracellular targets. Herein, we introduced the concept of small molecule-nanobody conjugate induced proximity (SNACIP) that utilizes a nanobody as one warhead to target a specific POI and a small molecule binding motif to induce proximity (Fig. 1a).

A small molecule-nanobody conjugate is usually not cell-permeable and cannot be directly employed to regulate cellular processes inside living cells. Traditional intracellular delivery modules such as linear cell-penetrating peptides (CPPs)^18^ and other more elegant biocompatible cargos such as engineered C3 toxin^19^ mediate intracellular delivery via internalization. This process is typically slow and suffers from lysosomal degradation. Therefore, we also used an intracellular cleavable cyclic CPP, named cR10*, with a chirality opposite to previously known analogs^20,21^ and found it to be an ideal cyclic delivery module.

Microtubule (MT) nucleation in spindle assembly is essential for maintaining life^22^ and the dysregulation of which is associated with many diseases^23^. Though microtubule targeting agents (MTAs) that directly bind microtubules have been introduced for cancer chemotherapy, agents that directly modulate microtubule nucleation are rare. Microtubule nucleation involves the concerted action of large protein complexes and intrinsically disordered protein factors^22^, which makes it hard to develop related small molecule modulators via, for example, structure-guided drug design. In this regard, we introduced SNACIPs to target the intrinsically disordered microtubule nucleation factor TPX2 and demonstrated the value of SNACIP to modulate endogenous oncogenic unligandable targets for therapeutic intervention.

## Results

### Design, assembly, and biochemical characterizations of the general-purpose SNACIP inducer cRGT

Because green fluorescent protein (GFP) is among the most widely used fluorescent proteins (FPs), direct modulation of GFP-fused protein’s function will have widespread applications. Furthermore, as GFP is a fluorophore, simultaneous regulation and visualization can be achieved. However, to date, small molecule ligands that directly bind to GFP with high affinities have not been reported, rendering GFP an unligandable target. In this study, we first designed the general-purpose SNACIP inducer cR10*-SS-GBP-TMP, or cRGT (Fig. 1b). cRGT features a GFP binding protein (GBP, K_d_ = 1.4 nM)^24^ nanobody module as one warhead and a trimethoprim (TMP) ligand as the other warhead. The anti-biotic TMP has a high affinity (K_i_ = 1.3 nM) for *E. coli* dihydrofolate reductase (eDHFR, or ED), but a much lower affinity (K_i_ = 4–8 μM) for mammalian DHFR^25^. Hence, cRGT can potentially induce a strong heterodimerization between proteins of interest fused to GFP and eDHFR. Also, we designed a cyclic decaarginine peptide cR10* to decorate the GBP-TMP conjugate via a disulfide bridge, a known excellent intracellular cleavable linker. After cRGT smoothly crosses the plasma membrane (PM), cR10* is readily cleaved in the reducing intracellular environment.

Prior to the assembly of cRGT, CysTMP, which is used to attach a TMP ligand to GBP, was chemically synthesized. CysTMP features an N-terminal cysteine, a water-soluble PEG linker and a TMP moiety to bind an eDHFR tag (Fig. 1c, left). In parallel, Cys-cR10* that features a L-Cys residue, a (Gly)_5_ linker and a cyclic(KrRrRrRrRrRE) cell-penetrating peptide moiety (Fig. 1c, right) was prepared via solid-phase peptide synthesis with 99% purity (Supplementary Fig. 1). Then, cRGT was assembled in only two steps via expressed protein ligation (EPL) followed by disulfidization chemistry (Fig. 1d). First CysTMP was ligated with GBP-intein-CBD (Supplementary Fig. 2a, chimera **I**; CBD: chitin-binding domain) to attach TMP to the C-terminus of GBP along with cleavage of the intein-CBD tag (Supplementary Fig. 2b, chimera **II**) to produce GBP-TMP after purification (Supplementary Fig. 2c, conjugate **III**). Finally, GBP-TMP that bears an internal cysteine residue was coupled with Cys-cR10* via a disulfide bridge using disulfidization chemistry to create cR10*-SS-GBP-TMP, i.e. cRGT (Supplementary Fig. 2d, left, conjugate **IV**). The cR10* module can be rapidly and completely cleaved under reducing conditions at room temperature (Supplementary Fig. 2d, right).

Using size exclusion chromatography (SEC), we demonstrated that GBP-TMP induces dimerization between enhanced GFP (EGFP) and eDHFR in vitro. A stable EGFP/GBP-TMP/eDHFR ternary complex was formed in the presence of GBP-TMP while no complex was generated in the absence of GBP-TMP (Fig. 1e–g). We further validated the dimerization process using Förster resonance energy transfer (FRET) and confirmed the interaction between EGFP donor and mScarlet-eDHFR acceptor in the presence of GBP-TMP (Supplementary Fig. 3).

### The SNACIP inducer cRGT enables minute-scale, no-wash, reversible and dose-dependent control of protein positioning inside living cells

**Fig. 2:**
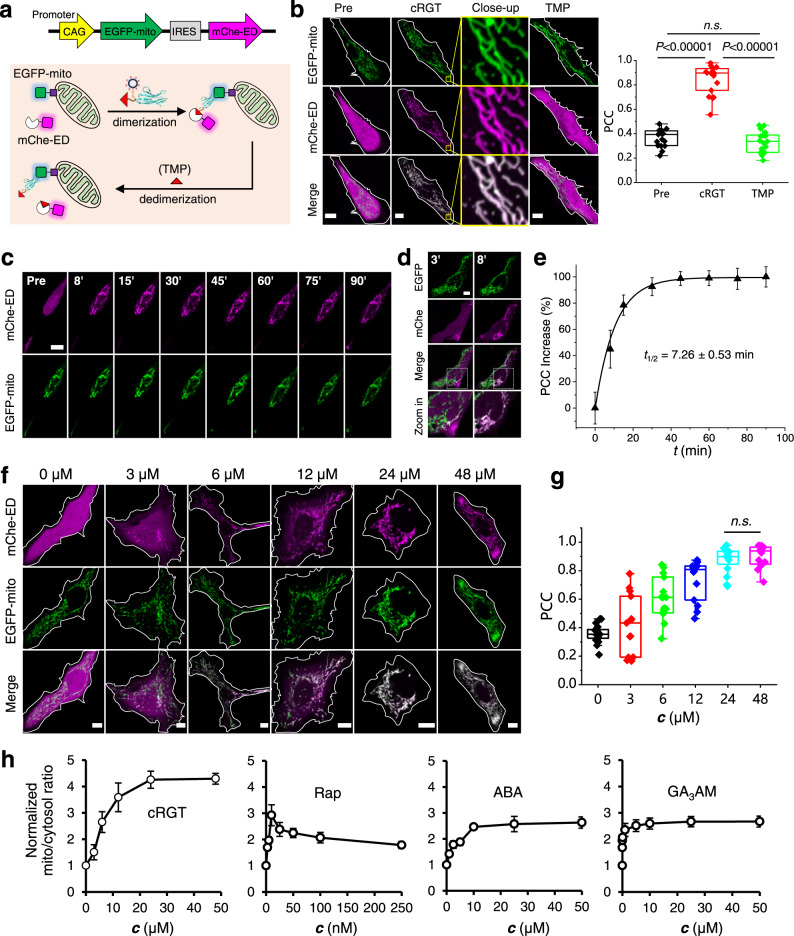
cRGT enables minute-scale, no-wash, reversible and dose-dependent control of dimerization between EGFP and eDHFR inside living cells. **a** Schematic view of the experimental design in which a bicistronic vector that co-expresses mCherry-eDHFR (red, cytosol) and EGFP-mito (green, mitochondria; mito: mitochondria targeting sequence) was used. **b** Representative confocal images of live HeLa cells coexpression of mCherry-eDHFR and EGFP-mito before adding cRGT (Pre), after adding 24 μM cRGT, for 1.5 h and after adding 10 μM TMP for 10 min; right: statistical PCC analysis between the two channels (*n* = 21 cells for Pre, *n* = 18 cells for cRGT, *n* = 19 cells for TMP); one-sided Student’s *t*-test was used; see Methods for description of box plots. **c** Live HeLa cells co-expressing mCherry-eDHFR and EGFP-mito were treated with 24 μM cRGT and the dimerization pattern was recorded over time. **d** cRGT started to penetrate cells in a polarized fashion within 3 min (yellow arrow) and prominent dimerization was achieved in 8 min. **e** Normalized PCC increase plotted against time revealed a *t*_1/2_ of 7.26 ± 0.53 min for cells treated with 24 μM cRGT (*n* = 7 cells); bar graphs denote mean ± standard error of the mean (SEM). **f** Live HeLa cells co-expressing mCherry-eDHFR and EGFP-mito were treated with increasing dosages of cRGT (0, 3, 6, 12, 24, and 48 μM) for 1.5 h, which induced stepwise increased degree of dimerization; dimerization reached maximal when 24 μM cRGT was reached. **g** Statistical PCC analysis between EGFP and mCherry channels (*n* = 19/13/15/14/16/15 cells for 0/3/6/12/24/48 μM); two-sided Student’s *t*-test was used; see Methods for description of box plots. **h** Comparison of cRGT with several state-of-the-art dimerizers using a translocation assay by treating live HeLa cells with gradient final concentrations of given dimerizers for 1.5 h. Live HeLa cells co-express a pair of dimerization domains (cRGT: mChe-ED/EGFP-mito; Rap: mChe-FRB/FKBP-EGFP-mito; ABA: mChe-ABI/EGFP-PYL-mito; GA_3_AM: mChe-GID/GAI-EGFP-mito) and the addition of a dimerizer will lead to the translocation of mCherry-tagged partner to mitochondria; bar graphs denote mean ± SEM. Cells analyzed: *n* = 19/13/15/14/16/15 cells for 0/3/6/12/24/48 μM in cRGT group; *n* = 11/9/12/11/10/12/10/11 cells for 0/2.5/5/10/25/50/100/250 nM in Rap group; *n* = 13/11/8/9/10/11/10 cells for 0/1/2.5/5/10/25/50 μM in ABA group; *n* = 15/15/15/15/12/14/13/15/15/15 cells for 0/0.001/0.005/0.01/0.1/1/5/10/25/50 μM in GA_3_AM group. mChe mCherry, ED eDHFR . All scale bars: 10 μm.

Cellular processes are often regulated by the dynamic distribution of molecules inside cells. Therefore, we first used cRGT for control of protein positioning. HeLa cells co-expressing EGFP-mito and mCherry-eDHFR were treated by 24 μM cRGT for 1.5 h and imaged without washing. cRGT directed mCherry-eDHFR to EGFP-mito at the mitochondria based on zoomed-in high resolution confocal images and Pearson’s correlation coefficient (PCC) colocalization analysis (Fig. 2a, b). This can be attributed to the formation of a stable EGFP/GBP-TMP/eDHFR ternary protein complex as shown above (Fig. 1e–g). Since TMP is a known reversible inhibitor of eDHFR, 10 μM TMP was added to the cell culture, which abolished the dimerization in 10 minutes (Fig. 2a, b). Dose-response study revealed that 5 μM of TMP have been sufficient to induce dedimerization (Supplementary Fig. 4).

We then studied the cellular entry mechanism and dimerization kinetics of cRGT. cRGT rapidly enters live HeLa cells in a non-endocytic fashion within a few minutes (Supplementary Fig. 5a–e). cRGT inside live cells shows no colocalization with endosomal puncta (Supplementary Fig. 5f) and the presence of an endocytic inhibitor using either chlorpromazine or dansylcadaverine does not prevent the cellular entry of cRGT (Supplementary Fig. 5g, h). These evidences further confirm the non-endocytic entry mechanism. cRGT began to induce dimerization as early as 3 min and a prominent dimerization pattern was observed by 8 min (Fig. 2c, d). Statistical kinetics revealed *t*_1/2_ = 7.26 ± 0.53 min (Fig. 2e) for cRGT to reach maximal degree of dimerization. Although being a macromolecule conjugate, this rate renders cRGT induced dimerization in the range of small-molecule based popular CIDs^26^. Cytosolic mCherry-eDHFR was recruited to mitochondria in a dose-dependent manner and 24 μM cRGT represent an optimal concentration (Fig. 2f–g). In contrast, GBP-TMP without bearing cR10* did not induce any intracellular dimerization, confirming that cR10* is indeed essential for intracellular delivery (Supplementary Fig. 6). EdU cell proliferation assay revealed that cRGT is not harmful to cells under the conditions used in this study (Supplementary Fig. 7). We compared cRGT with several state-of-the-art dimerizers including rapamycin (Rap), (+)-abscisic acid (ABA), and GA_3_AM using a translocation assay in which protein translocation can be quantified by measuring fluorescence intensity. According to the dose-dependent translocation curve (Fig. 2h), Rap shows a “Hook effect”, or so-called “off-on switch” effect^27^ with a narrow optimal concentration window, and this effect is not originated from potential Rap precipitation (Supplementary Fig. 8). Also, cRGT achieves a larger possible translocation dynamic range than ABA. Compared to GA_3_AM^5^, cRGT-induced dimerization can be readily reversed using TMP as shown before.

With cRGT in hand, we show that cRGT efficiently directs EGFP to different organelles including mitochondria (Supplementary Fig. 9a) and Golgi apparatus (Supplementary Fig. 9b) in a reversible fashion. Noteworthy, the small size of cRGT also allows it to control protein re-positioning into nucleus (Supplementary Fig. 9c, d). The yellow-color mEYFP and cyan-color mTurquoise2 represent other close variants of GFP. Positioning of mEYFP is controlled by cRGT without compromising dimerization efficacy but mTurquoise2 cannot (Supplementary Fig. 10a, b). This is interesting but understandable because Asn146, which forms a key hydrogen bond with Asn99 in GBP, is retained in EGFP and mEYFP but is replaced by Phe146 in mTurquoise2. We also used non-FP fused eDHFR-mito to exclude any artifacts originated from FP-FP interactions and confirmed that cRGT is orthogonal to other commonly used FPs ranging from blue to red including mTagBFP2, mTurquoise2, DsRed, mScarlet and mCherry (Supplementary Fig. 10c–h). Hence cRGT is a versatile player that modulates the positioning of EGFP, mEYFP and eDHFR fused POIs with orthogonality to other commonly used FPs.

### cRGT controls signal transduction by bringing the signaling protein Rac1 to the proximity of PM

**Fig. 3:**
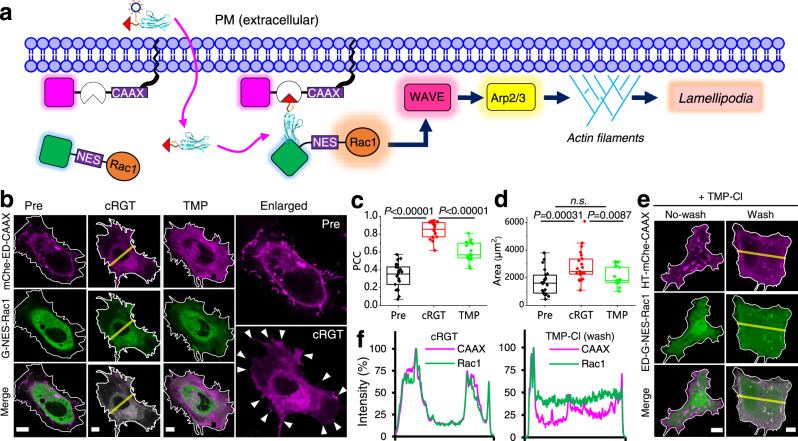
cRGT brings the signaling protein Rac1 to the proximity of inner PM to initialize the signaling cascade of lamellipodia formation. **a** A non-PM targeting and constitutively active EGFP-NES-Rac1Q61LΔCAAX (i.e. G-NES-Rac1) mutant with the truncation of its PM-targeting C-terminal CAAX-box was engineered and co-expressed with mCherry-eDHFR-CAAX in live HeLa cells; treatment of HeLa cells with cRGT will target Rac1 mutant to the functional PM location and initialize lamellipodia signaling pathway. WAVE WASp-family verprolin-homologous protein, Arp2/3 actin-related proteins-2/3. **b** Representative live HeLa cells coexpression of G-NES-Rac1 (green, cytosolic) and mCherry-ED-CAAX (red, PM) before adding cRGT (Pre), after treatment with cRGT (24 μM, 1.5 h), and after adding TMP (10 μM, 10 min); NES: nucleus exporting signal, LQNKLEELDL, which reduces Rac1’s accumulation in the nucleus. **c** Statistical PCC analysis between EGFP and mCherry channels (*n* = 22 cells for Pre, *n* = 21 cells for cRGT, *n* = 18 cells for TMP); one-sided Student’s *t*-test was used; see Methods for description of box plots. **d** Quantification of the cell areas before adding cRGT (Pre), after adding 24 μM cRGT, and after adding 10 μM TMP for 10 min (*n* = 22 cells for Pre, *n* = 21 cells for cRGT, *n* = 17 cells for TMP); one-sided Student’s *t*-test was used; see Methods for description of box plots. **e** Representative confocal images of a comparable CID (TMP-Cl, 10 μM, 1 h) induced targeting of eDHFR-EGFP-NES-Rac1Q61LΔCAAX (i.e. ED-G-NES-Rac1) to PM without washing (left) and after washing out (30 min) excess of TMP-Cl (right); HT HaloTag. **f** Comparison between cRGT and TMP-Cl induced dimerization using line profile analysis. All scale bars: 10 μm.

PM is the central signal hub of cells and the ability to bring signaling molecules to the proximity of PM is a general approach to control signal transduction^28^. As a proof of concept, we controlled Rac1-mediated signal transduction during lamellipodia formation, which plays a key role in metastasis and invasion of cancer cells^29^. We found that constitutively active Rac1 mutant can be readily targeted to PM by cRGT causing a subtle change of cell phenotypes (Fig. 3a, b). Cells show a highly extended shape with many newly formed lamellipodia (Fig. 3b, lower-right, white triangles). This process was fully reversed after applying 10 μM TMP for 10 min (Fig. 3b, c). The average cell area increased from around 1500 μm^2^ to 2500 μm^2^ after adding cRGT and decreased significantly after the addition of TMP (Fig. 3d). TMP-Cl which is the core element of several recently published TMP-tethered CIDs^14,28,30,31^, fails to induce dimerization without washing out TMP-Cl and induces less complete dimerization than cRGT after washing out TMP-Cl (Fig. 3e, f, Supplementary Fig. 11). Hence, cRGT can also be advantageous over related CIDs to study biological systems and to modulate targets present at low concentrations inside cells^32^.

### Study cargo-motor specificity by bringing a motor protein to the proximity of cellular cargo surface

**Fig. 4:**
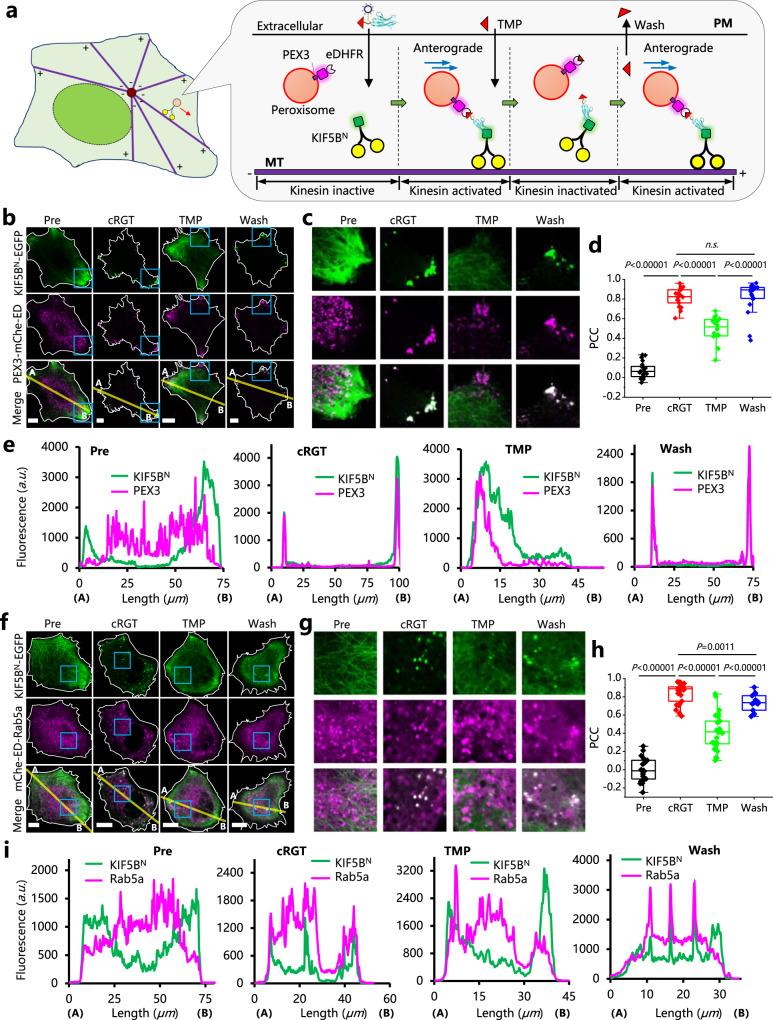
Multi-round reversible control of cellular cargo transport and study of kinesin-cargo specificity using cRGT-based SNACIP. **a** Schematic view of how cRGT in combination with TMP inhibitor reversibly controls KIF5B-mediated cellular transport of peroxisomes. In this process, attachment of the N-terminal motor region (1–560) of KIF5B, i.e. KIF5B^N^, to an appropriate cargo, e.g. peroxisome, can activate KIF5B^N^ and subsequently stimulate the anterograde transport of cargo toward the plus end of microtubules, usually located at the periphery region of a cell. **b** Representative confocal images of live HeLa cells co-expressing PEX3-mCherry-eDHFR (peroxisome localization) and KIF5B^N^-EGFP before adding cRGT (Pre), after adding 24 μM cRGT for 1.5 h, after adding 10 μM TMP for 10 min and after washing out TMP (10 min). **c** Zoomed-in images of (**b**, cyan squares) with details of individual peroxisomes displayed. **d** Statistical PCC analysis between red and green channels (*n* = 21 cells for Pre, *n* = 22 cells for cRGT, *n* = 20 for cells TMP, *n* = 21 cells for Wash); one-sided Student’s *t*-test was used; see Methods for description of box plots. **e** Line profile analysis of the images in **b**. **f** Representative confocal images of live HeLa cells co-expressing mCherry-eDHFR-Rab5a (early endosome localizer) and KIF5B^N^-EGFP before addition of cRGT (Pre), after adding 24 μM cRGT for 1.5 h, after adding 10 μM TMP for 10 min, and after washing out TMP (10 min). **g** Zoomed-in images of (**f**, cyan squares) with more details displayed. **h** Statistical PCC analysis between red and green channels (*n* = 22 cells for Pre, *n* = 30 cells for cRGT, *n* = 26 cells for TMP, *n* = 14 cells for Wash); one-sided Student’s *t*-test was used; see Methods for description of box plots. **i** Line profile analysis of the confocal images in (**f**) which revealed that colocalization was induced but no efficient anterograde transport of early endosomes to the very periphery of cells occurred. All scale bars: 10 μm.

Regulation of the proximity between cellular cargos and motors are essential for protein and organelle transport along microtubules^30^, proper functioning of neurons^33^ and checkpoint signaling during cell division^31^. We then demonstrated the usefulness of SNACIP to study motor-cargo interaction^34^. First, kinesin-mediated cargo transport could be regulated in a multi-round “OFF”→“ON”→“OFF”→“ON” fashion via washing to remove TMP, further highlighting the nice reversibility of SNACIP (Fig. 4a–c). Kinesin motor was completely targeted to and released from cargos, which enabled reversible control of anterograde transport of cargo toward the plus end of microtubules at the cell periphery (Fig. 4b–e). We compared two labeled cargos and noted that peroxisomes are more efficiently transported with KIF5BN-EGFP than early endosomes (Fig. 4f–i).

### cRGT activates ferroptosis by bringing GPX4 to the proximity of peroxisomes

Ferroptosis is a recently recognized nonapoptotic form of regulated cell death that is iron-dependent and characterized by the morphological change of mitochondria and the accumulation of lipid-based reactive oxygen species^35^. It is speculated that targeting ferroptosis is a promising way to kill therapy-resistant cancers because cancer cells exhibit an increased iron demand compared with normal, non-cancer cells^36^. Motivated by this, we aimed to use cRGT to activate ferroptosis. Among many ferroptosis factors, glutathione peroxidase 4 (GPX4) is considered as the key factor that protects biomembranes from peroxidation (Supplementary Fig. 12a). Additionally, a recent study using genome-wide CRISPR screens has identified peroxisome components, including PEX3, as contributors to ferroptosis susceptibility^37^. Therefore, we envisioned that targeting the ferroptosis suppresser GPX4 to the proximity of PEX3 on peroxisomes may inhibit GPX4 and activate ferroptosis (Supplementary Fig. 12b). We found that cRGT (24 μM, 2 h) treated live HeLa cell repositioned EGFP-GPX4 to PEX3-mCherry-eDHFR on the surface of peroxisomes along with a subtle morphological change of mitochondria and the cell. Smaller than normal mitochondria and condensed mitochondrial membrane densities were observed together with an abnormal phenotype of the cell (Supplementary Fig. 12c–e). These observations matched well with the most characteristic features of ferroptotic cells and suggested that cRGT has rapidly activated ferroptosis in cancer cells.

### Extension of the cRGT-type inducer to modulate other targets via rapid exchange of the nanobody module

The above-mentioned examples show that cRGT-based SNACIP represents a general tool for control of cellular processes. In fact, the SNACIP concept is not limited to regulate only EGFP variants or eDHFR fused proteins. For example, the GBP nanobody can be facilely replaced by other nanobodies to further extend the application potential. In order to demonstrate this possibility, we employed a mCherry red fluorescent protein binding protein (RBP) nanobody^38^. Setup the ligation between RBP-Intein and Cys-TMP requires less than 10 min and coupling of Cys-cR10* requires a few hours of work (Supplementary Fig. 13a, b). Hence, the SNACIP, cR10*-SS-RBP-TMP (i.e., cRRT), was assembled (Supplementary Fig. 13c) using no more than two days of work. cRRT behaves similarly to cRGT and it induces dimerization at a high colocalization degree after 1.5 h at 24 μM concentration; the dimerization degree was not compromised when higher concentration of cRRT (48 μM) was used (Supplementary Fig. 13d, e).

Further, not only the nanobody module, but also both the nanobody and TMP binding motif can be altered to extend the application potentials of SNACIP. Hence, we are motivated to introduce another SNACIP inducer cRTC for direct modulation of endogenous TPX2 with an aim to offer additional biomedical insights in microtubule nucleation.

### Design of a latent SNACIP inducer cRTC for modulating the endogenous unligandable microtubule nucleation factor TPX2 involved in cancer cell division

Endogenous unligandable proteins are challenging targets for traditional CIPs^26^. Among them, intrinsically disordered proteins (IDPs) are receiving increasing interest due to their vital roles in biology^39^. Also, microtubule nucleation is an important topic in the cytoskeleton field. Recently, structures of the microtubule nucleator, γ-tubulin ring complex (γ-TuRC), were resolved^40,41^, but structures of other essential modules, e.g. augmin complex and IDP factors, are still unavailable. The intrinsically disordered TPX2 protein that mediates the Ran pathway in spindle assembly is a key regulator in microtubule nucleation^42^; as an oncogenic protein, TPX2 is also overexpressed in many cancers including hepatocarcinoma^43^ which is among the most difficult cancers to treat^44^.

Post-translational modifications (PTMs) promote proximity of molecules and regulate protein’s activity, localization and interaction with other cellular molecules^45^. Inspired by this, we aimed to design a latent version of SNACIP to control TPX2. A latent SNACIP inducer employs PTM machinery to equip a small-molecule binding motif to convert it to a functional SNACIP inducer. This latent strategy can modulate endogenous proteins in non-genetically modified cells or organisms and hence could lead to opportunities for therapeutic intervention. Moreover, it facilitates the assembly SNACIPs with site-specific attachment of the cR10* moiety being the only major bioconjugation step.

**Fig. 5:**
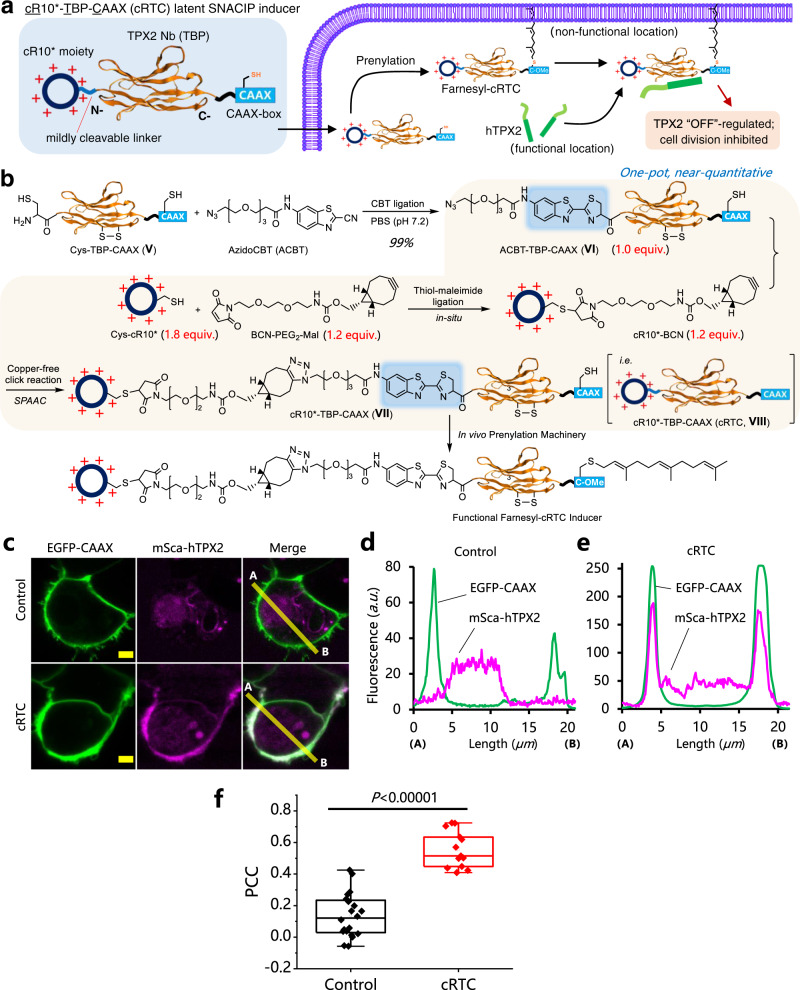
Design of cRTC inducer and its rapid assembly via N-terminal nanobody functionalization using stoichiometry-controlled one-pot tandem bioorthogonal ligations. **a** Structural elements of cRTC (left) and schematic presentation of how cRTC “OFF”-regulates the key microtubule nucleation factor TPX2 (right). **b** Schematic view of the assembly of cRTC inducer (BCN: bicyclonornyne which reacts with azide via copper-free click reaction; Mal: maleimide which reacts with cysteine). **c** Live HepG2 cells coexpression of EGFP-CAAX (PM marker) and mScarlet-hTPX2 (hTPX2 marker) with or without (control) adding 10 μM cRTC (1.5 h) reveal the repositioning of hTPX2 to PM. **d**, **e** Line profile analysis of the cells shown in **c**. **f** Statistical PCC analysis between EGFP-CAAX and mScarlet-hTPX2 for cells in **c** (*n* = 20 cells for Control, *n* = 13 cells for cRTC); one-sided Student’s *t*-test was used; see Methods for description of box plots. Yellow scale bars: 5 μm.

Positioning a molecule to the proximity of PM has also been elegantly used by nature to deactivate cellular activities. For example, the cytosolic DNA sensor cGAS, which is active as an immune receptor in cytosol, will be converted to a rest state when positioned at PM^46^. Inspired by this, we designed and assembled the latent SNACIP inducer cR10*-TBP-CAAX, or cRTC, that features a human TPX2 (hTPX2) binding protein (TBP) nanobody in the middle, a cyclic cR10* at the N-terminus, and a C-terminal CAAX-box sequence (Fig. 5a, left). The CAAX-box undergoes S-prenylation inside live cells to equip a farnesyl group for binding with PM^47^. Once cRTC penetrates cells, cRTC will be converted to a functional farnesyl-cRTC SNACIP and brings endogenous hTPX2 to the proximity of inner PM lipid bilayer. Hence, hTPX2 is recruited to the “rest”-PM position, deactivated, and subsequently inhibit cell proliferation (Fig. 5a, right).

### One-pot assembly of cRTC from azido-functionalized TBP-CAAX via tandem bioorthogonal ligations for “OFF’-regulation of hTPX2

Using M13 phage display, we generated a nanobody against hTPX2, i.e. TBP, that binds strongly with hTPX2 at nanomolar range (Supplementary Fig. 14a). We generated a TBP-mCherry chromobody and demonstrated that TBP indeed specifically target hTPX2 using live-cell immunofluorescence staining (Supplementary Fig. 14b, c). Then, we successfully assembled cRTC in one-pot via tandem bioorthogonal ligations using several bifunctional bioorthogonal linkers (Fig. 5b). Using this approach, the free cysteine residue in the CAAX-box that is a requisite for prenylation was kept intact during the entire ligation course. First, Cys-TBP-CAAX (**V**) that bears a free N-terminal cysteine was coupled with the bifunctional azidoCBT (ACBT) via CBT ligation to yield ACBT-TBP-CAAX (**VI**) (Supplementary Fig. 15a, b). Meanwhile, Cys-cR10* was ligated in situ with BCN-PEG_2_-Mal bifunctional linker via Michael addition to produce cR10*-BCN which was subsequently attached to ACBT-TBP-CAAX (**VI**) via strain-promoted azide-alkyne cycloaddition (SPAAC) in a one-pot fashion. The overall ligations rapidly assembled cRTC within 24 hours (Supplementary Fig. 15c, conjugate **VII**). Of note, the CBT moiety in cRTC is intrinsically florescent, facilitating the analysis of intracellular positioning and trafficking of cRTC.

cRTC inducer clearly targeted cytosolic hTPX2 to the PM region in live HepG2 cells (Fig. 5c–f). We mutated only Cys17 in the CAAX region and found that TBP-CAAX completely lost its ability to target PM, supporting that Cys17 in the CAAX-box of cRTC undergoes prenylation to transform cRTC into the functional farnesyl-cRTC (Supplementary Fig. 16).

### hTPX2 SNACIPs inhibit cell proliferation and suppress tumor growth in vivo

**Fig. 6:**
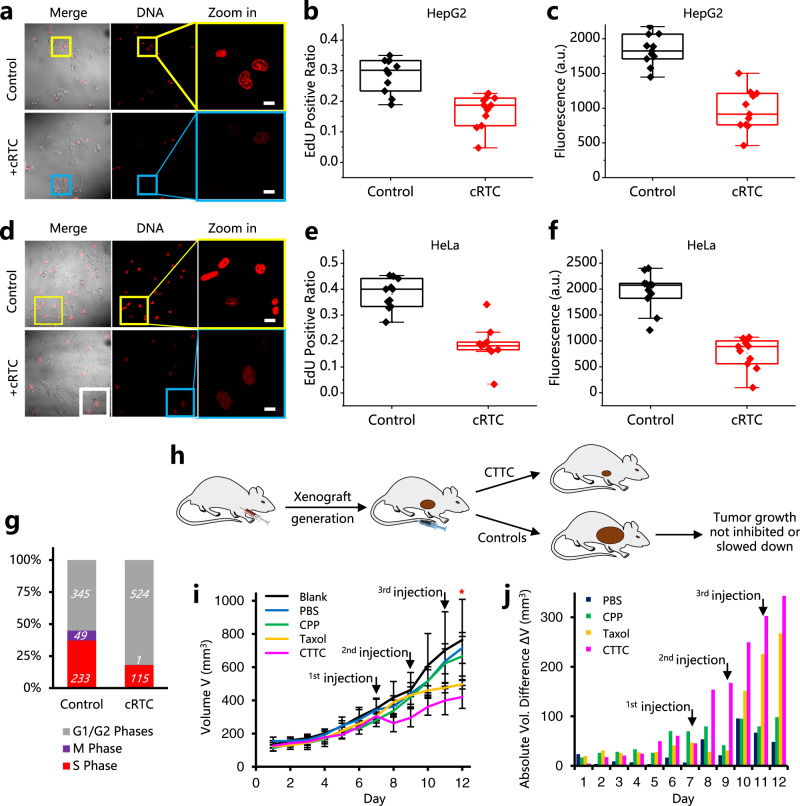
TPX2 SNACIP inducers effectively inhibit cancer cell proliferation via blocking cell cycle progression to M-phase, and suppress hepatocarcinoma cell tumor growth in vivo. **a** Representative confocal images of cRTC-treated (+cRTC, 10 μM, 24 h) and control HepG2 cells in the EdU cell proliferation assay. **b** Statistical analysis of EdU positive ratios of HepG2. **c** Statistical analysis of fluorescence intensities of EdU positive HepG2 cells. **d**–**f** Same experiments as in (**a**–**c**) for HeLa cells. *n* = 12 cells in **b**–**c** & **e**–**f**, and see Methods for description of box plots for **b**–**c** & **e**–**f**. **g** Percentage of each cell cycle phase of HeLa cells with or without adding cRTC; the total cell number in all imaging fields (*n* ≥ 10) at each cell cycle was given. **h** Generation of hepatocarcinoma xenograft mouse model by subcutaneous injection of ~5 × 10^6^ HepG2 cells into the axillary region of BALB/c nude mice to evaluate CTTC tumor suppression efficacy. When tumors had grown to between 0.7 cm and 0.9 cm in diameter, CTTC was injected intravenously via the tail vein, and the injection was repeated every other day to compensate for metabolic consumption of CTTC in vivo. **i** Tumor volumes of CTTC-injected, CPP-injected, PBS-injected, Taxol-injected, and the blank groups were measured every day; error bars: SEM (standard error of the mean, *n* = 3–6); one-sided Student’s *t*-test; *P = 0.039 for CTTC-injected compared to PBS-injected group (*n* = 4 mice for Blank, *n* = 6 mice for PBS, *n* = 3 mice for CPP, *n* = 4 mice for Taxol and *n* = 3 mice for CTTC from 2 independent experiments; bar graphs denote mean ± SEM). **j** Absolute tumor volume differences for the average value of different groups compared to the blank group suggested that only CTTC-injected and Taxol-injected mice showed tumor growth suppression. All scale bars: 10 μm.

We next asked if “OFF”-regulation of hTPX2 using cRTC inhibits cell proliferation or not using the EdU proliferation assay. cRTC-treated HepG2 cells showed reduced EdU positive ratio (Fig. 6a, b) and reduced brightness in the nucleus (Fig. 6a, c) while even greater reduced proliferation activities were observed for HeLa cells (Fig. 6d–f). We further analyzed different phases in the cell cycle. S-phase cells are essentially the EdU positive cells while mitotic phase (M-phase) cells are characterized by the unique phenotypes (dumbbell or round-shaped). We were therefore able to draw a diagram of cell phase changes of HeLa cells, which showed that S-phase ratio decreased significantly while M-phase was almost completely disappeared after cRTC treatment (Fig. 6g). Hence cRTC suppressed cancer cell division via blocking cell cycle progression to M-phase.

Based on the results above, we predicted that hTPX2 SNACIPs can be developed as SNACIP therapeutics to suppress tumor growth in vivo. To make cRTC better suited for in vivo evaluation, we designed and prepared a bivalent version latent SNACIP inducer mCherry-CPP-TBP-TBP-CAAX (CTTC) which features a tandem TBP nanobody (TBP-TBP) and a Tat CPP (Supplementary Fig. 17a). A bivalent nanobody moiety typically allows enhanced antigen avidity and an extended plasma half-life^48^. CTTC also readily enters the HeLa cell, directs hTPX2 to the non-functional PM region (Supplementary Fig. 17b) and effectively suppresses cell proliferation (Supplementary Fig. 17c, d). Prior to in vivo evaluation, we further showed that the TBP-TBP bivalent nanobody, either fused with C-terminal CAAX-box or not, interacts with endogenous TPX2 using immunoprecipitation (IP) (Supplementary Fig. 18).

Using a hepatocarcinoma xenograft mouse model (Fig. 6h), we found that tumors in the control group (PBS) grew as rapidly as in the blank group (Fig. 6i, j). For mice subjected to CTTC injection, tumor volumes decreased 24 h post drug-injection and tumor growth was still inhibited over a longer time period compared to blank and control groups (Fig. 6i, j). As a positive control, Taxol also displayed some tumor suppression effect, albeit with a slower onset (Fig.6i, j, orange line or column). We also designed and prepared a non-SNACIP form bivalent nanobody chimera CTT which cannot be prenylated. Using a same batch of mice, CTTC clearly showed a higher tumor suppression effect than CTT (Supplementary Fig. 19). These in vivo results highlight the value of SNACIP in modulating endogenous undruggable targets for drug development.

## Discussion

In summary, we introduced small molecule-nanobody conjugate inducers of proximity (SNACIPs) as attractive additions to currently existing sets of CIP and CID molecules. The presence of a nanobody binding module enables direct modulation of FP-fused proteins or endogenous targets. In this study, we showcased two major classes of SNACIPs. The first is the general-purpose SNACIP as exemplified by cRGT that features a small-molecule TMP ligand and is able to induce heterodimerization between a FP-fused protein and an eDHFR-fused protein. The presence of a reductively cleavable cyclic decaarginine CPP allows the efficient transduction of cRGT-type dimerizers into live cells. The second is the latent-type SNACIP which employs post-translational modification to equip the small-molecule binding motif; hence this strategy facilitates the preparation and can also skip genetic modification of cells.

The general-purpose cRGT inducer achieves minute-scale, no-wash, reversible and dose-dependent control of dimerization inside living cells. Therefore, it turns to be a useful dimerizer and has been demonstrated to control several cellular processes including protein positioning, signal transduction, cargo transport and ferroptosis. In particular, temporally resolved results showed that ferroptotic phenotypes were rapidly induced by bringing GPX4 factor to the proximity of peroxisomes, and this approach may serve as a valuable mean to induce cancer cell death. Also, the nanobody module could be exchanged to further expand the substrate scopes, as exemplified by the preparation of cRRT that dimerizes a mCherry-fused protein and an eDHFR-fused protein.

The development of microtubule nucleation modulators for cancer therapy has been proposed for decades since the identification of microtubule nucleation^49^. These nucleation factors are non-cell surface targets and are mostly disordered or form large protein complexes. Hence, they represent difficult targets for both antibody modalities and small molecule inhibitors. For this, we introduced latent-type SNACIP inducers that can directly modulate unligandable factors inside cells. We showed that latent cRTC and the modified versions were able to modulate an essential unligandable nucleation factor called hTPX2 for inhibiting cancer cell proliferation and tumor growth in vivo. This may create a route for the development of potent agents to treat cancers, which will be advantageous over traditional MTAs that often lead to a condition known as chemotherapy-induced peripheral neuropathy (CIPN)^50^.

SNACIP methodology may also associate with few limitations. Unlike CIPs, preparation of SNACIPs requires attachment of both a small molecule ligand and an appropriate cell-penetrating peptide using bioorthogonal ligations^51^. In this study we showed a few ways to install a small molecule/peptide moiety onto the C-terminus, N-terminus or a side chain of a nanobody, which could be helpful to guide the preparation of additional SNACIPs. The requirement of a nanobody may also represent a challenge in some circumstances. Along with the development of contemporary nanobody screening and evolution technologies^17,52^, enrichment of public nanobody libraries, and more available commercial sources, this potential challenge may gradually become a less critical concern in the future. Sometimes nanobodies may affect biological processes via, for example, block binding sites via steric hindrance; this could be circumvented by screening nanobody candidates that not do not interfere with biological functions.

With respect to the above-mentioned findings, we expect that SNACIPs will be next generation proximity inducers for control of various cellular processes. Given the current development of protein degradation modalities^53^, optogenetic and chemogenetic tools^54,55^, and cell therapies^56^, we envisioned that SNACIP methodology may also be extended for targeted protein degradation, spatiotemporal control of cellular functions, and proximity-enabled cell therapeutic applications in the future.

## Methods

### Ethical statement and animal welfare

Mice were maintained under specific pathogen free (SPF) conditions and handled under the approval from the Institutional Animal Care and Use Committee (IACUC) of Harbin Institute of Technology with the permit number IACUC-2021052. Mice were housed under controlled light (12 h light/12 h dark cycle), temperature (24 ± 2 °C) and humidity (50 ± 10%) conditions with a free access to autoclaved chow and water. The maximal tumor size permitted by IACUC is 20 mm for the length, or 10% of the body weight, and this limit was not exceeded in the study.

### Mammalian cell culture

HeLa (Cat# CL-0101) and HepG2 (Cat# CL0103) cells were obtained from Procell Life Science & Technology Co., Ltd. (Wuhan, P.R. China), short tandem repeat (STR) identified and proven to be HIV-1, HBV, HCV, mycoplasma, and other microorganisms free before culturing. Other reagents such as full DMEM (Dulbecco’s modified Eagle’s medium) and PBS (phosphate buffered saline) were also confirmed to be mycoplasma free before usage. Cell culture were maintained at 37 °C under 5% CO_2_ in high glucose (4.5 g · L^−1^) DMEM (Cat# SH30243.01, HyClone) containing 4 mM L-glutamine and sodium pyruvate and supplemented with additional 10% fetal bovine serum (FBS, Cat# SV30087.03, HyClone), 1% non-essential amino acid (NEAA, 100×), and 1% penicillin-streptomycin (100×). Trypsin-EDTA (Cat# SH30042.01, HyClone) and PBS (Cat# SH30256.01, HyClone) were used in subculturing. HeLa cells were subcultivated in a ratio of 1:5–10 while HepG2 cells were subcultivated in a ratio of 1:4–6.

### Generation of xenograft mouse model and drug treatment

The immunodeficient BALB/c nude female mice about 4–6 weeks were obtained from Liaoning Changsheng Biotechnology Co., Ltd. HepG2 cells were grown in a standard *Φ* ~85 mm Petri dish to exponential phase before harvesting. Sex was not considered in the study design because hepatocarcinoma is not a gender-based disease. Then HepG2 cells were first washed by 10 ml PBS, added 1 ml of trypsin, digested for 5–10 min to allow full detachment of cells from the growing surface, and added 3 ml of PBS to suspend the detached cells. The cell suspension was subjected to centrifugation at 800 × *g* for 8 min at 4 °C. The clear supernatant was discarded and the cell pellets were resuspended in a freshly prepared ice-cold 1:1 (v/v) mixture of PBS/Matrigel (Cat# M8370, Solarbio) on ice. The final cell density is ~5 × 10^7 ^ml^−1^. To make HepG2 xenograft mouse model, ~5 × 10^6^ HepG2 cells in 0.1 ml of PBS/Matrigel solution were subcutaneously injected into the axillary region of the BALB/c nude mice. Tumor will typically appear in 1–2 weeks and will continue to grow steadily. In order to evaluate the efficacy of TPX2 SNACIP inducer, pH 7.2 PBS buffer supplemented with additional 1 mM TCEP, 0.5 M NaCl, and 3% glycerol was used as the control or to solubilize CTTC. Unless otherwise specified, 100 μl of 25 mg·ml^−1^ CTTC (M.W. 62.2 kD, 0.4 mM) in PBS, CPP (0.4 mM) in PBS or PBS alone (control) were injected intravenously via the tail vein to the mice and the injection was repeated at the given time point. For giving Taxol (Paclitaxel from Macklin, Cat# P875571), Paclitaxel was first dissolved in a 1:1 (v:v) mixture of EtOH/ Cremophor EL (Macklin, Cat# C804845) at 22.5 mg·ml^−1^. Then 30 μl of this solution were mixed with equal volume of EtOH/ Cremophor EL (1:1) and 240 μl of PBS to give 2.25 mg·ml^−1^ of Taxol diluted in PBS. Then around 80 μl of such Taxol solution was intravenously injected into a nude mouse (~18 g) so that the drug dosage was around 10 mg·kg^−1^ per each injection. Average tumor sizes were monitored every day using a caliber [*Φ* = (*Φ*_L_ + *Φ*_S_)/2]. Tumor volumes were calculated based on the following equation: V = 1/6(π*Φ*^3^).

### Plasmid construction

Plasmid vectors, such as pTXB1, pET28a(+), EGFP-C1 and EGFP-N1 were obtained from commercial vendors. These parental vectors may be further engineered, such as introducing a His_6_- or His_8_- affinity tag, insertion of a TEV or TEV’ protease cleavage site, alternation of restriction cleavage sits, or replacing EGFP by mTagBFP2, mTurquoise 2, mEYFP, mScarlet, or mCherry etc. to give modified versions of the parental vector for cloning. Subcloning, Gibson cloning, or modified Gibson cloning methods were employed to construct the desired plasmids. For subcloning, fragments of interest were directly cut from the parent plasmid using appropriate restriction enzymes, or amplified by PCR from plasmids containing the desired genes using hyPerFUsion high-fidelity polymerase (Cat# 1032, APExBIO), gel purified, digested with restriction enzymes and purified again. The gene fragments were ligated into appropriate vectors using T4 DNA ligase. Multiple fragments were assembled by stepwise subcloning or one-step multi-fragment Gibson cloning. List of primers for cloning plasmids that involve PCR amplification was provided as Supplementary Data 1.

Most genes were obtained via custom gene synthesis from Comate Bioscience Co., Ltd. (Changchun, P.R. China). These genes include *E. coli*. codon optimized *homo sapiens* TPX2 (TPX2), *E. coli*. codon optimized GFP nanobody (GBP), mScarlet, and etc. None-codon optimized TPX2 gene was amplified from the plasmid pLenti-EF1a-EGFP-P2A-Puro-CMV-TPX2-3Flag purchased from (Shanghai) Obio Technology Co., Ltd. (Cat# H10559). Plasmids containing human KIF5B, Rac1, Rab1b, Rab5a, and other genes were purchased from Miaoling Plasmid Sharing Platform. Mito-sequence: ESGDASGSGSGSRAQASNSKLIAKSAEDEKAKEEPGNHRIVILAMLAIGVFSLGALIKIIQLRKNN. CAAX-box sequence: KMSKDGKKKKKKSKTKCVIM.

### Transfection

Transient transfection was typically performed in an 8-well (Cat# 155409) or 4-well (Cat# 155382) Lab-Tek®II imaging chamber from Thermo Scientific. Typically, 0.25 μg DNA was dissolved in 12.5 µl gibco opti-MEM (Cat# 31985-062, Life technologies) while 0.5 μl ExFect®2000 transfection reagent (Cat# T202, Vazyme Biotech Co., Ltd., Nanjing, P.R. China) was dissolved in 12.5 μl gibco opti-MEM. Both solutions were first incubated at room temperature for 5 min. Then the diluted DNA solution in opti-MEM was transferred into ExFect®2000 solution in opti-MEM and mixed gently. The DNA/ExFect®2000 mixture in opti-MEM were further incubated at room temperature for 5–10 min (typically 7.5 min) and then gently added into an imaging chamber well freshly seeded with 1.5–2.0 × 10^4^ cells in 250 μl full DMEM. The cells were maintained under 5% CO_2_ at 37 °C for around 2 h to allow cell adhesion. Afterwards, the medium was replaced by warm full DMEM and the cells were further incubated under 5% CO_2_ at 37 °C for over 20 h. For co-transfection of more than one plasmid, the quantity of DNA used in this protocol implies the total amount of plasmids.

### Confocal microscopy

Live cells were imaged in phenol red free Dulbecco’s Modified Eagle Medium (Cat# 21063-29, Life Technologies) supplemented with additional 10% FBS, 1% sodium pyruvate, 1% NEAA, 1% penicillin-streptomycin and 15 mM HEPES-Na (final pH 7.0) at 37 ^o^C under 5% CO_2_. Microscopy was performed using Zeiss LSM 880 inverted confocal laser scanning microscope equipped with an Airyscan super resolution module. Zeiss Plan-APOCHROMAT 100×/1.4 oil DIC objective was primarily used for imaging while Zeiss Plan-APOCHROMAT 60×/1.4 oil DIC objective was used as an alternative; for larger field of views, Zeiss Plan-APOCHROMAT 40×/0.95 DICIII objective was used, such as in EdU assay. Confocal images were typically acquired in 12-bit depth at 512 × 512 resolution. 405 nm laser diode was used to excite mTagBFP2, DAPI, or Hochest, 458 nm argon laser was used to excite mTurquoise2, 488 nm argon laser was used to excite EGFP or fluorescein, 514 nm argon laser was used to excite mEYFP, HeNe laser 543 nm was used to excite Apollo 567 dye in EdU assay, and HeNe laser 543 nm or HeNe laser 594 nm was used to excite mScarlet or mCherry. In most cases, the basic imaging setup parameters were configured applying the “Smart-Setup” function with typical parameters set as follows: scan speed 8, pixel dwell time 1.54 μs, number of averaging 4, line mode in one-direction scanning, and pinhole 89.9 μm. Where appropriate, edges of live cells recorded in microscopy were sketched as white lines for better clarity in this study.

### Treatment of live cells with SNACIP inducers in microscopic imaging

Unless otherwise specified, the full DMEM was replaced by a solution of SNACIP inducer at the given concentrations in phenol red free imaging medium for a given period of time. The concentrations of cRGT mentioned through the text indicate the cR10*-bearing portion (**III**) in the cRGT product; and washing was not applied before imaging, which will already induce high-degree of dimerization. To reversibly control dimerization, a freshly prepared TMP (10 μM) solution in phenol red free imaging medium was used to replace the SNACIP inducer-containing imaging medium. Full dedimerization will be accomplished rapidly in 10 min. For inducing dimerization again, cells were rinsed by PBS (2×), incubated in imaging medium for 10 min, and rinsed again by PBS (1×) to fully remove TMP; subsequently imaging medium was added for microscopic imaging. For TMP-Cl induced dimerization, live HeLa cells were treated with 10 μM TMP-Cl for 1 h, rinsed with PBS (2×), incubated in imaging medium for 30 min, and rinsed again with PBS (2×); subsequently imaging medium was added for microscopic imaging.

### Immunofluorescence (IF) staining

HeLa cells were washed three times by PBS, fixed using 4% paraformaldehyde for 20 min, washed three times by PBS, permeabilized using 0.5% Triton X-100 in PBS for 20 min, washed three times by PBS, blocked with 5% BSA in PBS at room temperature for 30 min. Blocking solution was removed and primary antibody against hTPX2 (Cat# R27376, ZENBIO) diluted 1:100 in 0.5% BSA/ PBS was added and cells were incubated at 4 °C overnight. Next day, primary antibody solution was removed and then cells were incubated with Alexa Fluor 488 labeled secondary antibody (Cat# 550037, ZENBIO, 1:100 diluted in 5% BSA/ PBS) at room temperature for 1 h. Secondary antibody was removed and cells were washed three times by PBS and imaged under a confocal microscope.

### Immunoprecipitation (IP)

HeLa cells grown in a *Φ* 10 cm petri dish transfected with EGFP-TBP-TBP-CAAX or EGFP-TBP-TBP were harvested after tryptic digestion (~6 × 10^6^ cells/ dish), washed once with PBS, and lysed using 1 ml radioimmunoprecipitation assay buffer (RIPA) buffer (Cat# BL504A, Biosharp) supplemented with additional 1% PMSF on ice for 10 min. The cell lysate was then centrifuged at 17,320 × *g* at 4 °C for 10 min and the supernatant was separated for IP. IP-grade Protein A/G MagBeads (Cat# 36417ES03, Yeasen) were coated with EGFP antibody (Cat# R24437, ZENBIO) or IgG (Cat# A00002, ZENBIO) according to the manufacturer’s protocol. Beads were retrieved using a magnetic particle concentrator (MPC), gently mixed with the freshly prepared cell lysate supernatant, and were slowly rotated at 4 °C overnight. The next day, beads were retrieved using MPC, washed three times by 1× TBST, and boiled with 50 μl 2× SDS-PAGE sample loading buffer at 90 °C for 5 min. Then beads were removed using MPC and the supernatant was subjected to Western blot (WB) analysis. For WB, 10 μl of the sample was separated using SDS-PAGE (10 %), transferred to a nitrocellulose membrane, and probed with appropriate antibodies.

### EdU cytotoxicity/cell proliferation assay

The EdU cytotoxicity/cell proliferation assay was performed using an EdU cell proliferation detection kit (Cat# R11053.9, RiboBio). Briefly, 5 × 10^4^ HepG2 or 2 × 10^4^ HeLa cells harvested at exponential phase were seeded in an 8-well imaging chamber and allowed to grow overnight. Drug solutions at specified final concentrations in full DMEM was used to treat the cells. After a given incubation time, the drug solution was replaced by EdU solution at a final concentration of 50 μM. It was incubated for 2 hours at 37 °C under 5% CO_2_ as recommended for general cancer cell lines. Then, each well was washed by PBS (2 × 5 min) to remove the excess of EdU, added with 100 μl of fixative solution (4% PMA in PBS) and incubated for 30 min at room temperature. Then 100 μl of 2 mg·ml^−1^ glycine solution was added to each well and shaken at RT for 5 min to quench the fixative. Glycine solution was removed and each well was washed by 200 μl PBS and shaken at RT for 5 min. PBS was removed and each well was added with 200 μl cell permeabilization solution (0.5% TritonX-100 in PBS) and shaken at RT for 10 min. The fixed cells were further washed by PBS (1 × 5 min) before labeling.

Before fluorescent labeling by click reaction, 1× Apollo labeling solution that contains the red color Apollo567 dye (Cat# C10310-1, RiboBio), catalyst and other necessary reagents were freshly prepared according to the manufacture’s guidance. For example, 1 ml of 1× Apollo labeling solution could be prepared by sequentially adding 938 μl DI-H_2_O, 50 μl Apollo reaction buffer (reagent B), 10 μl Apollo catalyst solution (Cu^2+^, buffer C), 3 μl Apollo 567 dye (reagent D) and ~9 mg Apollo additive (sodium ascorbate, reagent E). 200 μl freshly prepared 1× Apollo labeling solution was added into each well, shielding from light, and shaken at RT for 30 min to complete the click labeling. Labeling solution was removed, and the cells in each well were washed by permeabilization solution (0.5% TritonX-100 in PBS) again (3 × 10 min). Permeabilization solution was removed and the cells were wash by PBS (1 × 5 min). Finally fresh PBS was added and the labeled cells were ready for confocal microscopy imaging. Hoechst could be used to label the nucleus if necessary.

### Isothermal titration calorimetry (ITC)

ITC was performed using MicroCal ITC200 from GE Malvern. TBP and hTPX2 were all dissolved in freshly prepared pH 7.2 PBS buffer supplied with additional 1 mM TCEP as reductant, 0.5 M NaCl and 3% glycerol. 21 μl TPX2 at 20 μM concentration was loaded into the sample cell. TBP at 51 μM concentration was loaded in the syringe and then injected in 2.0 μl × 18 portions to the sample cell at 25 °C with 3 min interval between each injection; only for the first injection, 0.8 μl nanobody was used followed by 2.5 min interval. The titration data was processed using the default Origin software.

### Förster resonance energy transfer (FRET)

EGFP and mCherry or mScarlet are FRET pairs due to the spectra overlap between the emission spectrum of them. The Molecular Device (MD) *SpectraMax i3x* spectrometer was used in FRET measurement. 100 μl or 200 μl solution of a mixture of donor and acceptor fluorophores were added into each well in the black 96-well plate. The excitation wavelength was set at 470 nm and the fluorescent spectra were recorded from 490–750 nm.

### Design and preparation of the cyclic cell-penetrating peptide Cys-cR10*

The cyclic Cys-cR10* peptide was designed with a cyclic rR ring (r = D-Arg, R = L-Arg) plus a (Gly)_5_ linker with a N-terminal free cysteine and a C-terminal -CONH_2_ group. The peptide was synthesized via standard solid phase peptide synthesis using Rink amide resin. After the synthesis of liner R10* fragment, intramolecular cyclization was performed to bridge the Lys side chain (-NH_2_ group) and Glu side chain (-COOH group). Afterwards, Cys-(Gly)_5_ tail was sequentially added to the cyclic-R10* moiety followed by TFA deprotection and HPLC purification. Cys-cR10* peptide was obtained in a high purity of 98.8% and confirmed using mass spectrometry. C_84_H_160_N_50_O_19_S, exact mass: 2205.28, M.W.: 2206.56; found *m/z* 736.4 [M + 3H]^3+^, 552.6 [M + 4H]^4+^, 442.3 [M + 5H]^5+^.

### Protein expression and purification

#### General protocol

pET28a(+) or modified pET28a(+) vectors were used in most protein expressions while pTXB1 vector was used to express intein-tag fused nanobody chimeras for expressed protein ligation (EPL). These plasmids for protein expression were first transformed into *E. coli* Rosetta 2a cells and the transformants were selected on ampicillin (100 mg·L^−1^) or kanamycin (50 mg·L^−1^) agar plates depending on the antibiotic resistance of the plasmids. A single colony was used to inoculate 50–100 ml of LB medium containing 100 mg·L^−1^ ampicillin or 50 mg·L^−1^ kanamycin and shaken at 240 rpm for 8–10 hours or overnight at 37 °C. 30–50 ml of the preculture was used to further inoculate ~1.8 L fresh LB medium containing 100 mg·L^−1^ ampicillin or 50 mg·L^−1^ kanamycin, and additional chloramphenicol (33 mg·L^−1^). The absorbance at 600 nm (OD600) of the inoculated culture should be controlled between 0.05 to 0.1 in this inoculation step. Then the culture was shaken at 180 rpm at 37 °C for a few hours (typically 2–3 h) until OD 600 reached 0.5–0.6. Then 0.5 ml isopropyl β-D-thiogalactoside (IPTG) stock solution (1 M) was added (final ~0.27 mM) to induce protein expression at 37 °C for 5 h, or at 16 °C overnight. Sometimes protein expression time and temperature needed to be optimized in order to achieve an optimal expression for some particular proteins.

Later, cells were harvested by centrifugation at 13,881 × *g*, at 4 °C for 15 min and washed once with PBS (4149 × *g*, 10 min). The bacterial pellet was resuspended in lysis buffer (pH 8.0, PBS supplemented with additional 0.5 M NaCl, 3% glycerol, w/o 3 mM β-mercaptoethanol (BME), 1 mM phenylmethylsulfonyl fluoride (PMSF). For relatively smaller volumes of bacterial cell suspensions (<40 ml), bacterial cells were typically lysed via ultra-sonification at 80 W for 30 min or 60 W for 45 min (1 s sonification followed by 3 s interval) on ice. For batch processing or larger volumes of cell suspensions, cells were typically lysed using ultra-high-pressure homogenizer cooled by a bench chiller for 2–3 cycles under 800–900 bar at 4 °C. The lysate was cleared by high-speed centrifugation (74,766 × *g*, 45 min, 4 °C) and the supernatant was loaded onto a gravity Ni-NTA column (2-5 ml resin). The Ni-NTA column was washed and then the His-tag fused protein was eluted using step-gradient of imidazole (50, 100, …, until 500 mM) solutions. Alternatively, GE ÄKTA Pure machine equipped with a HisTrap FF column was used to purify His-tagged protein via gradient elution (0 → 500 mM imidazole) by combining buffer A (pH 8.0 PBS, 0.5 M NaCl, 3% glycerol, w/o 3 mM BME) and buffer B (pH 8.0 PBS, 0.5 M imidazole, 0.5 M NaCl, 3% glycerol, w/o 3 mM BEM). Ionic exchange or size exclusion chromatography may be further applied if additional purifications are necessary. The obtained proteins were typically concentrated, buffer exchanged in buffer A, aliquoted, snap frozen in liquid nitrogen, and stored under −80 °C.

#### Preparation of hTPX2 antigen via TEV protease cleavage

hTPX2 could not be expressed without fusion with additional tags in *E. coli*. Therefore, a pET28b(TEV)_hTPX2-TEV-EGFP-His_8_ plasmid was first constructed, which features a C-terminal His_8_-tag, an EGFP tag at the C-terminus of hTPX2, and a TEV cleavage site. hTPX2-TEV-EGFP-His_8_ was expressed in *E. coli*. culture according to the above-mentioned general protocols. More specifically the respective Rosetta 2a *E. coli* culture was allowed to grow at 30 ^o^C overnight after adding IPTG. After cell pelleting, cell lysis, centrifugation and gradient Ni-IMAC purification, hTPX2-TEV-EGFP-His_8_ was obtained as a solution in pH 8.0 Buffer A + ( + 3 mM BME). Next, appropriate amount of TEV protease was added and the protein solution was incubated at 2 °C overnight to allow full cleavage of hTPX2-TEV-EGFP-His_8_. The solution was subjected to Ni-IMAC purification again where hTPX2 was eluted first by imidazole-free Buffer A + ( + 3 mM BME). Cleaved EGFP-His_8_ protein fragment and His-tagged TEV protease would bind more tightly to the Ni-column and hence could only be eluted using higher concentrations of imidazole. hTPX2 fractions were combined, concentrated via centrifugal filtration and subjected to size-exclusion chromatography using Superdex 200 10/300 increase GL column. hTPX2 in PBS was concentrated, snap-frozen in liquid nitrogen and stored at −80 °C before use. This hTPX2 product will be ready for camelid nanobody screening. For quantification of protein concentration, typically 1 μl of protein sample was loaded onto the *DS-11FX(* + *)* DeNovix Spectrophotometer/Fluorometer. A280 was read with M.W. and *ε* being accounted in order to precisely determine the protein concentration: ***c*** (mg·ml^−1^) = [**A280** · **M.W**.(g·mol^−1^)]/***ε***(L · mol^−1^ · cm^−1^).

#### Preparation of the hTPX2 nanobody for ITC measurement

pET28b(TEV)_His_8_-mCherry-TEV-TBP nanobody plasmid was first cloned and the protein was expressed following the above-mentioned protocol; bacteria culture was shaken at 30 °C overnight after IPTG induction. The purified nanobody chimera was treated with appropriate amount of TEV protease at 2 °C overnight to allow complete cleavage. The protein solution was subjected to Ni-IMAC purification again and the TPX2 nanobody was eluted by imidazole-free Buffer A to produce the TPX2 nanobody with sufficient purify for the subsequent analysis. Cleaved His_8_-mCherry fragment, His-tagged TEV protease and most impurities were removed due to their higher binding affinities to Ni-NTA column.

#### Preparation of Cys-TBP-CAAX with N-terminal cysteine via TEV protease cleavage

The precursor His_8_-TEV’-TBP-CAAX was expressed from *E. coli*. culture according to the above-mentioned general protocols. More specifically the respective Rosetta 2a *E. coli*. culture was allowed to grow at 30 °C overnight after adding IPTG. After cell pelleting, cell lysis, centrifugation, and gradient Ni-IMAC purification, His_8_-TEV’-TBP-CAAX was obtained as a solution in pH 8.0 Buffer A + ( + 3 mM BME). Some impurity bands were present according to SDS-PAGE analysis, which will be facilely removed in the next step. Next, appropriate amount of TEV protease was added and the protein solution was incubated at 2 °C overnight to allow full cleavage of His_8_-TEV’-TBP-CAAX at the TEV’ site (TEV’ sequence: ENLYFQ ↓ C). The protein solution was subjected to reverse Ni-IMAC purification again and the Cys-TBP-CAAX was eluted by imidazole-free Buffer A + ( + 3 mM BME) with sufficient purify. Cleaved His_8_-TEV’ peptide fragment, His-tagged TEV protease and most impurities that bind more tightly to Ni-NTA column will be removed.

### Generation of TBP from a naive library via M13 phage display

In general, the TBP was obtained using the above prepared hTPX2 as the antigen to screen a naïve phage library. pADL10b phagemid was used and panning and ELISA selection were performed in Alpa-Life (Shenzhen, P.R. China). The phage library consisting of ~2 × 10^9^ molecules was generated from bloods collected from 103 camelids. Around 1.4 mg of hTPX2 were expressed and purified with a qualified purity, stored at −80 °C as snap frozen aliquots, and thaw at 4 °C overnight just before use. 50 μg hTPX2 in 2 ml PBS or 50 μg BSA (control) in 2 ml PBS was used to coat Nunc immune tube (Cat# 444202, Thermo Scientific) overnight. Panning was performed according to the standard operation protocol (SOP) of the company, which produced a sub-phage library ready for ELISA selection. After three rounds of ELISA selection, the nanobody with the highest affinity was identified and was designated as TPX2 binding protein, i.e. TBP nanobody.

### General EPL protocol for the assembly of SNACIPs

Proteins to be ligated were expressed as a fusion chimera with a C-terminal Mxe GyrA intein tag by cloning the respective gene into pTXB1 vector. Afterwards, this fusion chimera was expressed, purified and buffer exchanged in Buffer A (pH 8.0 PBS, 0.5 M NaCl, 3% glycerol). Typically, the Mxe GyrA intein fusion protein was reconstituted to around 8.5 mg·ml^−1^ before ligation. To initialize the ligation reaction, 1/4 volume of pH 8.0 sodium 2-mercaptoethanesulfonate (MENSNa) stock solution (2 M) was added as the intein cleavage reagent and 1/4 volume of pH 8.0 4-mercaptophenylacetic acid (MPAA) stock solution (1.1 M) was added as the catalyst. Finally, N-terminal cysteine-functionalized fragment was added to the reaction solution at a final concentration of 0.5–1 mM. The reaction mixture was incubated on ice. SDS-PAGE was used to monitor the ligation process and most of the intein fusion will be converted to the ligation product after 2–4 days of incubation. The ligated product could be further purified via step-gradient (0→500 mM imidazole) gravity Ni-IMAC chromatography using high affinity Ni-charged Resin FF (Cat# L00666-25, GenScript). Sometimes, chitin resin (Cat# S6651L, NEB) may need to be used to remove CBD-fused non-ligated product if necessary.

### Stepwise protocol for assembly of cRGT

- His_6_-GBP-Intein-CBD buffer exchanged in Buffer A (pH 8.0) was added MENSNa (pH 8.0, 2 M stock) to final 0.4 M, MPAA (pH 8.0, 1.1 M stock) to final 0.2 M and CysTMP (25 mM stock) to final 1 mM; the reaction solution was incubated on ice for one day and additional 1 mM CysTMP was added the second day.
- Conversion will reach maximal after additional one or more days as monitored by SDS-PAGE, and the reaction solution was subjected to Ni-NTA IMAC purification to remove cleaved intein and some unreacted GBP-Intein-CBD.
- Incubate with chitin resin in a Buffer A pre-equilibrated chitin column and rotate for 2 h in cold room; collect the flowthrough and elute the column by Buffer A until A280 reaches zero; flowthrough and elution fractions were combined and concentrated to give the GBP-TMP conjugate.
- Buffer exchange (1-2×) into pH ~8.3 **DTNP Buffer** (50 mM Na_2_HPO_4_, 0.5 M NaCl) using the same centrifugal filtration tube used above; degas via brief sonification, add 2 equiv. TCEP (20 mM stock), flush with Ar, incubate for 45 min and degas via sonification again.
- Add 10 equiv. DTNP (100 mM in 0.5 M Na_2_HPO_4_) for 45-60 min to activate Cys and the reaction solution will soon turn yellowish; buffer exchange (2-3×) into pH 9.0 **Disulfidization Buffer** (50 mM HEPES, 0.5 M NaCl) to remove the excess of DTNP.
- Degas via brief sonification, add 25 mM Cys-cR10* to final 0.5-2 mM, incubate on ice for 30 min; additional Cys-cR10* may be added to increase conversion; buffer exchange (1-2×) into pH 7.2 PBS and the cRGT product in PBS was aliquoted, snap frozen in liquid nitrogen and stored under −80 °C before use.

### Assembly of cRTC via one-pot tandem bioorthogonal ligation

In a typical reaction, Cys-TBP-CAAX in pH 7.2 PBS (1.78 mg·ml^−1^, 80 μl) was added 4 μl ACBT (~10 mM stock, final ~0.5 mM) and incubated at 2 °C overnight to complete the ligand as confirmed by SDS-PAGE. The next day morning, the reaction solution was buffer exchanged to remove the excess of ACBT to produce ACBT-TBP-CAAX in Buffer A + (1.95 mg·ml^−1^, 71 μl). In parallel, 15 μl Cys-cR10* (25 mM stock in DMSO, 0.375 μmol) and 10 μl of BCN-PEG_2_-maleimide (25 mM stock in DMSO, 0.25 μmol) were mixed in 80 μl PBS at RT and incubated for ~1 h to complete thiol-maleimide ligation. 3.9 μl in situ generated cR10*-BCN (~2.4 mM, ~1.2 equiv.) were added into ACBT-TBP-CAAX solution and incubated for a few hours to complete the click reaction. After buffer exchange, cR10*-TBP-CAAX (cRTC) was obtained as a solution in PBS (1.52 mg·ml^−1^, 73 μl) in an overall yield of 78%.

### Image analysis

Microscopic images were analyzed and processed with ImageJ/Fiji and prepared for presentation using Microsoft Office PowerPoint. Image manipulations were restricted to adjustment of brightness level (i.e. linear stretch), background subtraction, cropping, rotating, scaling, and false color-coding using Look-Up Tables (LUT). Pearson’s correlation coefficient (PCC) was employed for colocalization analysis using the “Manders_Coefficients.class” plugin for ImageJ/Fiji. This plugin is provided with this paper as Supplementary Software. Usually, there is only a single cell within an imaging field; otherwise, a single cell was first selected using the polygon selections tool and then remove extra cells (Edit/Clear outside) prior to PCC analysis. The images were converted to 8-bit depth (Image/Type/8-bit) prior to analysis, and typically no less than ten cells were analyzed for each PCC analysis. For measuring translocation ratio, the mitochondria area was first identified by performing auto-threshold of the EGFP channel in ImageJ/Fiji (Image/Adjust/Threshold/Auto) and the identified mitochondria regions were selected using Wand tool and saved (Analyze/Tools/ROI manager/Add). Then the average mCherry fluorescence intensity at mitochondria area (*I*_mito_) and the area size (*S*_mito_) were measured (Analyze/Measure). The whole cell area (*S*_cell_) and average mCherry intensity (*I*_cell_) were also measured for each cell. Hence, the average cytosol area (*S*_cytosol_ = *S*_cell_ – *S*_mito_) and average cytosol intensity [*I*_cytosol_ = (*I*_cell_×*S*_cell_ – *I*_mito_×S_mito_)/S_cytosol_] were calculated. Finally, the mitochondria/cytosol fluorescence intensity ratio was obtained (ratio = *I*_mito_/*I*_cytosol_).

PM targeting index which reveals the degree of PM localization was determined through line-scan analysis. Lines were drawn to (1) avoid nucleus, (2) maximize the two membrane signals on both sides of the cell, and (3) just cross the two boundaries of the entire cell. Three line-scan analysis from three different directions were performed for each cell and their calculated index values will be averaged. The cytosolic signal ***I***(c) was taken as the average signal intensity across the middle 1/2 region of the line scan analysis. And the plasma membrane signal ***I***(p) was taken as the highest signal along the line-scan analysis. Hence, the PM targeting index that indicates the ratio between PM signal to cytosol signal is calculated using Eq. (1) below:

1 $${{{{{\bf{P}}}}}}{{{{{\bf{M}}}}}}\,{{{{{\bf{t}}}}}}{{{{{\bf{a}}}}}}{{{{{\bf{r}}}}}}{{{{{\bf{g}}}}}}{{{{{\bf{e}}}}}}{{{{{\bf{t}}}}}}{{{{{\bf{i}}}}}}{{{{{\bf{n}}}}}}{{{{{\bf{g}}}}}}\,{{{{{\bf{i}}}}}}{{{{{\bf{n}}}}}}{{{{{\bf{d}}}}}}{{{{{\bf{e}}}}}}{{{{{\bf{x}}}}}}=[I(p)/I(c)+I\hbox{'}(p)/I\hbox{'}(c)+I\hbox{''}(p)/I\hbox{''}(c)]/3$$

### Statistics and reproducibility

All microscopic imaging experiments were representative of at least three independent repeats if not otherwise stated; EdU experiments were performed twice with similar results and images from only one imaging section was used for analysis to keep fluorescence intensity readout consistent. Representative SDS-PAGE images were from at least three independent repeats with similar results; representative confocal microscopic images were from at least ten independent cells with similar results; IP experiment was performed once which already provided clear results. No randomization nor blinding was used in this study. Origin and Microsoft Excel were used for plotting, data fitting, graphing and statistical analysis. All box plots show mean (square), median (bisecting line), bounds of box (75^th^ to 25^th^ percentiles), outlier range with 1.5 coefficient (whiskers), and minimum and maximum data points (lower/ upper whiskers). *t*_1/2_ of dimerization rate was determined by a one-phase exponential decay non-linear regression with the ordinary Least Squares fit. Student’s *t*-tests were used to compare two experimental conditions. Unless otherwise specified, one-sided unpaired *t*-tests were performed, as for example cells with or without drug/inducer treatment. When necessary, stars were used to denote *P*-values for indicated statistical tests (*P < 0.05; **P < 0.01; ***P < 0.001; ****P < 0.0001). Exact *P*-values were indicated for critical experiments.

### Reporting summary

Further information on research design is available in the Nature Portfolio Reporting Summary linked to this article.

## Supplementary information

Supplementary Information

Description of Additional Supplementary Files

Supplementary Data 1

Supplementary Software

Reporting Summary

## Data Availability

The data that support the findings of this study are available within the Article, Supplementary Information. Source data are provided with this paper.

## Source data

Source Data

## Code availability

“Manders_Coefficients.class” plugin and how to use it in ImageJ/Fiji for the calculation of PCC values are provided with this paper.
