# Supplementary material for: Small molecule-nanobody conjugate induced proximity controls intracellular processes and modulates endogenous unligandable targets
Source: Nat Commun. 2023 Mar 24;14:1635. doi: 10.1038/s41467-023-37237-x (PMC10039045; doi:10.1038/s41467-023-37237-x)
Supplement: Supplementary file 1 — Supplementary Information [file 41467_2023_37237_MOESM1_ESM.pdf]

Small molecule-nanobody conjugate induced proximity controls intracellular processes and modulates endogenous unligandable targets

Xiaofeng Sun[1,2,3], Chengjian Zhou[1,2,3], Simin Xia[1,3], Xi Chen[1,2,4]*

[1]Laboratory of Chemical Biology & Frontier Biotechnologies, The HIT Center for Life Sciences (HCLS), Harbin Institute of Technology (HIT), Harbin, P.R. China, 150001

[2]School of Life Science and Technology, HIT, Harbin, P.R. China, 150001

[3]These authors contributed equally

[4]Lead contact

*Correspondence: chenxihit@hit.edu.cn

SUPPLEMENTARY INFORMATION

LIST OF ABBREVIATIONS

Key abbreviations of nanobodies, SNACIPs, protein tags and others in this paper:

GBP: green fluorescent protein binding protein
RBP: mCherry red fluorescent protein binding protein
TBP: TPX2 binding protein
TBP-TBP: tandem bivalent TPX2 nanobody
cRGT: cR10*-SS-GBP-TMP
cRRT: cR10*-SS-RBP-TMP
cRTC: cR10*-TBP-CAAX
CTTC: mCherry-CPP-TBP-TBP-CAAX
CTT: mCherry-CPP-TBP-TBP
CPP: cell-penetrating peptide
eDHFR or **ED**: *E. coli* dihydrofolate reductase
HT: HaloTag
TMP: trimethoprim
EPL: expressed protein ligation

SUPPLEMENTARY FIGURES

a **HPLC Chromatograph** (C18, λ=220 nm, 1 ml·min^{-1})

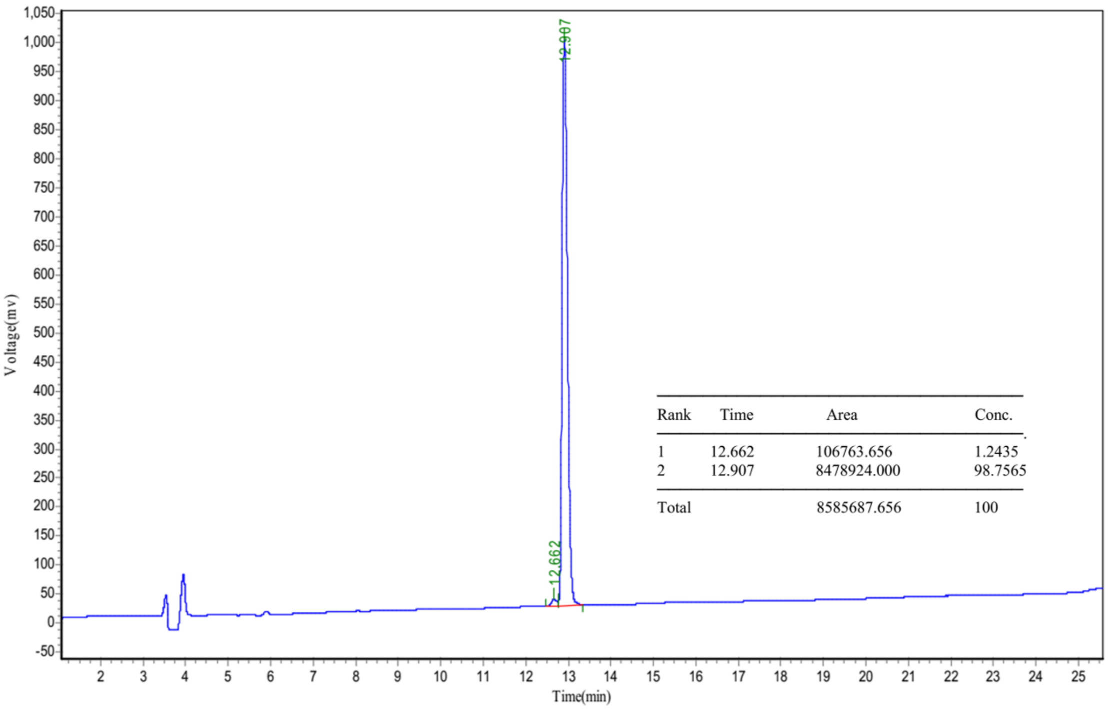

b **MS Spectrum:** m/z 736.4 [M+3H]$^{3+}$

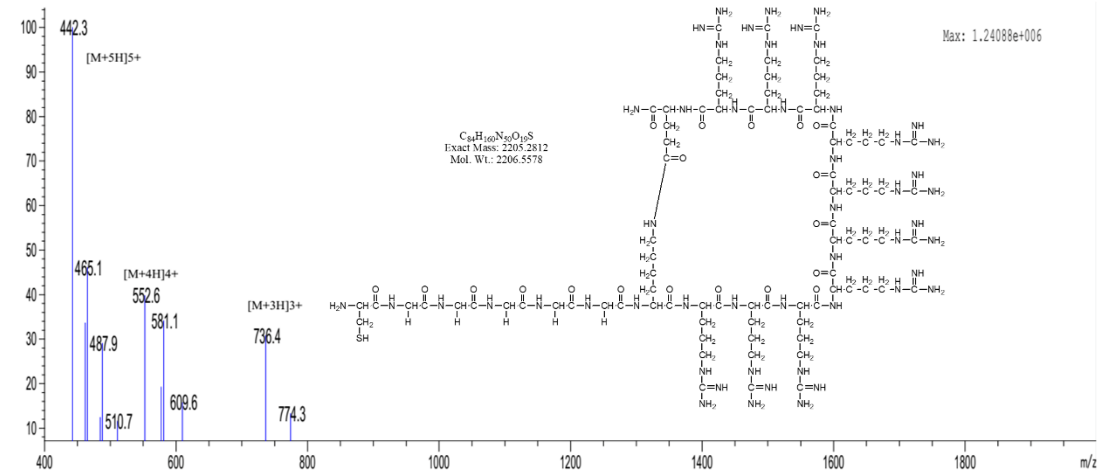

Supplementary Figure 1| HPLC and MS characterizations of the cyclic peptide Cys-cR10*. a, HPLC chromatograph revealed a high purity of 98.8%. **b**, Mass spectrometry (MS) spectrum confirmed the correct M.W. of Cys-cR10*.

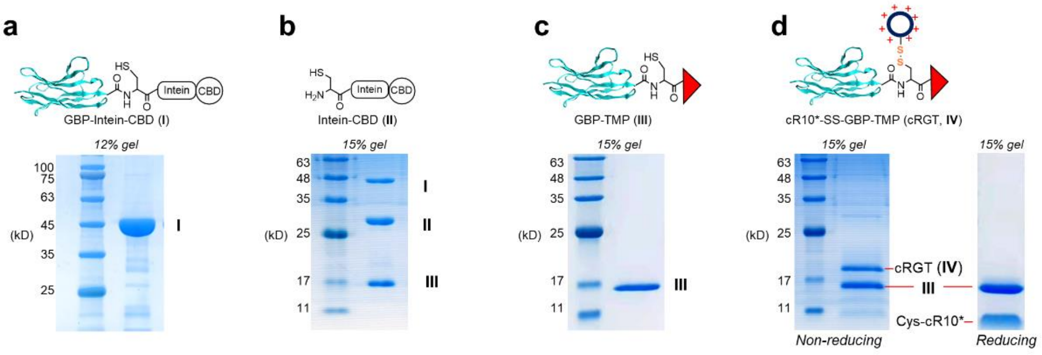

Supplementary Figure 2| SDS-PAGE characterizations of cR10*-SS-GBP-TMP (cRGT, IV) and its intermediates (I, III). **a**, SDS-PAGE of GBP-Intein-CBD (**I**). **b**, SDS-PAGE of the EPL reaction showing ~80% conversion. **c**, SDS-PAGE of GBP-TMP (**III**). **d**, Non-reducing SDS (left) revealed the formation of cRGT (**IV**) with around half conversion while reducing SDS-PAGE (10 min at room temperature, right) showed that cR10* can be reductively cleaved from cRGT.

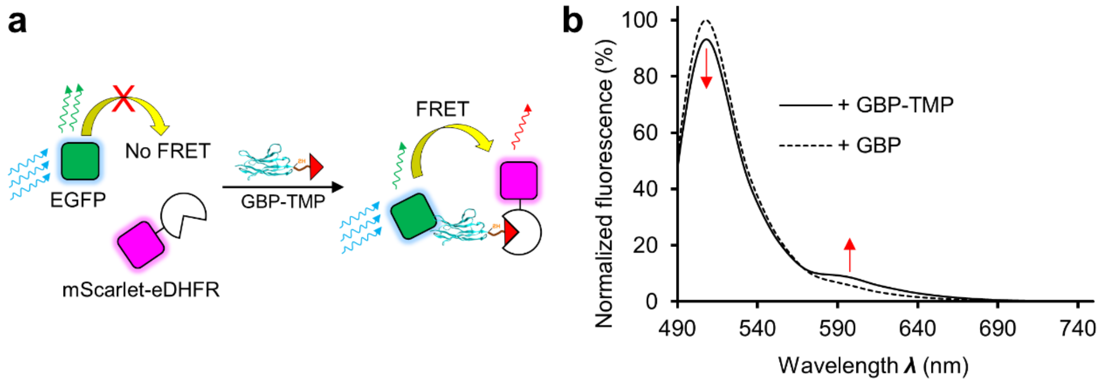

Supplementary Figure 3| GBP-TMP induced the dimerization between eDHFR and EGFP revealed by spectral Förster resonance energy transfer (FRET). a, Schematic view of the FRET assay. **b**, Spectral FRET results revealed the Förster resonance energy transfer from EGFP donor to mScarlet-eDHFR acceptor in the presence of GBP-TMP dimerizer but not GBP. A bit excess (1.1 equiv.) of GBP-TMP or GBP was added into the mixture of 5 μM EGFP and 5 μM mScarlet-eDHFR in PBS (pH 7.4) supplemented with 1 mM TCEP, 3 % glycerol, and 0.5 M NaCl; the fluorescence spectra were recorded using a fluorometer with excitation wavelength set at 470 nm.

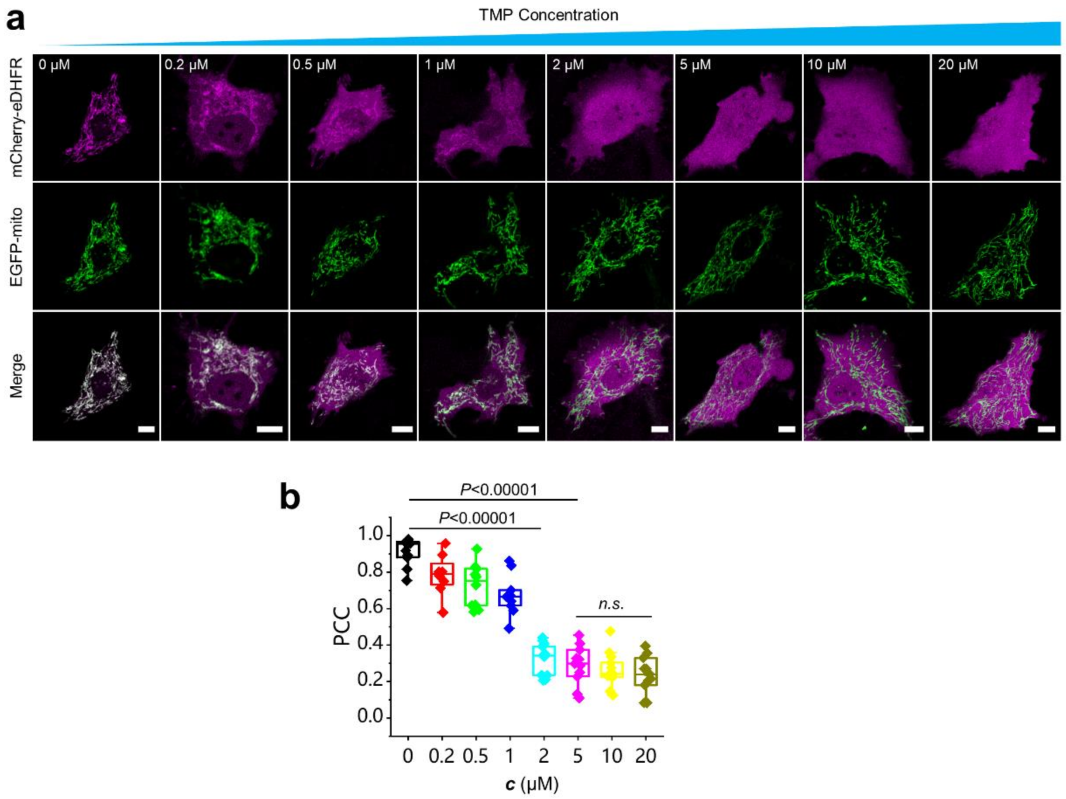

Supplementary Figure 4| The dimerization between EGFP and eDHFR induced by cRGT was reversed TMP in a dose-dependent manner. a, Live HeLa cells coexpressing mCherry-eDHFR (red, cytosolic) and EGFP-mito (green, mitochondria) were treated with cRGT inducer (24 μM, 1.5 h) to induce the dimerization between mCherry-eDHFR and EGFP; afterwards, gradient concentrations of TMP (0, 0.2, 0.5, 1, 2, 5, 10, and 20 μM) in imaging medium was used to replace the cRGT-containing imaging medium for 10 min. Confocal microscopic images revealed dose-dependent reversing of the dimerization by TMP. **b**, Statistical PCC colocalization analysis between mCherry and EGFP (n=11/8/10/10/13/12/13/11 cells for 0/0.2/0.5/1/2/5/10/20 μM); one-sided Student's *t*-test was used; see Methods for description of box plots. All scale bars: 10 μm.

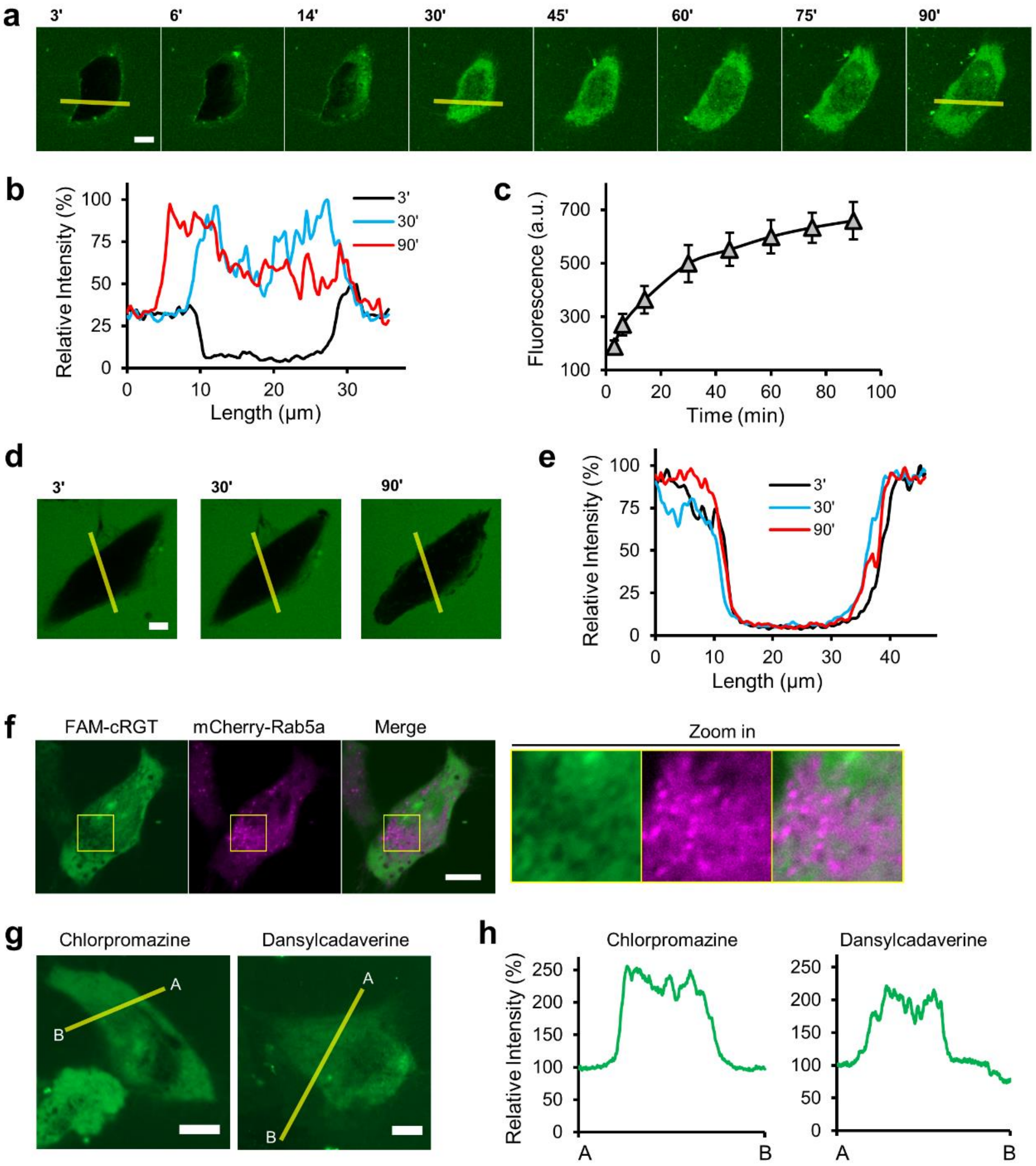

Supplementary Figure 5| Visualization of non-endocytic entry of cRGT into live HeLa cells via transduction. **a**, Fluorescein (5-FAM-NHS, 1 equiv., 4 °C, overnight) labeled cRGT (24 μM) in phenol-red free imaging medium was used to replace the full DMEM, and then HeLa cells were imaged using confocal fluorescence microscopy. cRGT gradually entered into the live HeLa cell and diffusively distributed inside the cell with immediate bioavailability without any endosomal puncta formation; this phenomenon was known as transduction which suggested that the entry occurred in a non-endocytic fashion[1, 2]. **b**, Line profile analysis (from left to right). **c**, Quantification of the fluorescence intensity enhancement along time (n=14 cells); bar graphs denote mean ± standard error of the mean (SEM). **d**, As a control experiment, fluorescein (5-FAM-NHS, 1 equiv., 4 °C, overnight) labeled glycine (24 μM) did not enter live HeLa cells at all during the course of this experiment. **e**, Line profile analysis (from upper left to lower right). **f**, FAM-cRGT entered live HeLa cells (24 μM, 1 h) and smoothly

distributed in the cytosol without colocalization with endosomal puncta (labeled by mCherry-Rab5a). **f**, FAM-cRGT entered live HeLa cells (24 μM, 1 h) and smoothly distributed in the cytosol even in the presence of an endocytic inhibitor using either chlorpromazine (20 μM) or dansylcadaverine (50 μM). All scale bars: 10 μm.

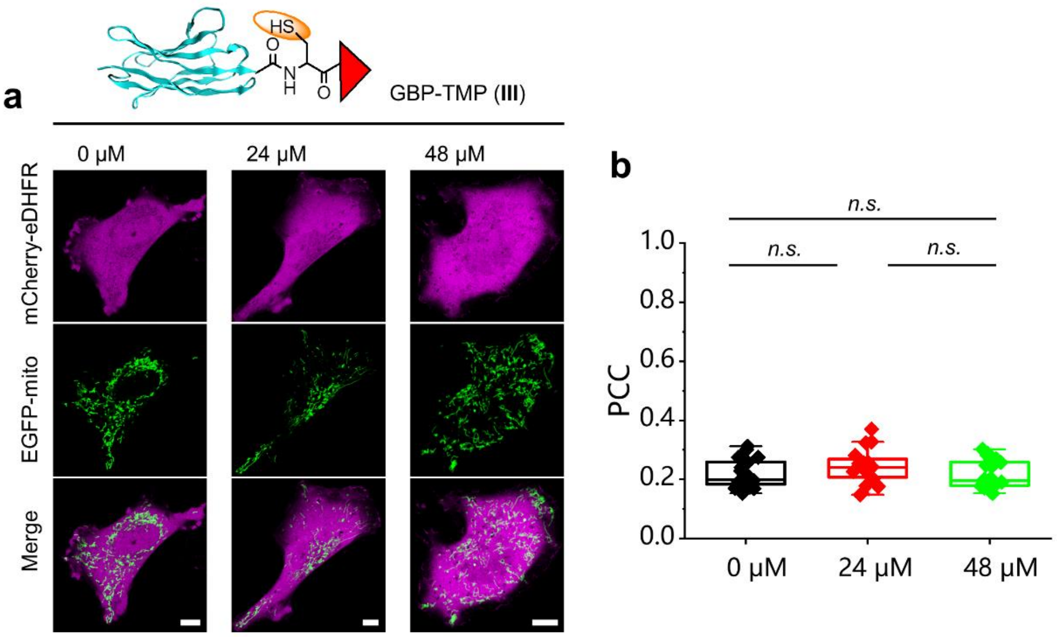

Supplementary Figure 6| GBP-TMP that is absent of cR10* cannot induce intracellular dimerization. a, Confocal microscopic images of live HeLa Cells coexpression of mCherry-eDHFR and EGFP-mito without adding GBP-TMP, after adding GBP-TMP (24 μM, 1.5 h) and after adding higher concentrations of GBP-TMP (48 μM, 1.5 h) show no positioning of mCherry-eDHFR to EGFP-mito on mitochondria. **b**, Statistical PCC colocalization analysis (n=18, 16, and 20 cells for 0 μM, 24 μM, and 48 μM, respectively); one-sided Student's *t*-test was used; see Methods for description of box plots. These results further highlight the necessity of cR10* module for intracellular delivery of cRGT and that the presence of GBP-TMP portion in cRGT product does not affect the function of cRGT in regulating intracellular processes.

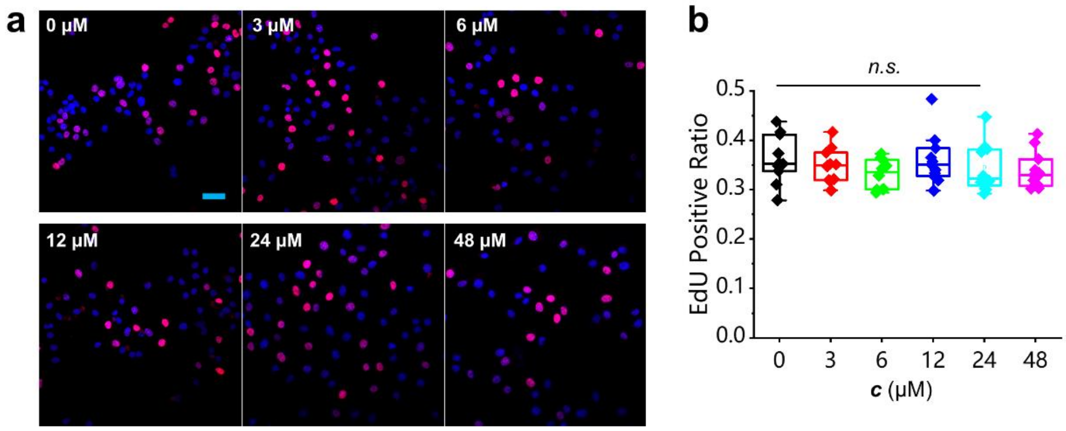

Supplementary Figure 7| cRGT does not show apparent cytotoxicity under the conditions used in this study according to EdU-based cytotoxicity assay. a, EdU-based cytotoxicity assay of live HeLa cells treated with the given concentrations of cRGT for 1.5 h and the cells were subjected to EdU assay; nucleus was stained by Hoechst (2.5 μg·ml^{-1}, blue) and S-phase cells were labeled with Apollo 567 dye (red). **b**, Quantification of the EdU positive ratio, which shows that no significant inhibition of cell proliferation was observed (n=10/10/10/11/12/11 cells for 0/3/6/12/24/48 μM); one-sided Student's *t*-test was used; around 10 fields were collected and analyzed for each concentration; see Methods for description of box plots. Scale bar: 50 μm.

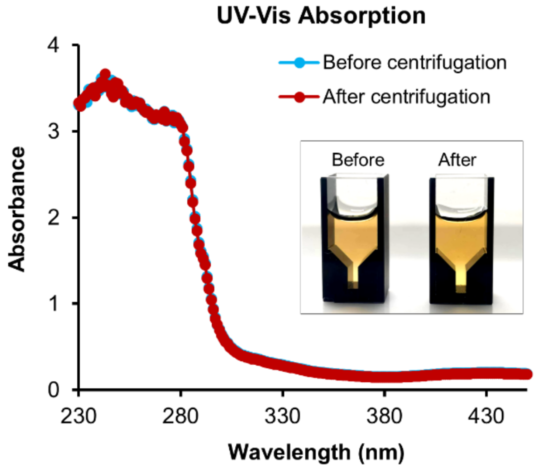

Supplementary Figure 8| UV-Vis absorption spectra of rapamycin solution in imaging medium before and after centrifugation suggest that no rapamycin precipitation occurs. The UV-Vis absorption spectra of 250 nM rapamycin in imaging medium before and after centrifugation show not detectable changes, suggesting that no precipitation of rapamycin occurs; also, both the solutions are fully transparent before and after centrifugation. This result is further supported by previous reports which showed that solubility of rapamycin in water is 2.6 µg/µl (i.e. 2.8 µM)[3], and that rapamycin precipitates only at micromolar concentrations of over 5 µM[4].

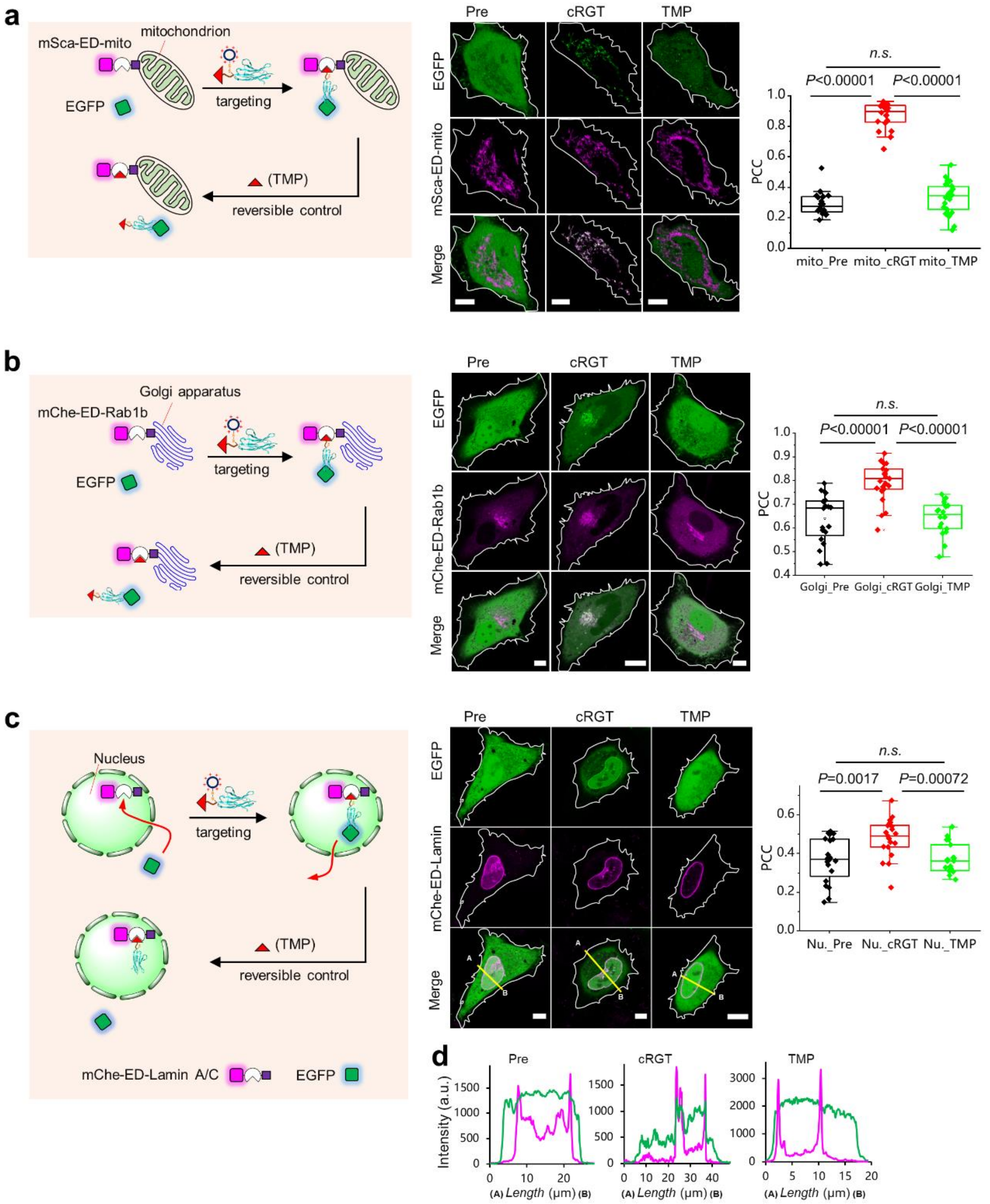

Supplementary Figure 9| cRGT controls the positioning of EGFP to different subcellular structures in live HeLa cells. a, cRGT (24 μM, 1.5 h) targets EGFP from cytosol to mScarlet-eDHFR-mito at mitochondria; positioning was rapidly reversed by applying TMP (10 μM, 10 min) (n=18, 20, and 22 cells for mito_Pre, mito_cRGT, and mito_TMP); one-sided Student's *t*-test was used; see Methods for description of box plots. **b**, Representative confocal microscopic images of live HeLa cells co-expressing mCherry-eDHFR-Rab1b (red, mainly Golgi localization) and EGFP (green) before adding cRGT (Pre), after adding cRGT (24 μM, 1.5 h) and after adding TMP (10 μM, 10 min) (n=20, 23, and 18 cells for Golgi_Pre, Golgi_cRGT, and Golgi_TMP); one-sided Student's *t*-test was used; see Methods for description of box plots. **c**,

Representative confocal microscopic images of live HeLa cells co-expressing mCherry-eDHFR-Lamin A/C (inner nucleus membrane marker) and EGFP before adding cRGT (Pre), after adding cRGT (24 μM, 1.5h), and after adding TMP (10 μM, 10 min) (n=20, 19, and 18 cells for Nu._Pre, Nu._cRGT, and Nu._TMP); one-sided Student's *t*-test was used; see Methods for description of box plots. **d**, Line profile analysis. Abbreviations in the figures: mScarlet, mSca; mCherry, mChe or R; EGFP, G; eDHFR, ED. One-sided Student's *t*-test was used in these experiments; all scale bars: 10 μm.

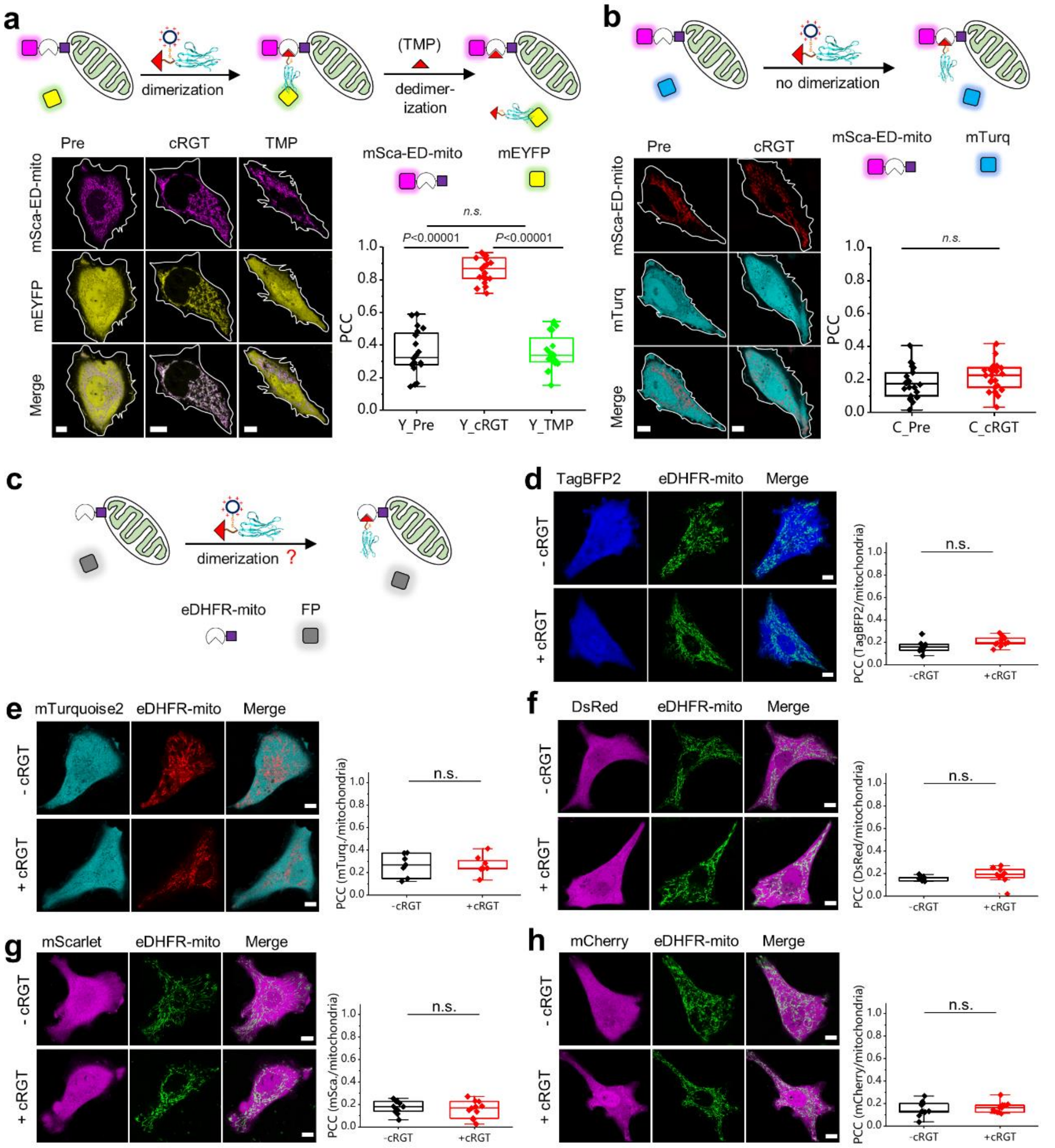

Supplementary Figure 10| Evaluation of the orthogonality of cRGT against alternative GFP variants and other FPs. a, cRGT (24 μM, 1.5 h) targets the yellow color GFP variant mEYFP from cytosol to mScarlet-eDHFR-mito at mitochondria; dimerization was rapidly reversed by applying TMP (10 μM, 10min) (n=20, 19, and 20 cells for Y_Pre, Y_cRGT, and Y_TMP); one-sided Student's *t*-test was used; see Methods for description of box plots. **b**, cRGT (24 μM, 1.5 h) fails to control the cyan color GFP variant mTurquoise2, suggesting that cRGT is orthogonal to mTurquoise2. Lower right panels in both **a**) and **b**): statistical PCC analysis between the two channels (n=22 cells for C_Pre, n=23 cells for C_cRGT); one-sided Student's *t*-test was used; see Methods for description of box plots. **c**, Validation of the orthogonality of cRGT to other commonly used fluorescent proteins (FPs) using a none FP-fused eDHFR-mito construct in order to exclude any possibility originated from FP-FP interactions. **d-h**, Live HeLa cells co-expressing TagBFP2, mTurquoise2, DsRed, mScarlet or mCherry

and eDHFR-mito were or were not treated with cRGT (24 μM, 1.5 h). Confocal microscopic images together with statistical PCC analysis suggested that no dimerization was induced in all sets of experiments (n=8 and 10 cells for -cRGT and +cRGT in **d**; n=7 and 8 cells for -cRGT and +cRGT in **e**; n=9 and 9 cells for -cRGT and +cRGT in **f**; n=11 and 11 cells for -cRGT and +cRGT in **g**; n=10 and 11 cells for -cRGT and +cRGT in **h**); one-sided Student's *t*-tests was used; see Methods for description of box plots. All experiments used mito-tracker green to stain the mitochondria aside from **e**) which used mito-tracker red to stain mitochondria. Abbreviations in the figures: mScarlet, mSca; eDHFR, ED; mEYFP, Y; mTurquoise2, mTurq or C. One-sided Student's *t*-test was used in these experiments; all scale bars: 10 μm.

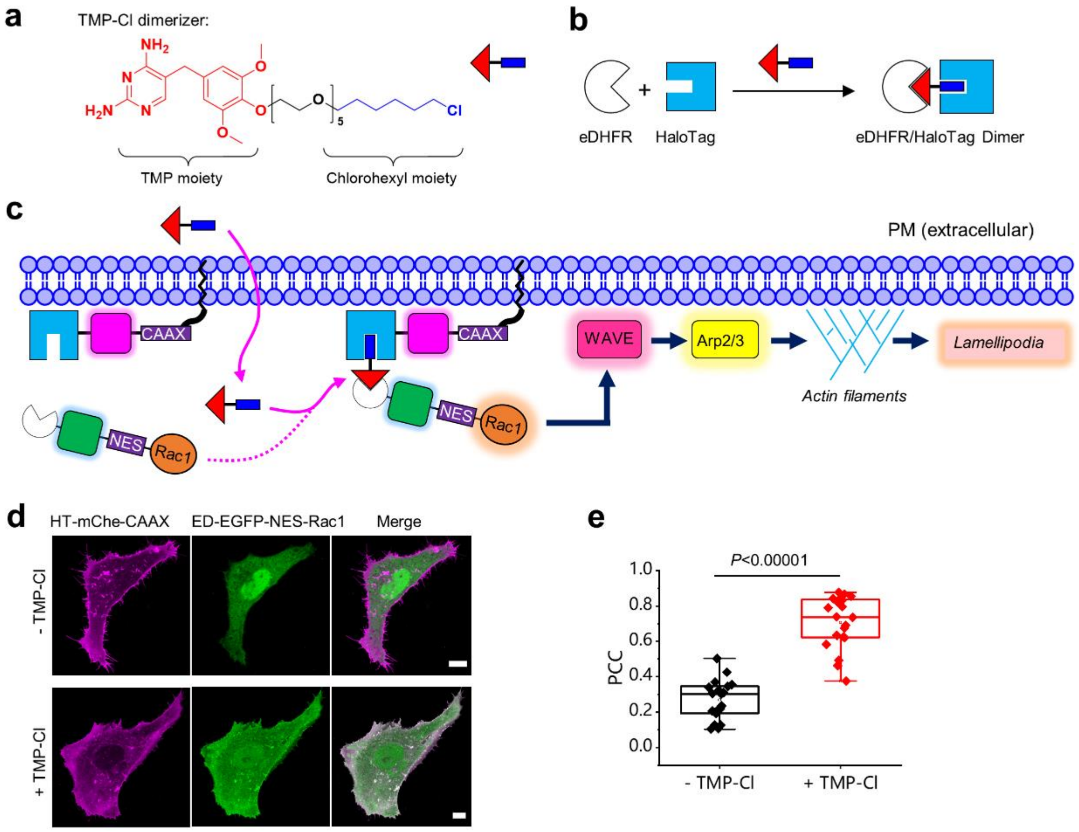

Supplementary Figure 11| TMP-Cl induces the dimerization in a less complete fashion. a, Structural elements of TMP-Cl which features a TMP moiety for binding with eDHFR and a chlorohexyl moiety for covalently binding with HaloTag. **b**, TMP-Cl induces the dimerization between eDHFR and HaloTag. **c,** Schematic view of TMP-Cl induced targeting of Rac1 to PM for activation of the signaling cascade of lamellipodia formation. WAVE: WASp-family verprolin-homologous protein; Arp2/3: actin-related proteins-2/3. **d**, Representative confocal microscopic images of live HeLa cells co-expressing HT-mChe-CAAX and ED-EGFP-NES-Rac1 (HT: HaloTag; ED: eDHFR; Rac1: Rac1Q61LΔCAAX) treated or without treated by TMP-Cl (10 μM, 1h) followed by washing out the excess of TMP-Cl (30 min). **e**, Statistical PCC analysis revealed a moderate colocalization degree with PCC around 0.7 (n=19 cells for -TMP-Cl and n=20 cells for +TMP-Cl); one-sided Student's *t*-test was used; see Methods for description of box plots. Scale bars: 10 μm.

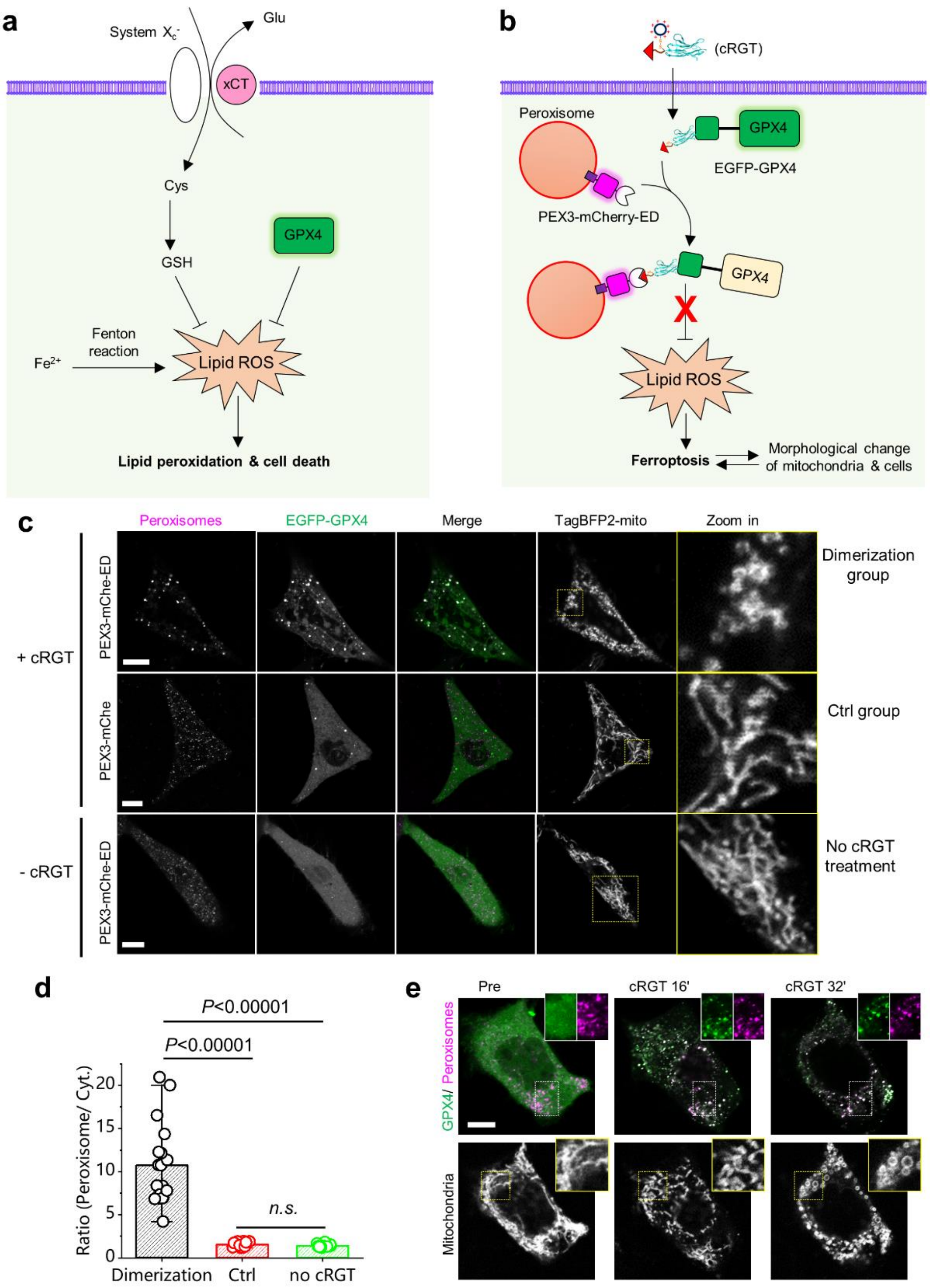

Supplementary Figure 12| cRGT activates ferroptosis in cancer cells by bringing GPX4 to the proximity of peroxisomes. **a**, A brief schematic view of the ferroptosis pathway that shows the essential role of GPX4 in protecting cells from ferroptosis. **b**, Schematic view of how cRGT brings EGFP-GPX4 to PEX3-mCherry-ED on the surface of peroxisomes to promote ferroptosis in cancer cells through deactivation of GPX4. GPX4 is recruited to non-functional peroxisomal location and that its catalytic selenocysteine residue is susceptible to oxidative deactivation that cause the loss of its suppression of ferroptosis. **c**, Live HeLa cells were transfected of PEX3-mCherry-

eDHFR (dimerization group and no cRGT group) or PEX3-mCherry (control group), EGFP-GPX4 and TagBFP2-mito (mitochondria marker). 22 h post-transfection, cells in the dimerization and control groups were treated with cRGT (24 μM, 2 h). According to confocal microscopic images, only the dimerization group showed repositioning of EGFP-GPX4 to peroxisomes along with the characteristic features of ferroptotic cells. **d**, Statistic quantification of the average EGFP fluorescence ratio between peroxisomes region and cytosol region; one-sided Student's *t*-test was used (dimerization, n=18 cells; Ctrl, n=15 cells; no cRGT, n=19 cells); bar graphs denote mean ± standard deviation (SD). **e**, A live HeLa cell was captured before adding cRGT (Pre), 16 min after adding cRGT (16'), and 32 min after adding cRGT (32'), which clearly showed the recruitment of GPX4 to peroxisomes and prominent morphological change of mitochondria along time. Scale bars: 10 μm.

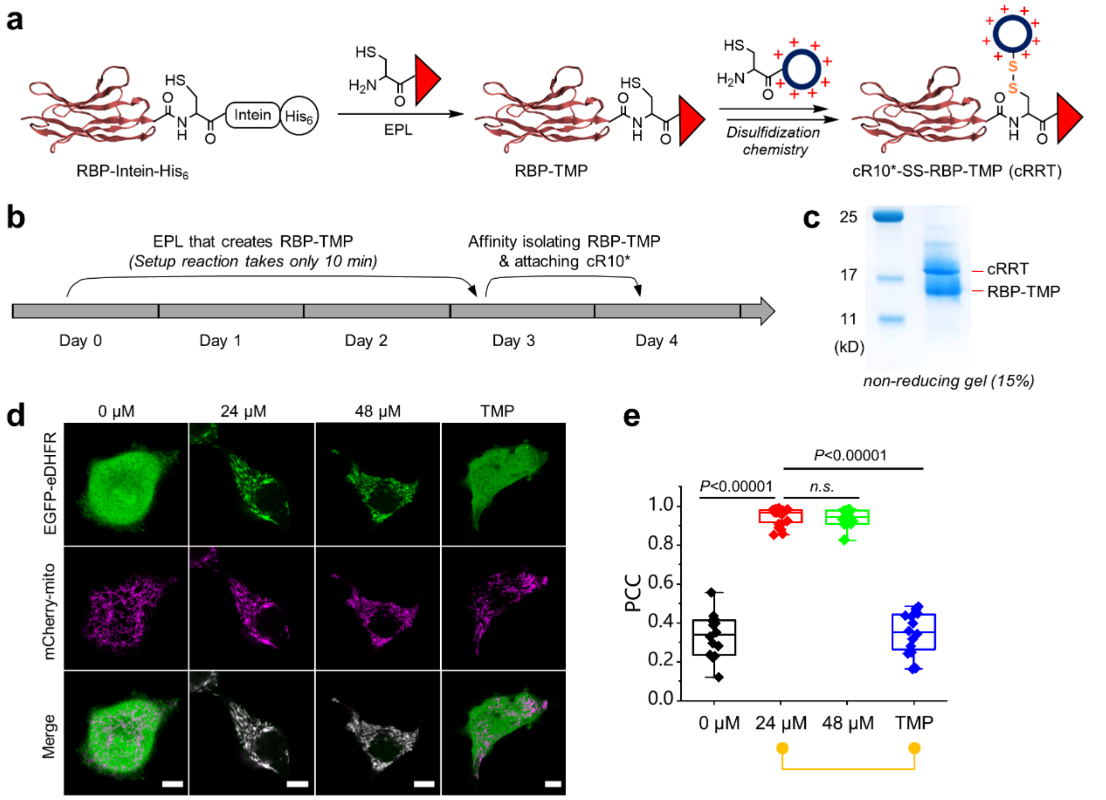

Supplementary Figure 13| Design and facile assembly of a new SNACIP inducer, cR10*-SS-RBP-TMP (cRRT), for control of the protein-protein proximity. a, Schematic view of the assembly of cRRT via expressed protein ligation (EPL) and disulfidization chemistry similar to the preparation of cRGT. **b**, Schematic view of the time flow reveals that cRRT can be facilely assembled. Note that EPL requires less than 10 min to setup; hence in reality the assembly requires only two days of laboratory works. **c**, SDS-PAGE characterization of cRRT, which reveals ~60% portion of the cR10* conjugated cRRT. **d**, Live HeLa cells coexpressing mCherry-mito and EGFP-eDHFR treated with cRRT (24 μM or 48 μM, 1.5 h) shows a high-degree of dimerization close to 1.0; TMP (10 μM, 10 min) completely abolished the dimerization that was induced by cRRT (24 μM). **e**, Statistical PCC colocalization analysis (n=14 cells for 0 μM, n=24 cells for 24 μM, n=16 cells for 48 μM, n=16 cells for TMP); one-sided Student's *t*-test was used; see Methods for description of box plots. All scale bars: 10 μm

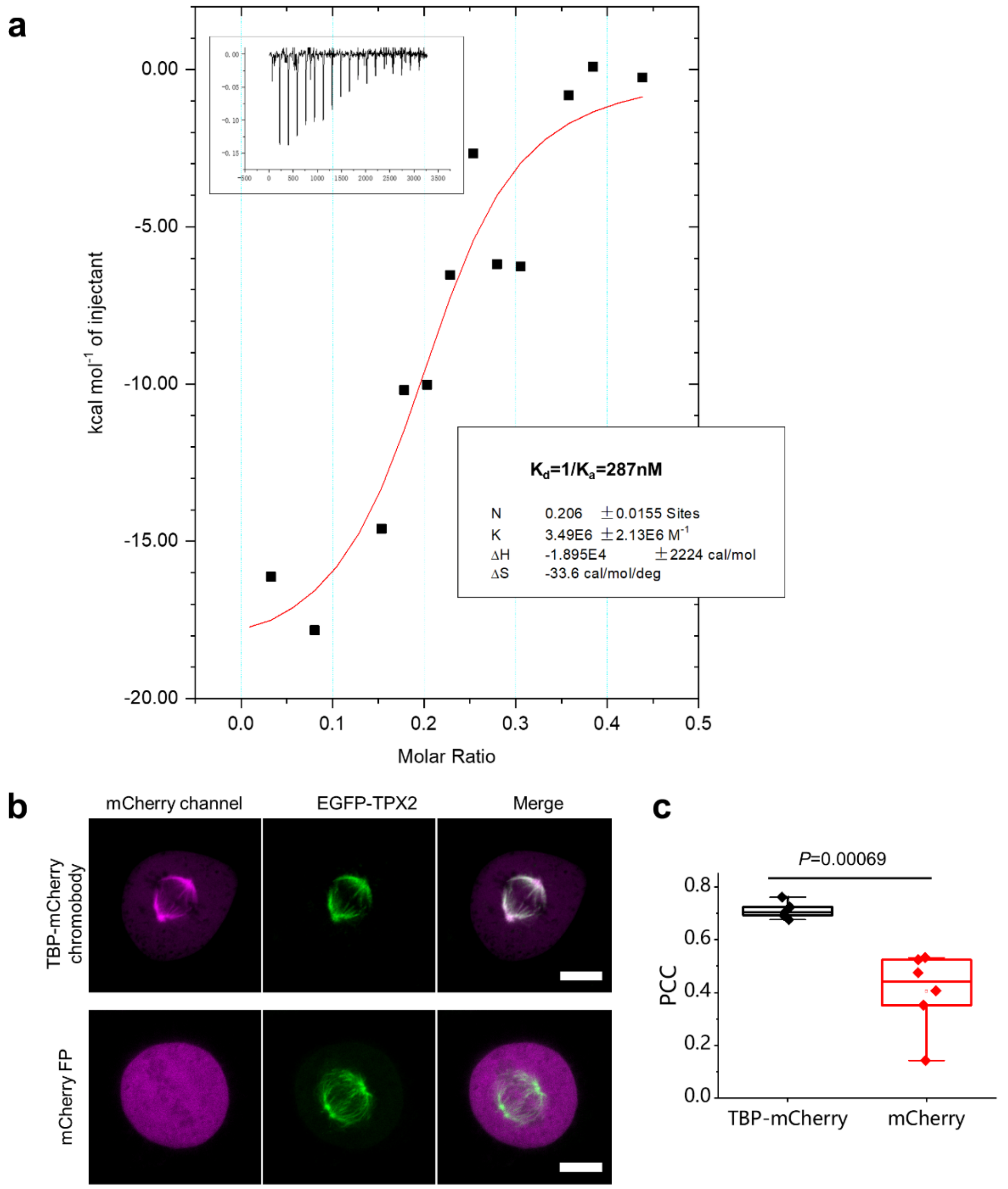

Supplementary Figure 14| Characterization TPB using isothermal titration calorimetry (ITC) and chromobody-based live-cell immunofluorescence staining. **a**, Characterization of the binding between TBP nanobody and hTPX2 antigen using ITC. Negative titration raw data peaks suggest that the binding is an exothermic reaction with a binding enthalpy ΔH being negative. According to the Wiseman plot, $K_d=1/K_a=287$ nM was derived at a stoichiometry of 1:5 for the binding between TBP and hTPX2. Note that the ΔS value is minus which indicates that the binding involves substantial conformational changes, matching well with the intrinsically disordered nature of hTPX2. **b**, Visualization of endogenous TPX2 using TBP-mCherry chromobody in live HeLa cells to show that TBP nanobody specifically binds with TPX2. A chromobody is small intracellular functional nanobody which consists of a V_{HH} genetically fused to a fluorescent protein. Chromobodies visualize endogenous cellular structures and processes in live cells and real-time. As a cell cycle protein, TPX2

localizes at spindles in M-phase, which allows clear visualization of its intracellular position. In this experiment, TBP-mCherry chromobody clearly colocalizes with TPX2 (visualized by co-expression of EGFP-TPX2) on spindle in M-phase, suggesting that TBP nanobody interacts with TPX2 highly specifically. **c**, PCC colocalization analysis between TPX2 and TBP-mCherry or mCherry (control); one-sided Student's *t*-test was used (TBP-mCherry, n=5 cells; mCherry, n=6 cells); see Methods for description of box plots. Scale bars: 10 μm.

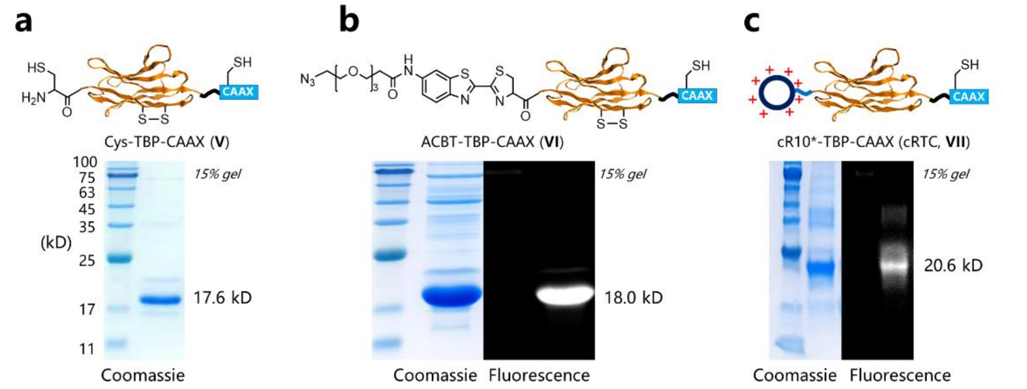

Supplementary Figure 15| Characterization of cRTC and its intermediates. a-c, SDS-PAGE and in-gel fluorescence analysis of the target molecule cRTC (**VII**) and its intermediates (**V**, **VI**).

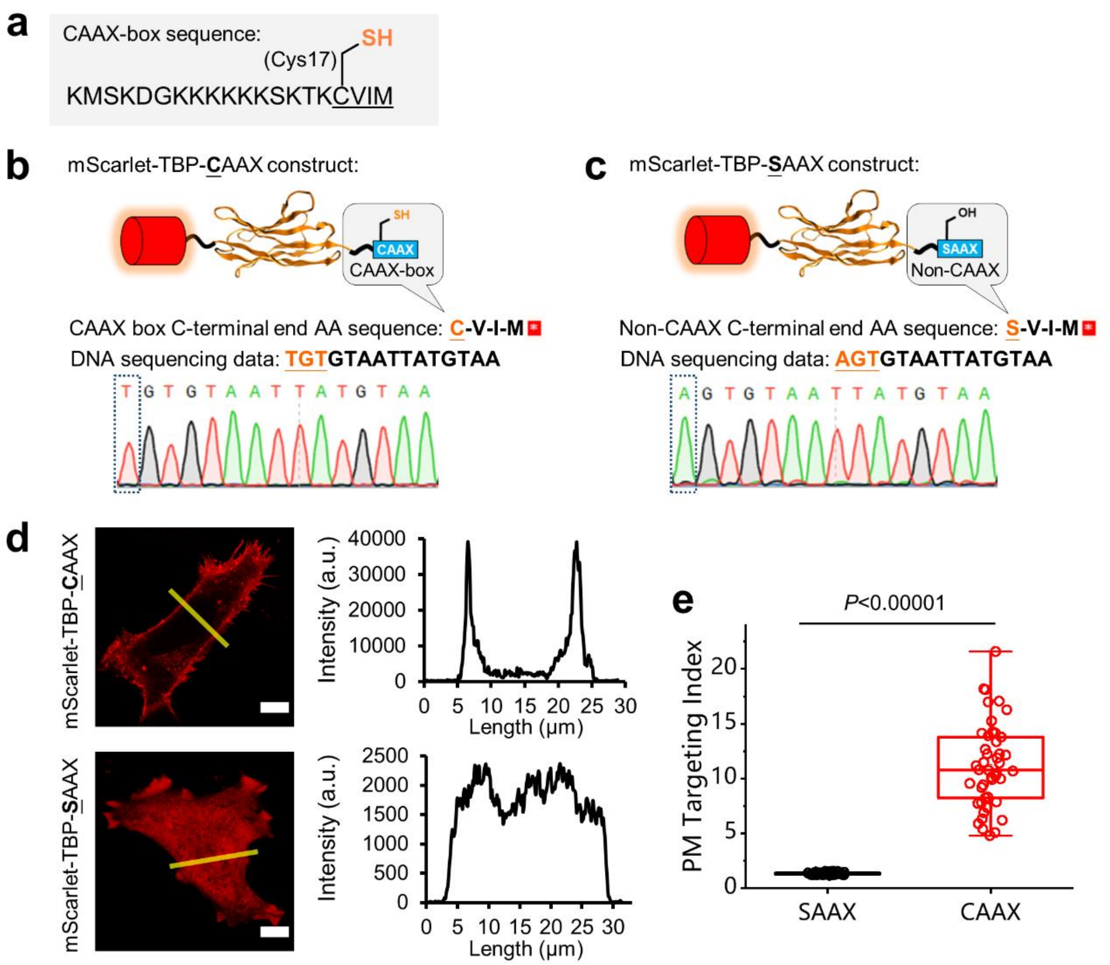

Supplementary Figure 16| The Cys17 residue of CAAX-box in TBP-CAAX chimera is responsible for post-translational prenylation. **a**, The amino acid (AA) sequence information of CAAX-box. **b**, The AA sequence and DNA sequencing data of the last four C-terminal residues of mScarlet-TBP-CAAX construct. **c**, The AA sequence and DNA sequencing data of the last four C-terminal residues in mScarlet-TBP-SAAX construct; it shows that the Cys17 residue responsible for prenylation has been mutated to Ser17 that is unable to undergo prenylation. **d**, According to the representative confocal microscopic images and corresponding line profile analysis (from left to right), mScarlet-TBP-CAAX shows a strong and clear localization at the PM while mScarlet-TBP-SAAX mutant completely losses the ability to target PM. **e**, Statistical comparison between the PM targeting index of mScarlet-TBP-CAAX and mScarlet-TBP-SAAX (SAAX: n=42 line analysis of 14 independent cells; CAAX: n=45 line analysis of 15 independent cells); one-sided Student's t-test was used; see Methods for description of box plots. PM targeting index reveals the relative degree of the protein accumulating at the PM over the cytosol region and more detailed description of this index is given in the Method section. Scale bars: 10 μm.

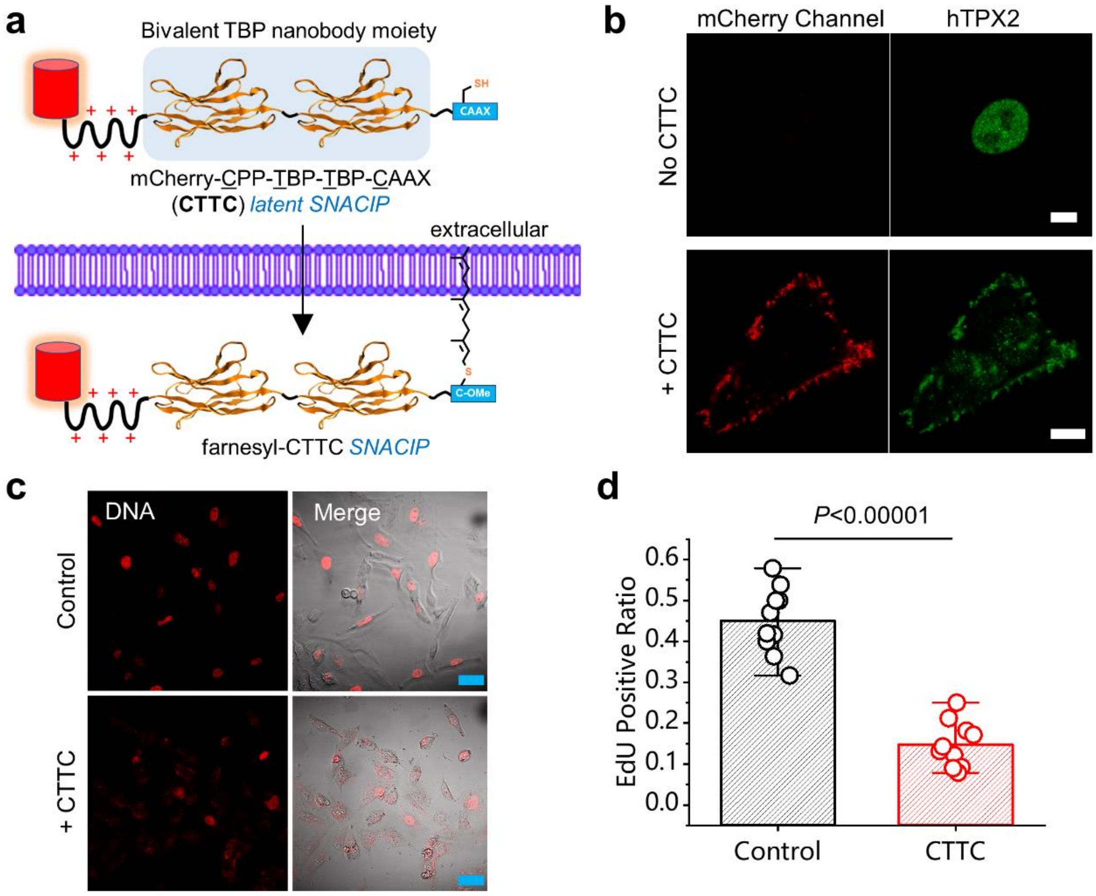

Supplementary Figure 17| CTTC is also cell-permeable and effectively inhibits cancer cell proliferation. **a**, Structural elements of CTTC inducer that features a tandem TBP nanobody moiety, a linear Tat cell-penetrating peptide (YGRKKRRQRRR) and a C-terminal CAAX-box. CTTC will undergo prenylation after entering the HeLa cell and subsequently being converted to a functional prenyl-CTTC SNACIP inducer. The cationic surfacing[5] feature (14× Arg in the arginine-rich TBP-TBP module) together with a classic Tat CPP will render this molecule readily cell-permeable. **b**, Immunofluorescence (IF) microscopic images of endogenous hTPX2 before and after CTTC drug treatment showed that CTTC directed endogenous hTPX2 to the non-functional plasma membrane location of the HeLa cell; hTPX2 revealed nucleus localization tendency before adding drug due to the presence of nucleus localization signal (NLS) sequence on this protein. CTTC inducer was used to treat live HeLa cells (10 μM, 2 h, then washed), and subsequently the cell sample was subjected to IF staining. **c**, EdU cell proliferation assay images of HeLa cells treated (+ CTTC) or not treated (control) with 10 μM CTTC. **d**, Statistical analysis of the EdU positive ratio of HeLa cells treated (+ CTTC) or not treated (control) by CTTC (n=10 cells for both groups); bar graphs denote mean ± SD; one-sided Student's *t*-test was used. Scale bars: white, 10 μm; cyan, 50 μm.

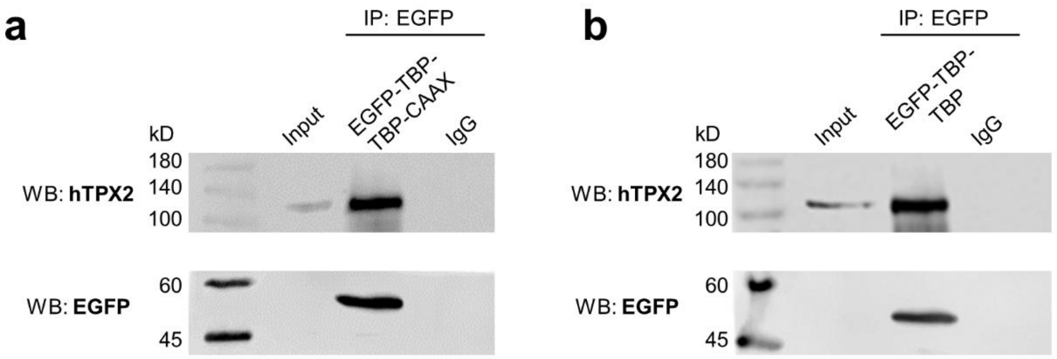

Supplementary Figure 18| Immunoprecipitation (IP) experiments show that TBP interacts with endogenous hTPX2. **a**, Live HeLa cells expressing EGFP-TBP-TPB-CAAX was subjected to cell lysis, incubated with EGFP antibody coated magnetic beads, and subjected to IP against hTPX2. IP result showed that EGFP-TBP-TBP-CAAX interacts with endogenous TPX2. **b**, Similar IP experiments as in **a**, showed that EGFP-TBP-TBP also interacts with endogenous hTPX2.

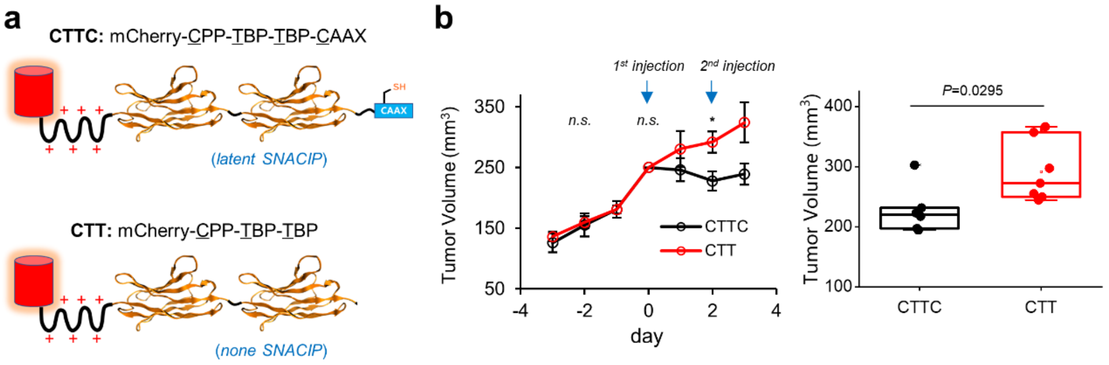

Supplementary Figure 19| CTTC displays a higher tumor suppression effect than the non-SNACIP type bivalent nanobody chimera CTT. a, CTTC possess a CAAX-box for prenylation whereas the bivalent nanobody chimera CTT is absent of CAAX-box and cannot be converted to a functional SNACIP inducer. **b**, Hepatocarcinoma xenograft mice were injected with ~0.08 ml of 22 mg·ml^{-1} (0.35 mM) of CTTC (n=6 mice from 2 independent experiments) or 20 mg·ml^{-1} (0.35 mM) of CTT (n=7 mice from 2 independent experiments), and the injections were repeated every two days to compensate for metabolic consumption; normalized tumor volumes were plotted against time and the tumor volumes for CTTC and CTT groups at the 2nd day after initial drug injection (denoted as day 0) were statistically compared using a boxplot. One-sided Student's *t*-test was used, *P*=0.0295; bar graphs denote mean ± SEM, and see Methods for description of box plots.

SUPPLEMENTARY METHODS

Synthesis and characterization of chemical compounds

General

Unless otherwise specified, all chemicals were purchased from commercial vendors and were used without further purification. The ^{1}H- and ^{13}C-NMR spectra were measured on a 400 MHz or 600 MHz Bruker BioSpin GmbH magnetic resonance spectrometer. Data for ^{1}H-NMR spectra are reported as follows: Chemical shifts are reported as δ in units of parts per million (ppm); multiplicities are reported as follows: s (singlet), d (doublet), t (triplet), q (quartet), dd (doublet of doublets), m (multiplet), or br (broadened); coupling constants are reported as J values in Hertz (Hz); the number of protons (n) for a given resonance is indicated as nH, and is based on the spectra integration values. High resolution mass spectra (HR-MS) measurement was performed via a customer service using electron spray ionization (ESI).

Synthesis of CysTMP (1) and related key intermediates

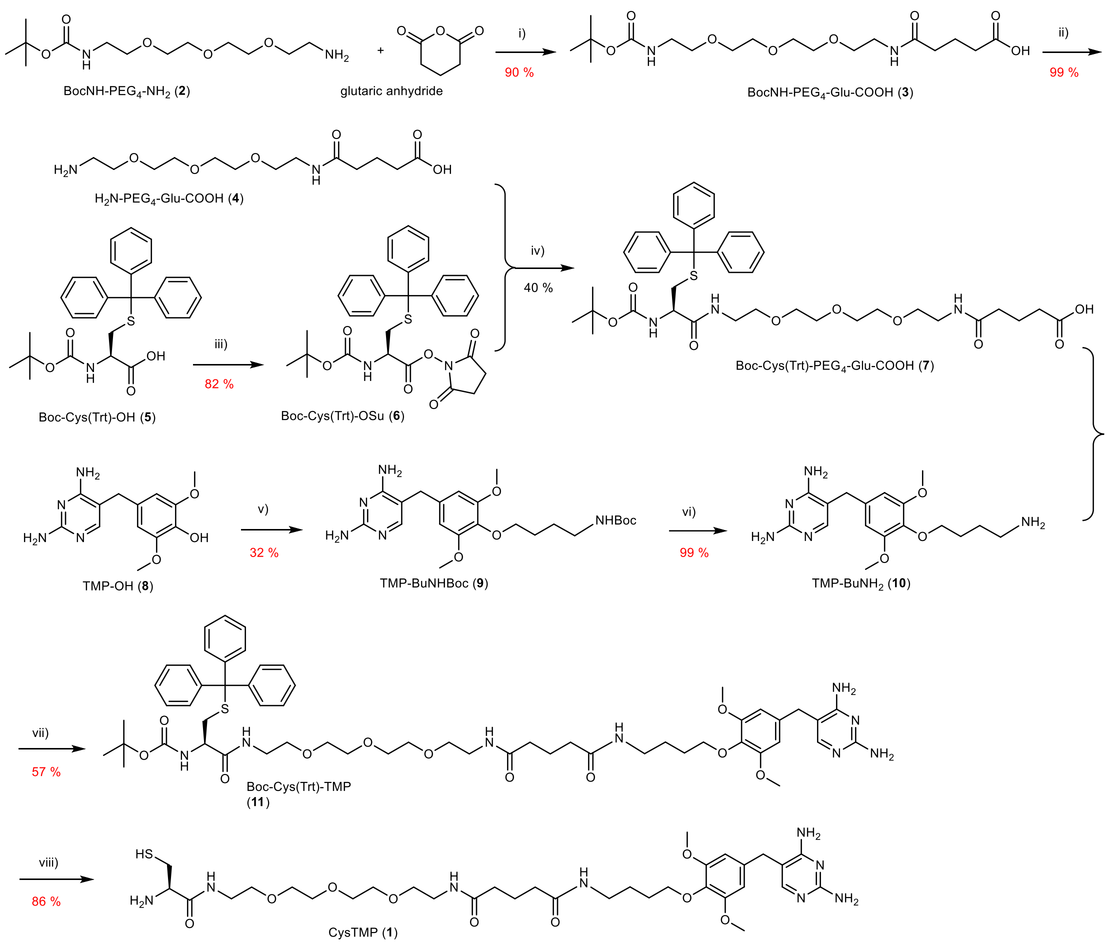

Synthetic scheme for the synthesis of Cys-TMP (**1**). i) DIEA, THF, RT, ON, 90%; ii) TFA, DCM, RT, 30 min, 99%; iii) HBTU, NHS, DIEA, DCM, 2h, 82%; iv) DIEA, THF, RT, ON, 40%; v) tert-butyl 4-bromobutylcarbamate, Cs_2CO_3, NaI·$2H_2O$, DMF, RT, 20h, 32%; vi) TFA/DCM (v:v 1:2), RT, 30min, then aq. Na_2CO_3, 99%; vii) HATU, DIEA, DCM, RT, ON, 57%; viii) TFA, 2.5%w TIS, RT, 1h, 86%. **Abbreviations:** TMP, trimethoprim; DIEA, N,N-diisopropylethylamine; THF, tetrahydrofuran; RT, room temperature; ON, overnight; TFA, trifluoroacetic acid; DCM, dichloromethane; HBTU, 2-(1H-Benzotriazole-1-yl)-1,1,3,3-tetramethyluronium hexafluorophosphate; NHS, N-hydroxysuccinimide; DMF, N,N-dimethylformamide; HATU, 1-[bis(dimethylamino)methylene]-1H-1,2,3-triazolo(4,5-b)pyridinium 3-oxide hexafluorophosphate; TIS, triisopropylsilane.

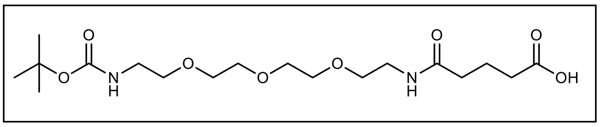

5-(2-(2-(2-(2-(tert-butoxycarbonyl)ethoxy)ethoxy)ethoxy)ethylamino)-5-

oxopentanoic acid (BocNH-PEG$_4$-Glu-COOH, **3**). BocNH-PEG$_4$-NH$_2$ (1.2 g, 4.11 mmol), glutaric anhydride (468 mg, 4.11 mmol) were dissolved in anhydrous THF (21 ml) and DIEA (636 mg, 4.93 mmol) were added. The reaction solution was stirred at RT overnight. The reaction solution was partitioned in EtOAc/aq. NaH_2PO_4 (2M). The organic phase was separated and the aqueous phase was extracted two additional times by EtOAc. Organic layers were combined, washed with brine, dried over anhydrous Na_2SO_4, filtered, concentrated and purified via silica gel chromatography (EtOAc/MeOH 8/1, Rf 0.4-0.6 (tailing), then EtOAc/MeOH 5/1) to yield 1.5 g product in a yield of 90%. **^{1}H-NMR** ($CDCl_3$, 400MHz): δ 6.51 (s, 1H), 5.16 (s, 1H), 3.63 (br, 8H), 3.53 (m, 4H), 3.44 (m, 2H), 3.29 (br, 2H), 2.37 (t, *J*=6.8Hz, 2H), 2.28 (t, *J*=7.4Hz, 2H), 1.94 (m, 2H), 1.42 (s, 9H); **^{13}C-NMR** ($CDCl_3$, 101MHz): δ 176.00, 172.91, 156.30, 79.53, 77.36, 70.61, 70.50, 70.29, 69.95, 40.41, 39.33, 35.32, 32.92, 28.51; **HRMS:** $C_{18}H_{35}N_2O_8^+$ $[M+H]^+$ calc. 407.2393, found 407.2393.

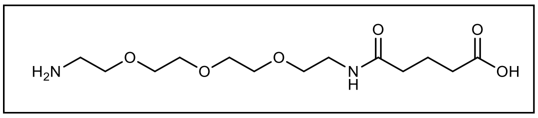

5-(2-(2-(2-(2-aminoethoxy)ethoxy)ethoxy)ethylamino)-5-oxopentanoic acid (H_2N-PEG$_4$-Glu-COOH, **4**). BocNH-PEG$_4$-Glu-COOH (406 mg, 1 mmol) was dissolved in anhydrous DCM (2 ml) and TFA (1 ml) was added. The reaction solution was stirred under Ar for 30 min to allow complete deprotection. DCM, TFA and other volatile constituents were removed under high vacuum to give ~423 mg of fully deprotected product nTFA·H_2N-PEG$_4$-Glu-COOH (~1 mmol) in a quantitative yield. **^{1}H-NMR** ($CDCl_3$, 400MHz): δ 7.94 (br, 1H), 7.90 (br, 1H), 4.66 (br, 3H), 3.79 (m, 2H), 3.70 (m, 2H), 3.62 (m, 6H), 3.56 (m, 2H), 3.43 (br, 2H), 3.21 (br, 2H), 2.37 (m, 2H), 2.29 (m, 2H), 1.93 (m, 2H); **^{13}C-NMR** ($CDCl_3$, 101MHz): δ 176.88, 174.36, 77.36, 70.33, 70.22, 69.96, 69.89, 69.75, 39.94, 39.48, 35.12, 33.09, 20.98; **HRMS:** $C_{13}H_{27}N_2O_6^+$ $[M+H]^+$ calc. 307.1869, found 307.1868.

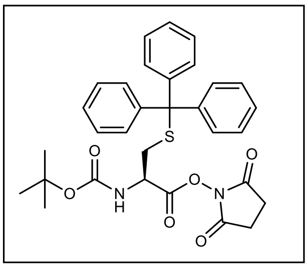

(R)-2-(tert-butoxycarbonyl)-3-(tritylthio)propanoic acid N-hydroxysuccinimidyl ester (BocCys(Trt)-OSu, **6**). BocCys(Trt)-OH (464 mg, 1 mmol) and HBTU (417 mg, 1.1 mmol) were dissolved in anhydrous DCM (10 ml) and stirred at RT for 10 min. Afterwards, DIEA (206 mg, 1.6 mmol) was added and the reaction solution was stirred at RT for another 10 min. Finally, NHS (127 mg, 1.1 mmol) was added and the reaction solution was allowed to stir for 2-3 hours. Thin layer chromatography (TLC) (cyclohexane/EtOAc 2/1, Rf 0.4) revealed full conversion of BocCys(Trt)-OH. The reaction solution was partitioned in EtOAc/ aq. NaH_2PO_4 (2M). The organic phase was separated and the aqueous phase was extracted one more time by EtOAc. All organic layers were combined, washed with brine, dried over anhydrous Na_2SO_4, filtered, concentrated and dried in vacuo to give the crude activated ester product. This NHS ester crude product hardly dissolves in cyclohexane, $CHCl_3$ nor benzene but could be easily dissolved in EtOAc. Hence, the crude product was first dissolved in EtOAc and then added with cyclohexane to give an emulsion which could be loaded onto a silica gel column. The product was purified via step-gradient elution using cyclohexane, cyclohexane/EtOAc 4/1, 3/1, and finally 2/1 to give 459 mg white foamy solid as the product in a yield of 82 %. **^{1}H-NMR** (DMSO-d^6, 400MHz): δ 7.67 (d, J=8.32Hz, 1H), 7.33 (m, 12H), 7.26 (m, 3H), 3.91 (m, 1H), 3.32 (s, 2H), 2.75 (s, 4H), 1.38 (s, 9H); **^{13}C-NMR** (DMSO-d^6, 101MHz): δ 169.60, 167.00, 154.83, 143.94, 129.00, 128.15, 126.89, 78.93, 66.71, 51.67, 32.28, 28.03, 25.37; **HRMS:** $C_{31}H_{33}N_2O_6S^+$ [M+H]$^+$ calc. 561.2059, found 561.2057.

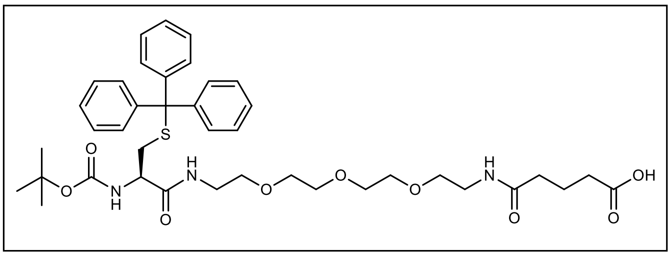

(R)-5-(2-(2-(2-(2-(2-(tert-butoxycarbonyl)-3-(tritylthio)propanamido)ethoxy)ethoxy)ethoxy) ethylamino)-5-oxopentanoic acid (BocCys(Trt)-PEG$_4$-Glu-COOH, **7**). BocCys(Trt)-OSu (440 mg, 0.78 mmol) was readily dissolved in THF (4.5 ml) and then added dropwise into a stirring alkanoic solution of nTFA·H$_2$N-PEG$_4$-Glu-COOH (423 mg, ~1 mmol) and DIEA (387 mg, 3 mmol) in anhydrous THF (3.5 ml). The reaction solution was stirred at RT for 8 hours. Then the reaction solution was partitioned in EtOAc/aq. NaH$_2$PO$_4$ (2M). The organic phase was separated and the aqueous phase was extracted two additional times by EtOAc. All organic layers were combined, washed four times by 2M aq. NaH$_2$PO$_4$, to reduce the amount of NHS byproduct and the excess of the starting material—H$_2$N-PEG$_4$-Glu-COOH to trace level. The organic layer was washed with brine, dried over anhydrous Na$_2$SO$_4$, filtered, concentrated, dissolved in EtOAc (slowly) and purified by silica gel chromatography via step gradient elution (EtOAc, then EtOAc/MeOH 20/1, and finally EtOAc/MeOH 15/1) to give 235 mg of white foamy solid as the product in a yield of 40 %. **^{1}H-NMR** (DMSO-d^6, 400MHz): δ 12.00 (s, br, 1H), 7.84 (t, *J*=5.76Hz, 1H), 7.74 (t, *J*=5.40Hz, 1H), 7.36-7.20 (m, 15H), 6.87 (d, *J*=8.36Hz, 1H); 3.91 (m, 1H), 3.48 (m, 10H), 3.38 (t, *J*=5.92Hz, 2H), 3.17 (m, 4H), 2.32 (d, *J*=7.08Hz, 2H), 2.19 (t, *J*=7.44Hz, 2H), 2.09 (t, *J*=7.44Hz, 2H), 1.69 (m, 2H), 1.37 (s, 9H); **^{13}C-NMR** (DMSO-d^6, 101MHz): δ 174.16, 171.70, 170.08, 144.32, 129.08, 128.02, 126.75, 78.37, 69.71, 69.60, 69.53, 69.10, 68.86, 65.86, 53.39, 38.44, 34.37, 32.99, 28.11, 20.67; **HRMS:** $C_{40}H_{54}N_3O_9S^+$ [M+H]$^+$ calc. 752.3581, found 752.3582.

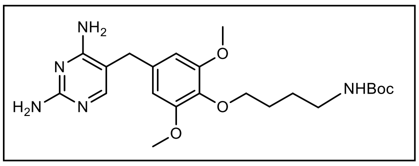

tert-butyl 4-(4-((2,4-diaminopyrimidin-5-yl)methyl)-2,6-dimethoxyphenoxy)butylcarbamate (TMP-Bu-NHBoc, **9**). Dimethoprim, *i.e.* TMP-OH (**8**) was prepared from TMP by demethylation using 47% HBr at 100 ^{o}C for 20 min (Chen *et al., Chem. Commun.* 2015, 51, 16537). Then dimethoprim (1.1 g, 4 mmol), 4-Boc-1-bromobutylamine (1.06 g, 4.2 mmol), Cs$_2$CO$_3$ (2.74 g, 8.4 mmol), and NaI·2H$_2$O (0.6 g, 4 mmol) were suspended in anhydrous DMF (20 ml). The reaction mixture was stirred at RT

under Ar for 20 hours. TLC ($CHCl_3$/MeOH 10/1, Rf = 0.4, visualized via UV/ninhydrin/PMA) revealed the completion of the reaction. The reaction mixture was partitioned in EtOAc/H_2O, and the aqueous phase was extracted three additional times by EtOAc. All organic layers were combined, washed with brine, dried over Na_2SO_4, filtered, concentrated and purified via silica gel chromatography (eluted sequentially by EtOAc, $CHCl_3$, and then $CHCl_3$/MeOH 10/1); the crude product was purified by a second silica gel chromatography ($CHCl_3$/MeOH 10/1, Rf 0.4) to yield 570 mg light yellow solid as the product in a yield of 32% with high purify. **^{1}H-NMR** (DMSO-d^6, 400MHz): : δ 7.51 (s, 1H), 6.77 (t, J=5.84, 1H), 6.54 (s, 2H), 6.08 (s, 2H), 5.69 (s, 2H), 3.77 (t, J=6.06Hz, 2H), 3.71 (s, 6H), 3.52 (s, 2H), 2.95 (m, 2H), 1.53 (m, 4H), 1.37 (s, 9H); **^{13}C-NMR** (DMSO-d^6, 101MHz): δ 162.22, 162.19, 155.69, 155.58, 152.85, 135.86, 134.64, 105.86, 105.78, 77.28, 72.02, 55.82, 39.57, 32.97, 28.25, 27.01, 26.09; **HRMS:** $C_{22}H_{34}N_5O_5^+$ $[M+H]^+$ calc. 448.2560, found 448.2560.

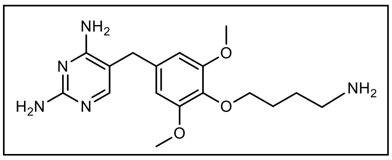

5-(4-(4-aminobutoxy)-3,5-dimethoxybenzyl)pyrimidine-2,4-diamine (TMP-Bu-NH_2, **10**). TMP-Bu-NHBoc (186 mg, 0.416 mmol) was dissolved in 1 ml of anhydrous DCM and TFA (0.5 ml) was added. The reaction solution was allowed to stir at RT for 1 hour to allow full deprotection. DCM and TFA and other volatile constituents were removed under high vacuum to give 144 mg of crude product in the form of trifluoroacetate salt. This crude product was further suspended in EtOAc/aq. Na_2CO_3 to liberate the free amine product. The organic phase was separated and the aqueous phase was extracted multiple times. Organic layers were combined, washed with brine, dried over anhydrous Na_2SO_4, filtered, concentrated and dried in vacuo to give 122 mg white solid as the amine product in a nearly quantitative yield. **^{1}H-NMR** (DMSO-d^6, 400MHz): δ 7.51 (s, 1H), 6.53 (s, 1H), 6.06 (s, 1H), 5.67 (s, 1H), 3.77 (t, J=6.36Hz, 2H), 3.71 (s, 6H), 2.55 (t, J=6.72Hz, 2H), 1.61 (m, 2H), 1.45 (m, 2H); **^{13}C-NMR** (DMSO-d^6, 101MHz): δ 162.24, 162.17, 155.72, 152.86, 135.60, 134.96, 105.90, 105.77, 72.36, 55.85, 41.41, 32.95, 29.72, 27.15; **HRMS:** $C_{17}H_{26}N_5O_3^+$ $[M+H]^+$ calc. 348.2036, found 348.2036.

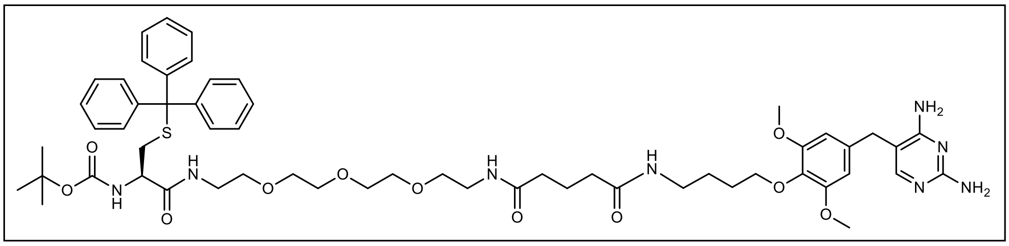

(R)-tert-butyl 1-(2-(2-(2-(2-(5-(4-(4-((2,4-diaminopyrimidin-5-yl)methyl)-2,6-dimethoxyphenoxy)butylamino)-5-oxopentanamido)ethoxy)ethoxy)ethoxy)ethylamino)-1-oxo-3-(tritylthio)propan-2-ylcarbamate (BocCys(Trt)-TMP, **11**). BocCys(Trt)-PEG$_4$-Glu-COOH (75.2 mg, 0.1 mmol) was dissolved in 1 ml of anhydrous DCM. Then 42 mg of HATU (0.11 mmol) and DIEA (36 mg, 0.28 mmol) was added and the reaction solution was allowed to stir for 5-10 min. TMP-Bu-NH$_2$ (35 mg, 0.1 mmol) was added and the reaction suspension was allowed to react at RT overnight to form a clear reaction solution. The reaction solution was partitioned in EtOAc/aq. Na_2CO_3. The organic layer was separated and the aqueous phase was extracted two additional times by EtOAc. Organic layers were combined, sequentially washed with aq. NaH_2PO_4 (2 M), aq. Na_2CO_3 and brine. Some yellowish PPT may form during washing steps and the PPT should be treated as the precipitated product. The combined organic phase was dried over anhydrous Na_2SO_4, filtered, concentrated and purified by silica gel chromatography via step-gradient elution ($CHCl_3$/MeOH 10/1, 8/1, and finally 5/1, Rf ($CHCl_3$/MeOH 5/1) = 0.5) to give 61.2 mg light yellow product in a yield of 57 %. **^{1}H-NMR** (DMSO-d^6, 600MHz): δ 7.84 (t, *J*=5.88Hz, 1H), 7.77 (m, 2H), 7.49 (s, 1H), 7.32 (t, *J*=7.5Hz, 6H), 7.28 (d, *J*=7.92Hz, 6H), 7.24 (t, *J*=7.08Hz, 3H), 6.92 (d, *J*=8.52Hz, 1H), 6.54 (s, 1H), 6.38 (br, 2H), 5.97 (s, 2H), 4.12 (s, 2H), 3.92 (m, 1H), 3.78 (t, *J*=6.0Hz, 2H), 3.70 (s, 6H), 3.52 (s, 2H), 3.47 (br, 4H), 3.45 (br, 4H), 3.19 (m, 1H), 3.12 (m, 1H), 3.05 (m, 2H), 2.04 (m, 2H), 1.55 (m, 2H), 1.53 (m, 2H), 1.37 (s, 9H); **^{13}C-NMR** (DMSO-d^6, 151MHz): δ 171.88, 171.58, 170.15, 162.51, 161.00, 154.92, 152.91, 144.35, 135.28, 134.88, 129.12, 128.08, 128.79, 106.31, 105.88, 78.39, 72.00, 69.74, 69.63, 69.56, 69.16, 68.89, 65.87, 55.85, 53.41, 40.06, 38.68, 38.45, 38.17, 34.84, 34.77, 34.06, 32.88, 28.15, 27.16, 25.76, 21.63; **HRMS:** $C_{57}H_{77}N_8O_{11}S^+$ [M+H]$^+$ calc. 1081.5427, found 1081.5471.

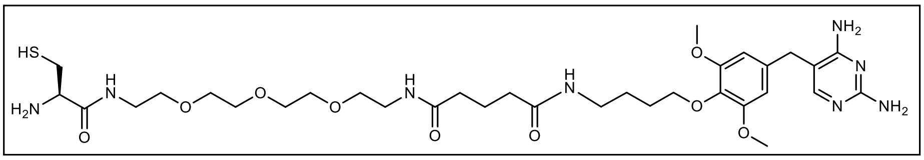

(R)-N1-(2-(2-(2-(2-(2-amino-3-mercaptopropanamido)ethoxy)ethoxy)ethoxy)ethyl)-N5-(4-(4-((2,4-diaminopyrimidin-5-yl)methyl)-2,6-dimethoxyphenoxy)butyl)glutaramide (CysTMP, **1**). BocCys(Trt)-TMP (25 mg, 0.023 mmol) was dissolved in 1 ml of TFA to allow deprotection of Boc and Trt groups. The reaction solution would immediately turn yellowish which indicates the formation of triphenylmethyl radical. Then 25 µl TIS was added and the yellow color of the reaction solution will soon disappear; this indicates quenching of triphenylmethyl radical by TIS and helps to drive the deprotection of Trt to complete. The reaction solution was stirred under Ar for 1 h and TLC ($CHCl_3$/MeOH 5/1, Rf 0.2-3) revealed the completion of the reaction. Most TFA and TIS were removed under high vacuum and the residue was partitioned in EtOAc/H_2O. The aqueous phase was separated, washed two additional times by EtOAc, concentrated under reduced pressure and dried in vacuo to give 17 mg white foamy solid as the product in a yield of 86 %. **^{1}H-NMR** (DMSO-d^6, 400MHz): *δ* 8.54 (t, *J*=5.7Hz, 1H, 8.29 (br, 3H), 7.84 (t, *J*=5.6Hz, 1H), 7.77 (t, *J*=5.6Hz, 1H), 7.74 (s, 1H), 7.64 (s, br, 2H), 7.44 (s, 1H), 6.60 (s, 2H), 3.95 (t, *J*=5.6Hz, 1H), 3.79 (t, *J*=6.28Hz, 2H), 3.73 (s, 6H), 3.59 (s, 2H), 3.51 (br, 4H), 3.50 (br, 4H), 3.39 (t, *J*=6.1Hz, 2H), 3.34 (m, 1H), 3.26 (m, 1H), 3.18 (m, 2H), 3.06 (m, 2H), 2.90 (br, 2H), 2.05 (m, 4H), 1.68 (m, 2H), 1.57 (m, 4H); **^{13}C-NMR** (DMSO-d^6, 101MHz): *δ* 171.88, 171.57, 166.72, 164.06, 154.28, 153.06, 139.77, 135.33, 132.79, 108.92, 106.30, 71.99, 69.71, 69.58, 69.53, 69.09, 68.71, 55.92, 53.90, 38.40, 38.13, 34.83, 34.77, 32.08, 30.75, 27.12, 25.72, 25.13, 21.60; **HRMS:** $C_{33}H_{55}N_8O_9S^+$ $[M+H]^+$ calc. 739.3807, found 739.3820.

Synthesis of the CID Dimerizer—TMP-Cl (14)

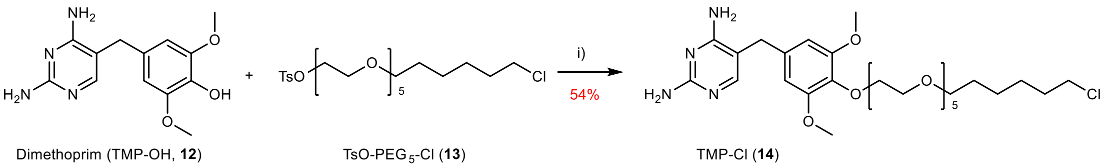

Dimethoprim (TMP-OH, **12**) TsO-PEG$_5$-Cl (**13**) TMP-Cl (**14**)

Synthetic scheme for the synthesis of TMP-Cl (**14**). i) Cs_2CO_3, DMF, RT, ON, Ar, 54%

yield.

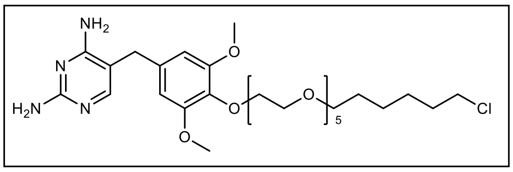

5-(4-((21-chloro-3,6,9,12,15-pentaoxahenicosyl)oxy)-3,5-dimethoxybenzyl) pyrimidine-2,4-diamine (TMP-Cl, **14**). TsO-PEG$_5$-Cl (**13**) was obtained following previously reported procedures (Chen, *et al. Angew. Chem. Int Ed.* **2017**, 56, 5916). Afterwards, TMP-OH (**12**, 50 mg, 0.18 mmol) was mixed with TsO-PEG$_5$-Cl (**13**, 97.2 mg, 0.19 mmol) and Cs_2CO_3 (76.3 mg, 0.234 mmol) in a 2-neck round bottom flask (RBF). Anhydrous DMF (1.8 ml) was added and the reaction suspension was rapidly stirred at RT under argon overnight to complete the coupling. DMF was removed under high vacuum. Methanol was added and the reaction residue was vacuumed again. This procedure was repeated three times to facilitate the complete removal of DMF. The dried residue was participated in EtOAc/Na_2CO_3 (aq.) and the organic layer was separated. The aqueous phase was extracted two additional times and all organic layers were combined, washed with brine (2×), dried over anhydrous Na_2SO_4, filtered, concentrated and purified using silica gel chromatography via gradient elution (EtOAc:MeOH 10:1→8:1→DCM:MeOH 10:1) to give 60.4 mg white solid as the product in a yield of 54%. The characterization data was in accordance with published in the same literature. **^{1}H-NMR** (DMSO-d^6, 600MHz): δ 7.50 (s, 1H), 6.54 (s, 2H), 6.11 (s, 2H), 5.72 (s, 2H), 3.90 (t, 2H, *J*=5.1Hz), 3.71 (s, 6H), 3.63-3.69 (m, 4H), 3.56 (m, 2H), 3.48-3.53 (m, 14H), 3.45 (m, 2H), 3.35 (t, 2H, t, *J*=6.48Hz), 1.70 (m, 2H), 1.47 (m, 2H), 1.37 (m, 2H), 1.29 (m, 2H). **MS**(ESI): $C_{29}H_{48}O_8N_4Cl^+$ $[M+H]^+$, calcd. 615.32, found 615.42.

Synthesis of AzidoCBT (ACBT, 17)

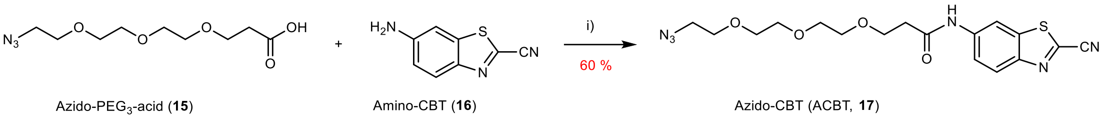

Synthetic scheme for the synthesis of ACBT (**17**). i) HATU, DIEA, DMF, RT, 1 day, 60% yield.

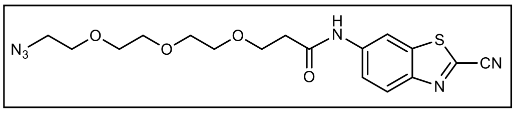

3-(2-(2-(2-Azidoethoxy)ethoxy)ethoxy)-N-(2-cyanobenzo[d]thiazol-6-yl)propenamide (ACBT, **17**). Azido-PEG$_3$-acid (33.5 mg, 0.136 mmol, **15**) was dissolved in 0.6 ml anhydrous DMF in a thoroughly dried RBF equipped with a stir bar. HATU (57.5 mg, 0.136 mmol) and DIEA (38 mg, 0.29 mmol) was added. The reaction suspension was stirred at RT under Ar for a few minutes to give a yellowish solution which indicates the activation of the carboxylate. Afterwards, Amino-CBT (20 mg, 0.113 mmol, **16**) was added. The reaction solution was stirred at RT under Ar for one day. The reaction mixture was partitioned in EtOAc/NaH_2PO_4 (2M). The organic layer was separated and the aqueous phase was extracted two additional times by EtOAc. Organic layers were combined, washed with sat. Na_2CO_3, filtered, concentrated, and purified via silica gel chromatography (EtOAc, Rf 0.25) to give a yellowish oil as the product in a yield of 60%. **^{1}H-NMR** (DMSO-d^6, 600MHz): δ 10.47 (s, 1H), 8.76 (t, J=2.22Hz, 1H), 8.18 (dd, J^1=8.94Hz, J^2=1.92Hz, 1H), 7.73 (d, J=9.00Hz, 1H), 3.73 (td, J^1=6.24Hz, J^2=1.98Hz, 2H), 3.48-3.55 (m, 11H), 3.33 (s, 2H), 2.63 (td, J^1=6.02Hz, J^2=1.98Hz, 2H); **^{13}C-NMR** (DMSO-d^6, 151MHz): 170.05, 147.55, 139.71, 136.79, 134.92, 124.83, 120.65, 113.66, 111.09, 69.81, 69.76, 69.74, 69.68, 69.24, 66.54, 49.97, 37.33; **HRMS**: $C_{17}H_{20}N_6O_4SNa^+$ $[M+Na]^+$ calcd. 427.1159, found 427.1151.

NMR SPECTRA

^{1}H-NMR (400MHz, $CDCl_3$) of BocNH-PEG$_4$-Glu-COOH (**3**)

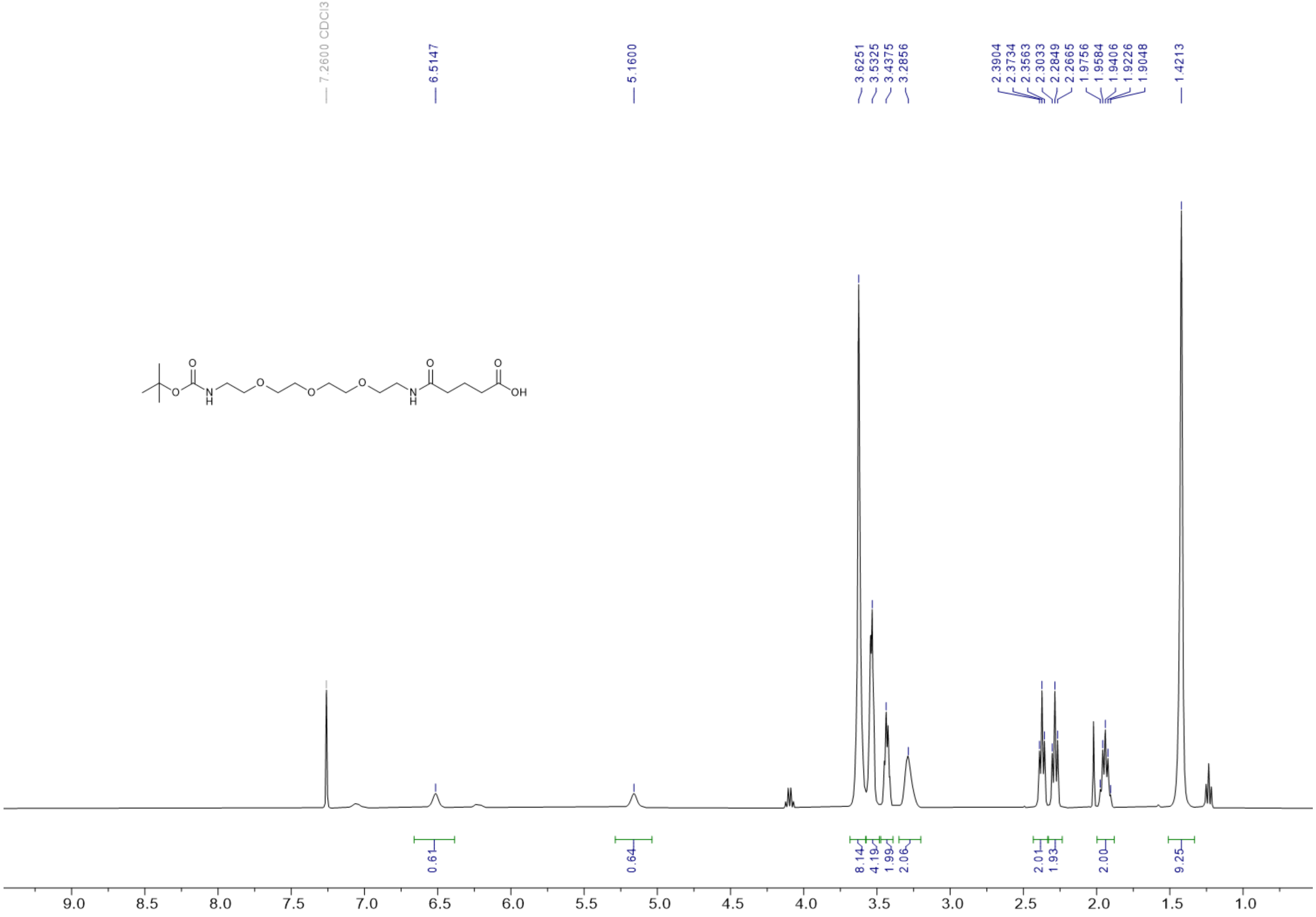

^{13}**C-NMR** (101MHz, $CDCl_3$) of BocNH-PEG$_4$-Glu-COOH (**3**)

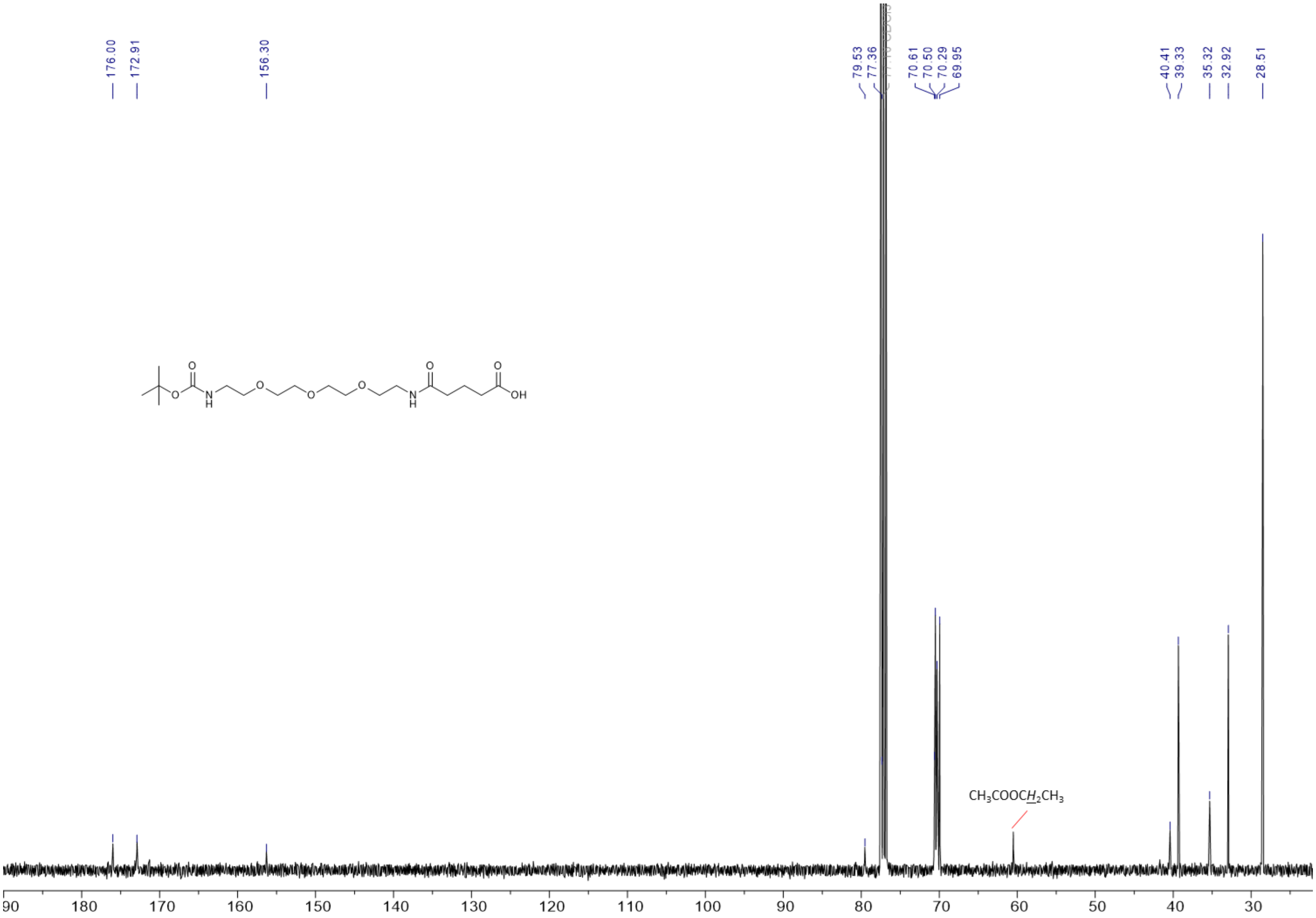

¹H-NMR (400MHz, DMSO-d⁶) of BocCys(Trt)-PEG₄-Glu-COOH (**7**)

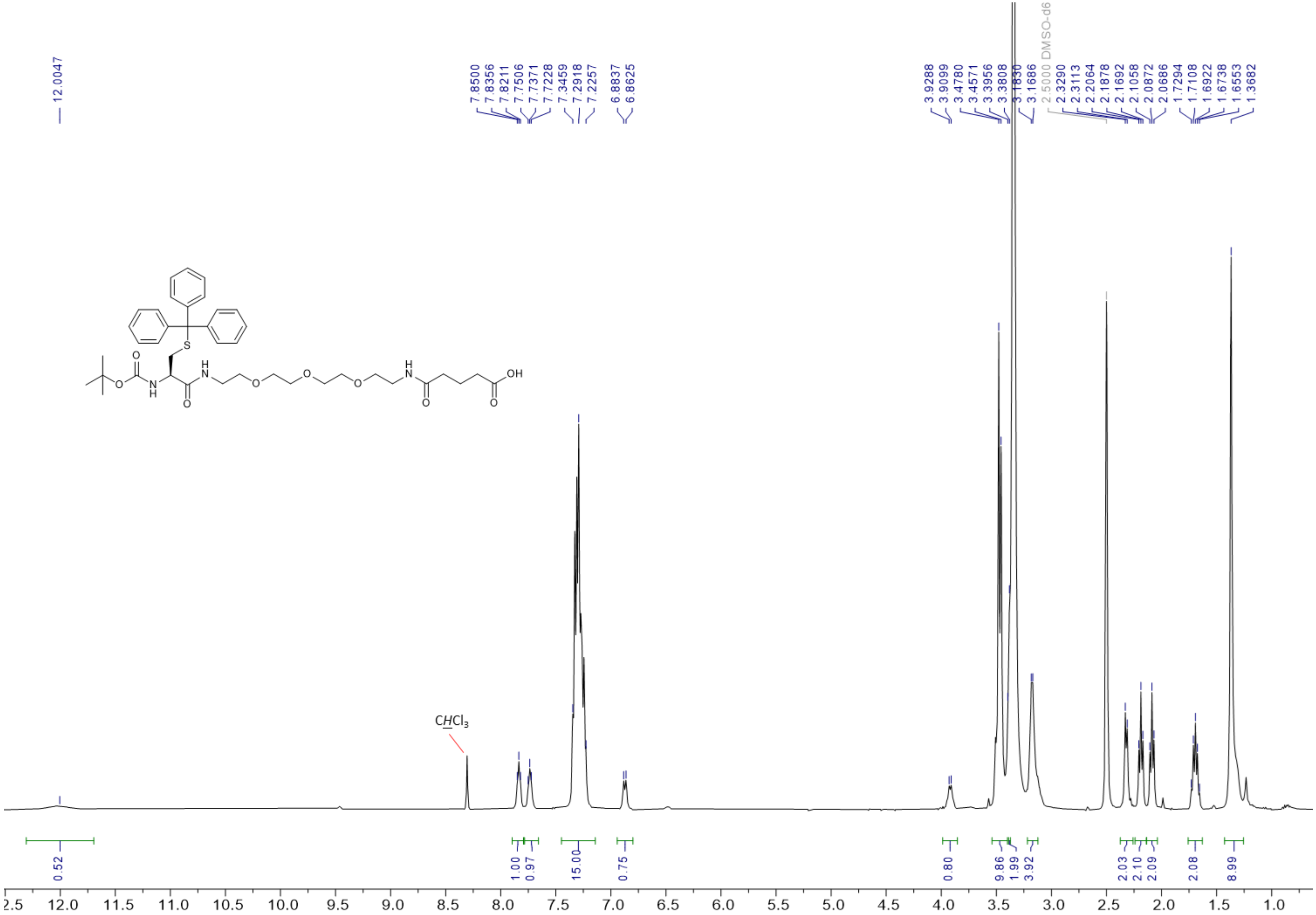

^{13}**C-NMR** (101MHz, DMSO-d^6) of BocCys(Trt)-PEG$_4$-Glu-COOH (**7**)

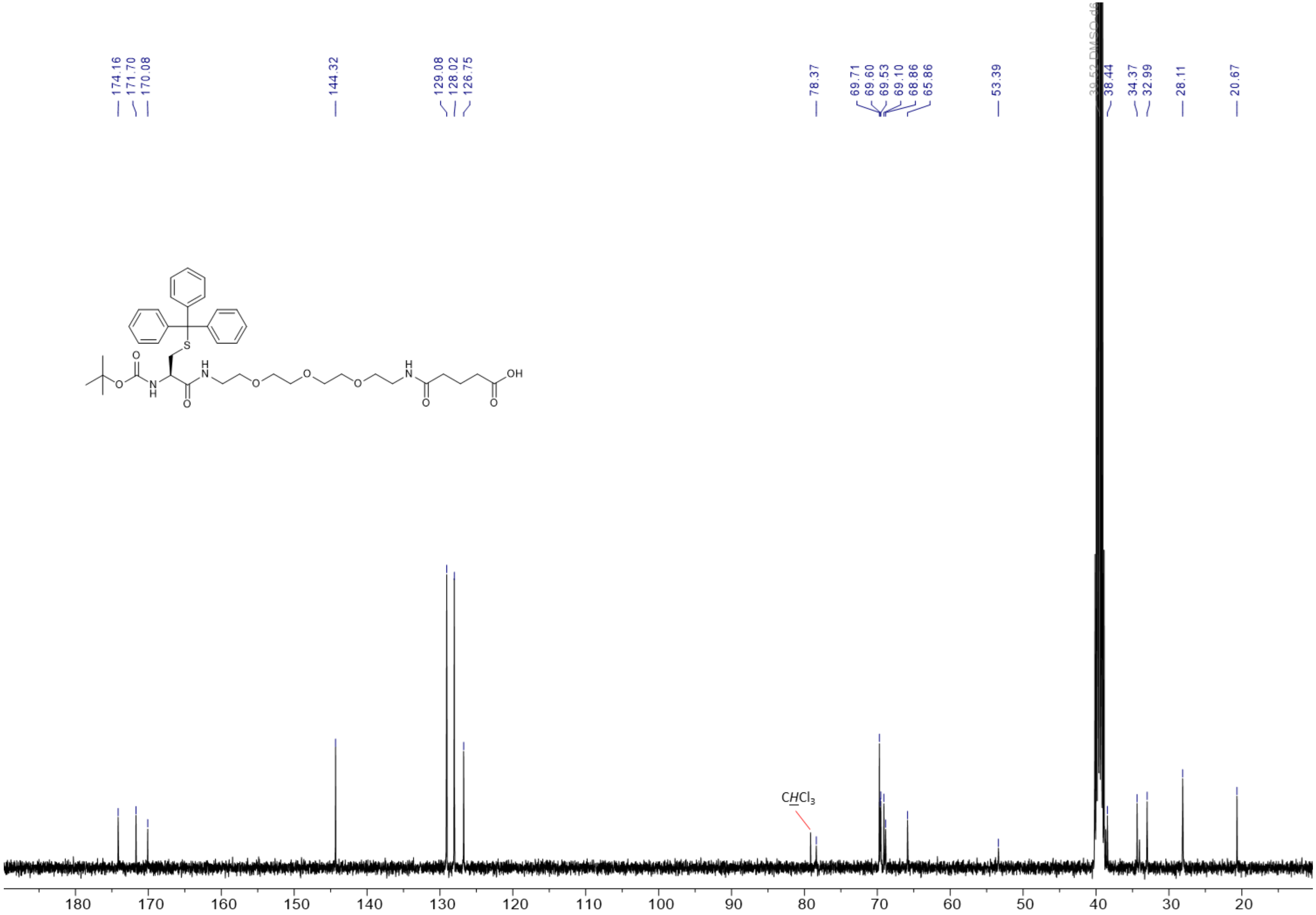

¹H-NMR (400MHz, DMSO-d⁶) of TMP-Bu-NHBoc (**9**)

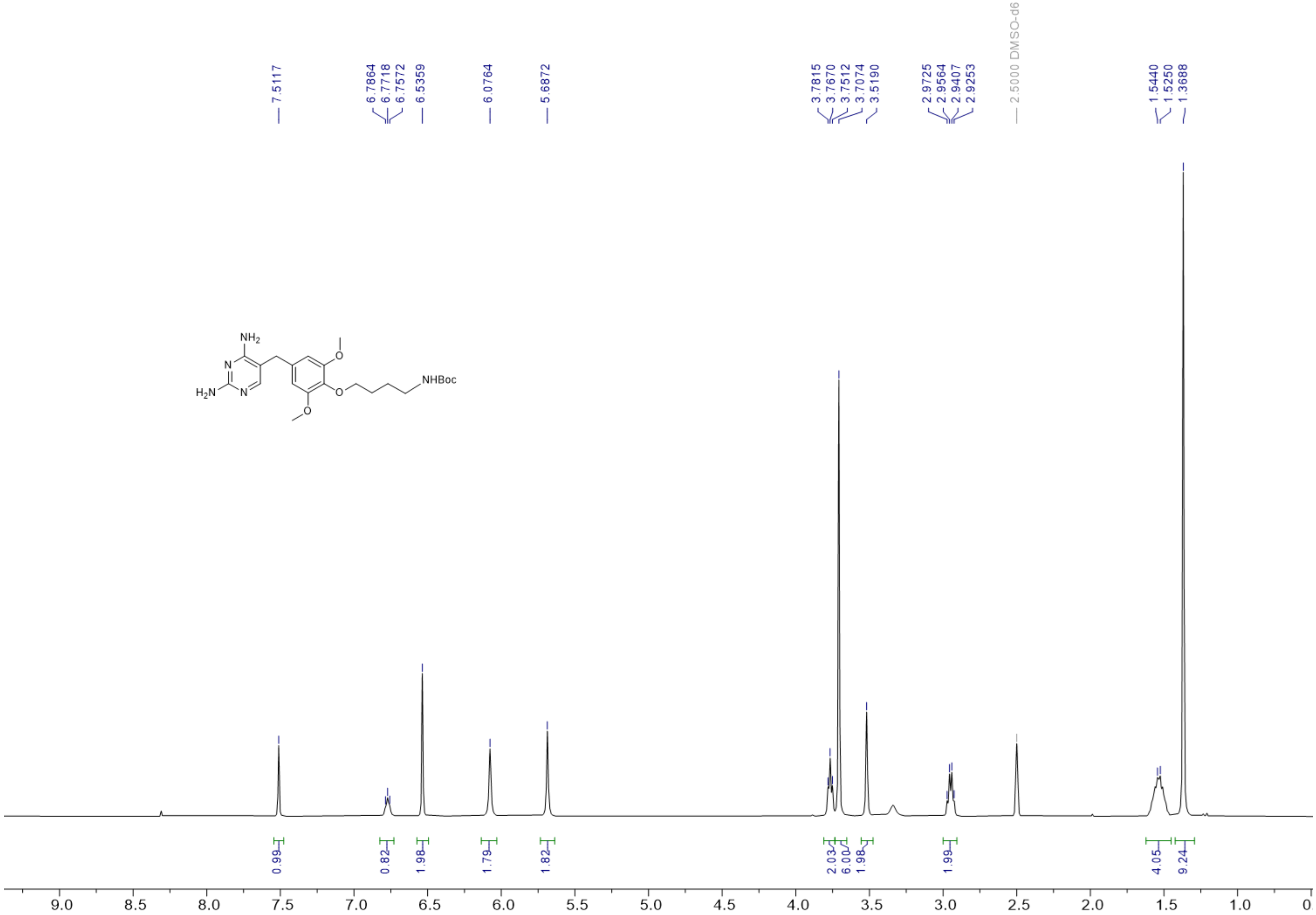

^{13}C-NMR (101MHz, DMSO-d^6) of TMP-Bu-NHBoc (**9**)

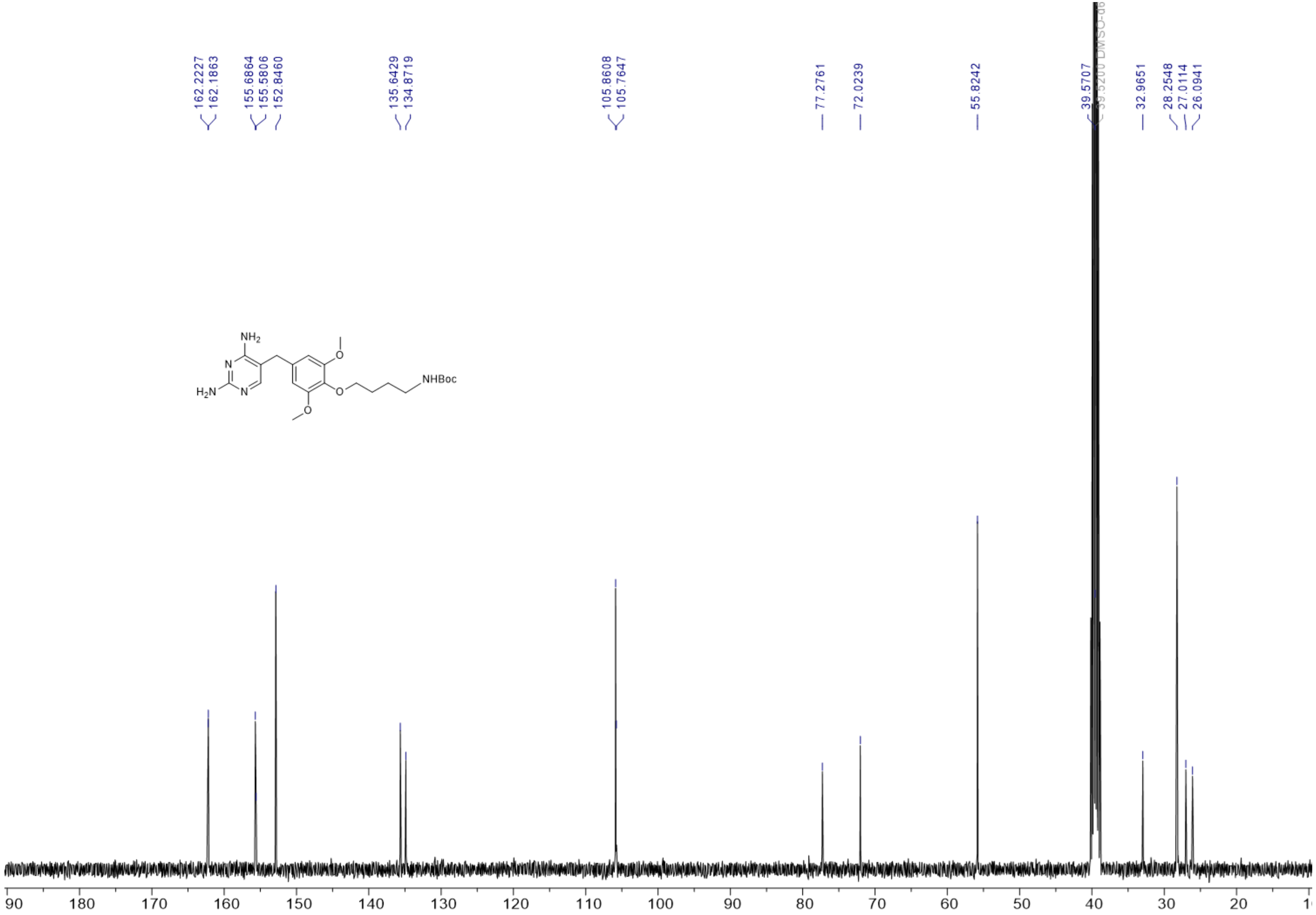

^{1}H-NMR (400MHz, DMSO-d[6]) of TMP-Bu-NH_2 (**10**)

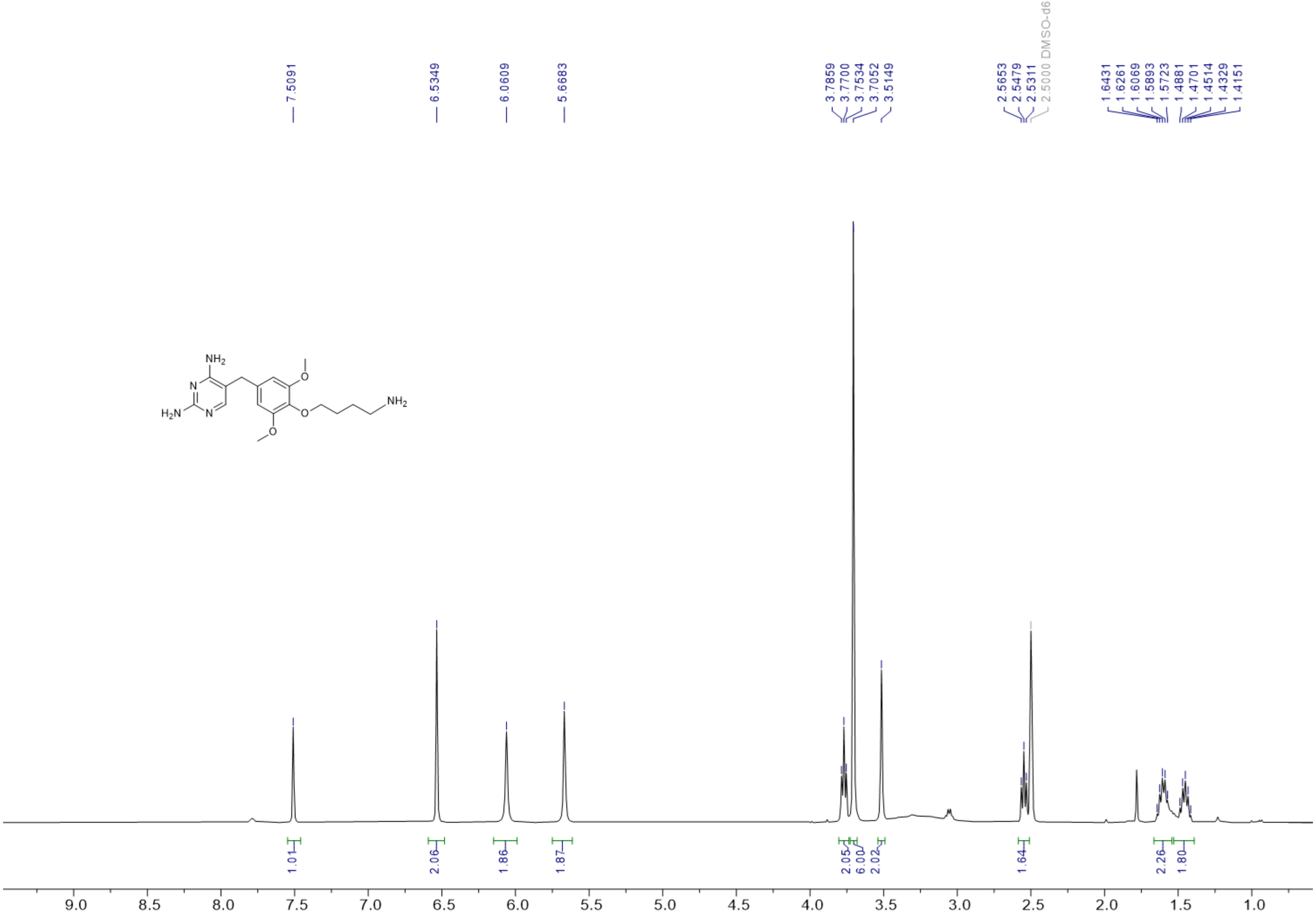

^{13}C-NMR (101MHz, DMSO-d^{6}) of TMP-Bu-NH_2 (**10**)

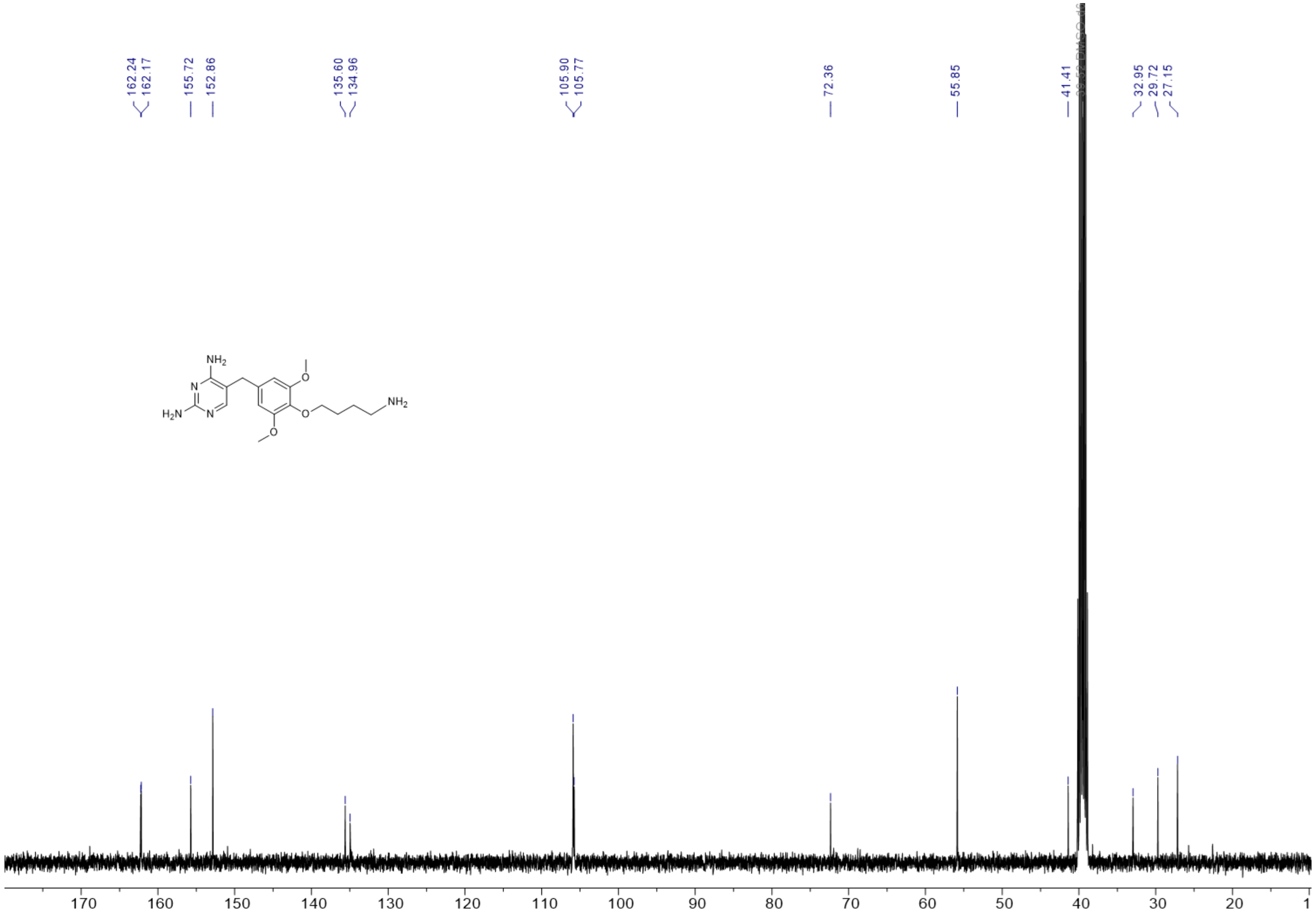

^{1}H-NMR (600MHz, DMSO-d^6) of BocCys(Trt)-TMP (**11**)

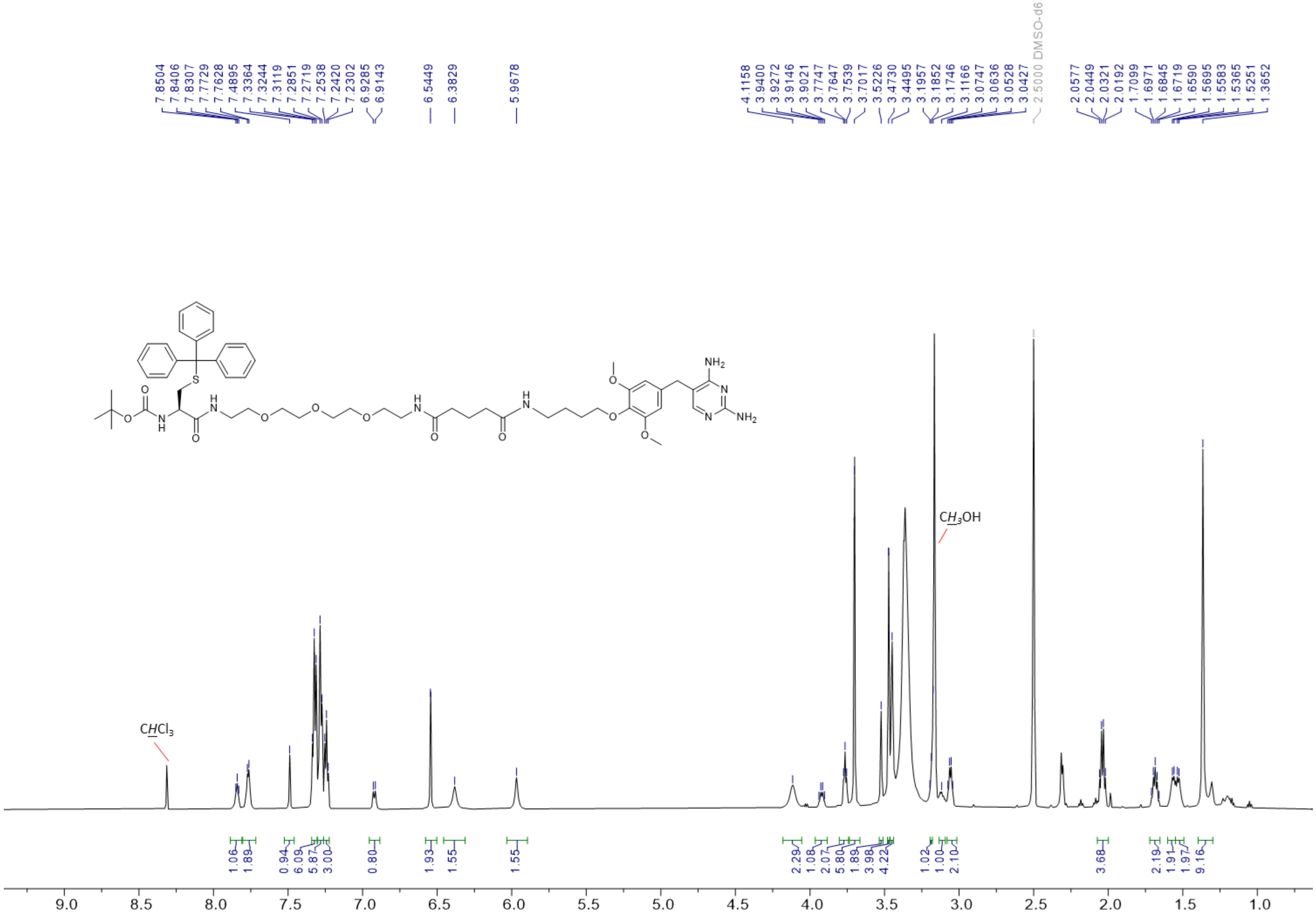

^{13}C-NMR (151MHz, DMSO-d^6) of BocCys(Trt)-TMP (**11**)

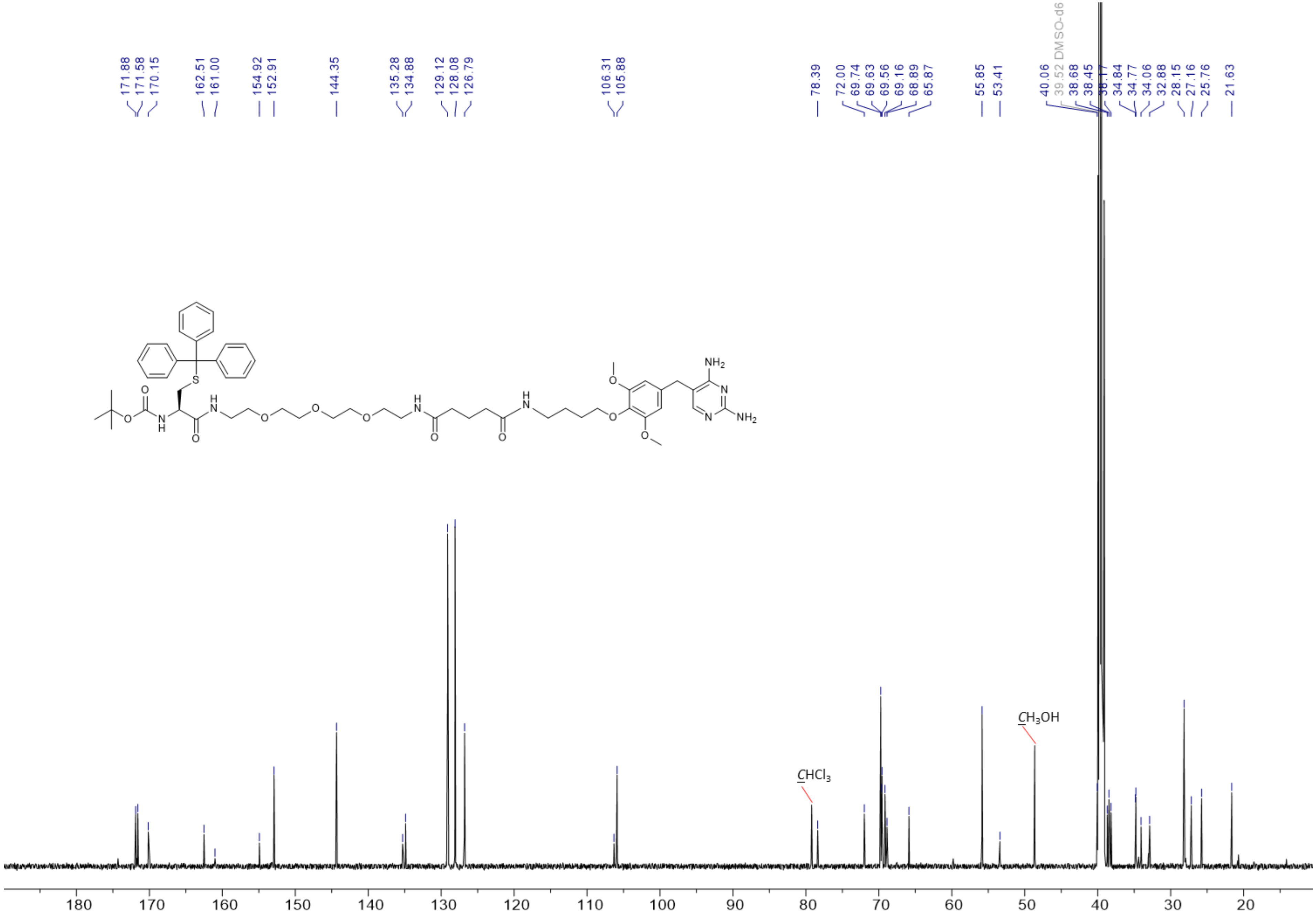

^{1}H-NMR (400MHz, DMSO-d^6) of Cys-TMP (**1**)

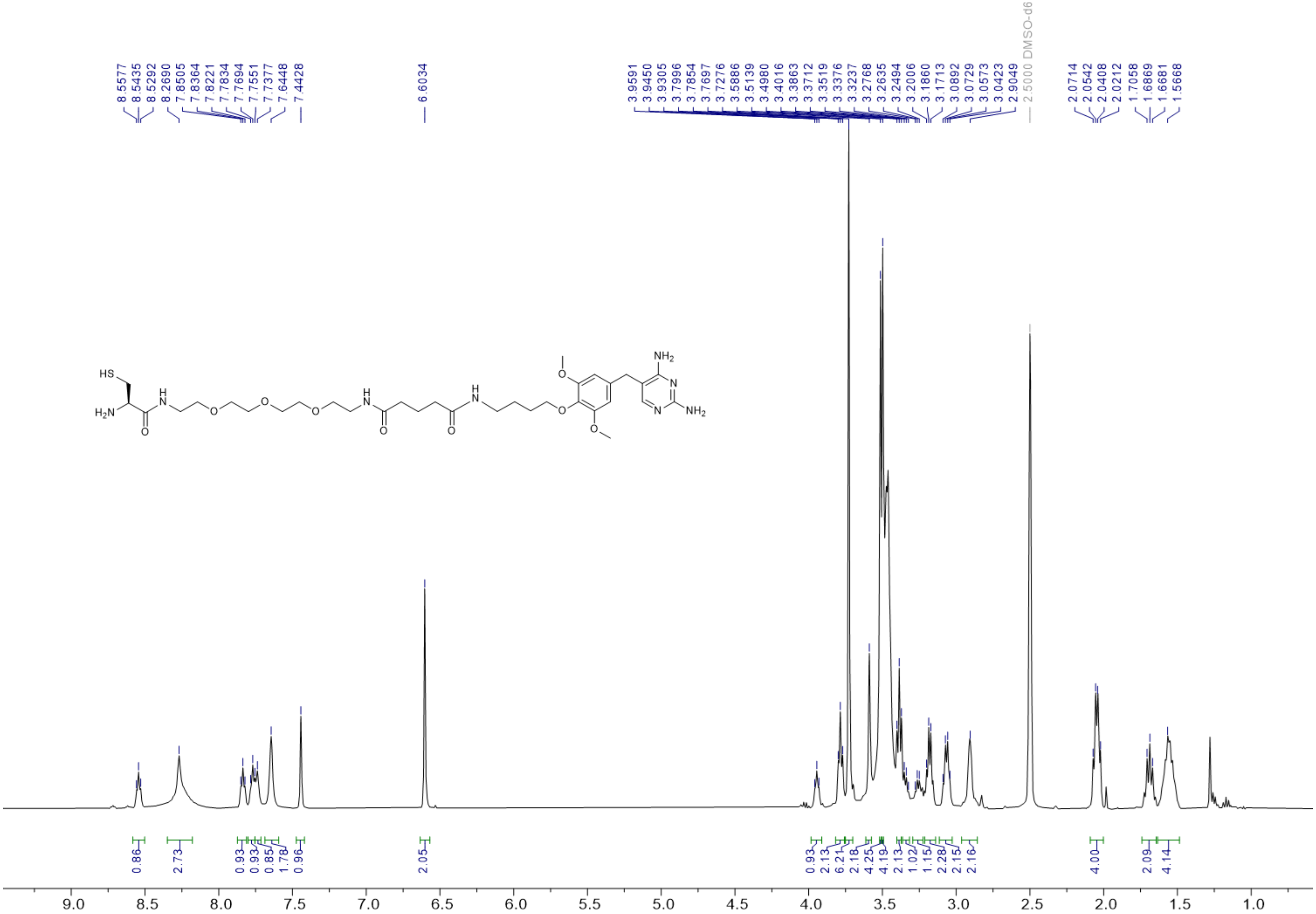

^{13}**C-NMR** (101MHz, DMSO-d^{6}) of Cys-TMP (**1**)

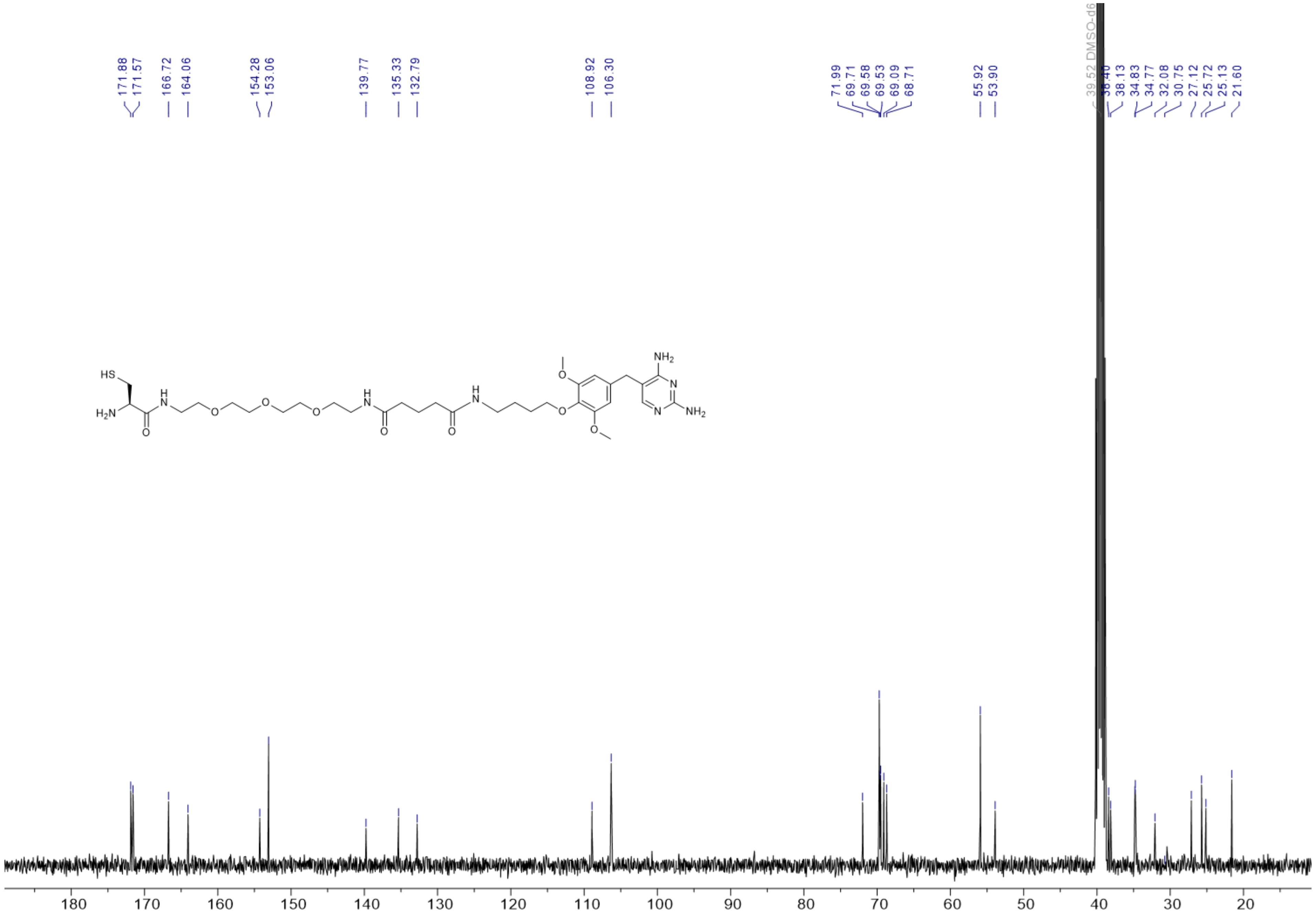

[1]**H-NMR** (600MHz, DMSO-d[6]) of ACBT (**17**)

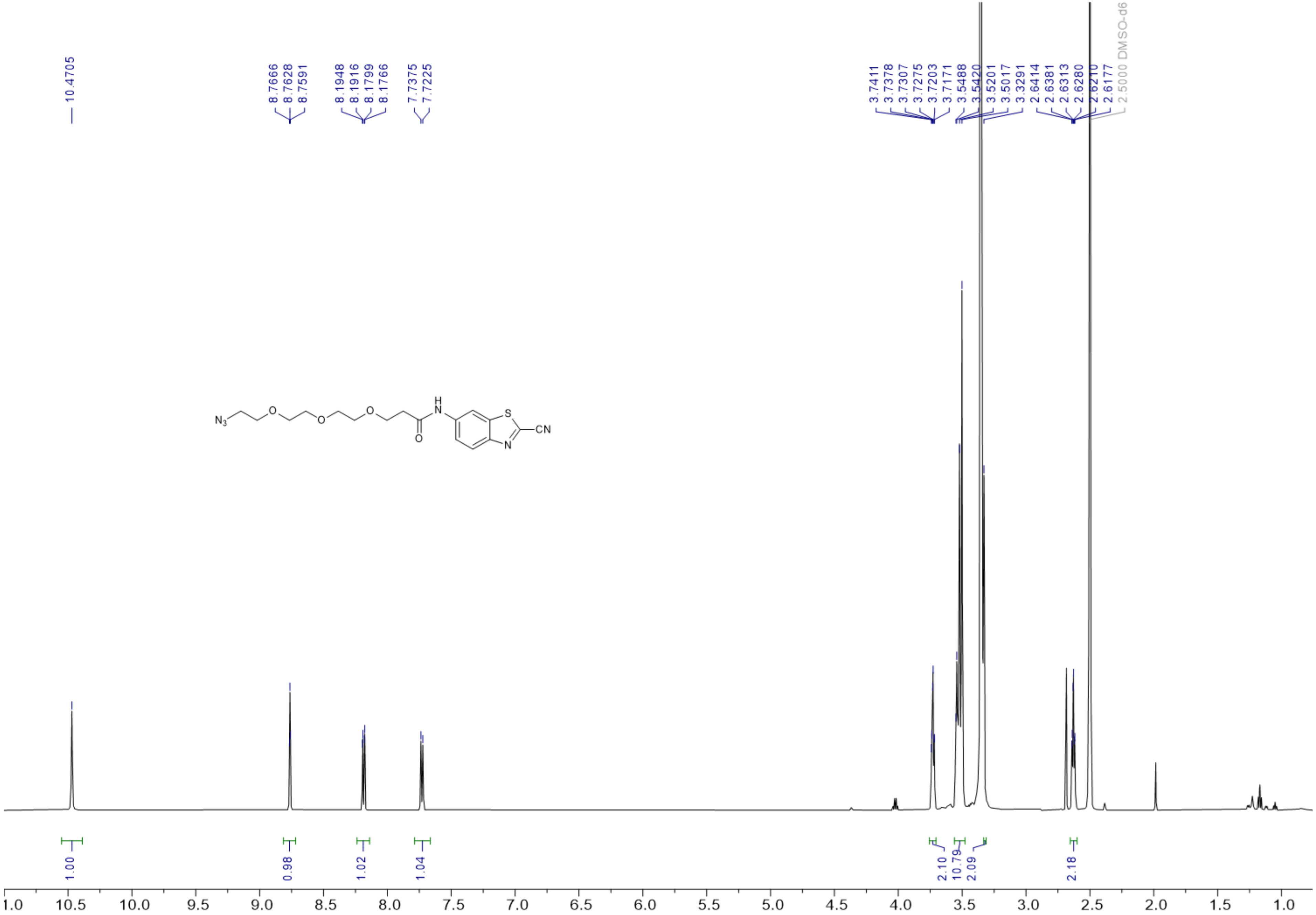

^{13}C-NMR (151MHz, DMSO-d^{6}) of ACBT (**17**)

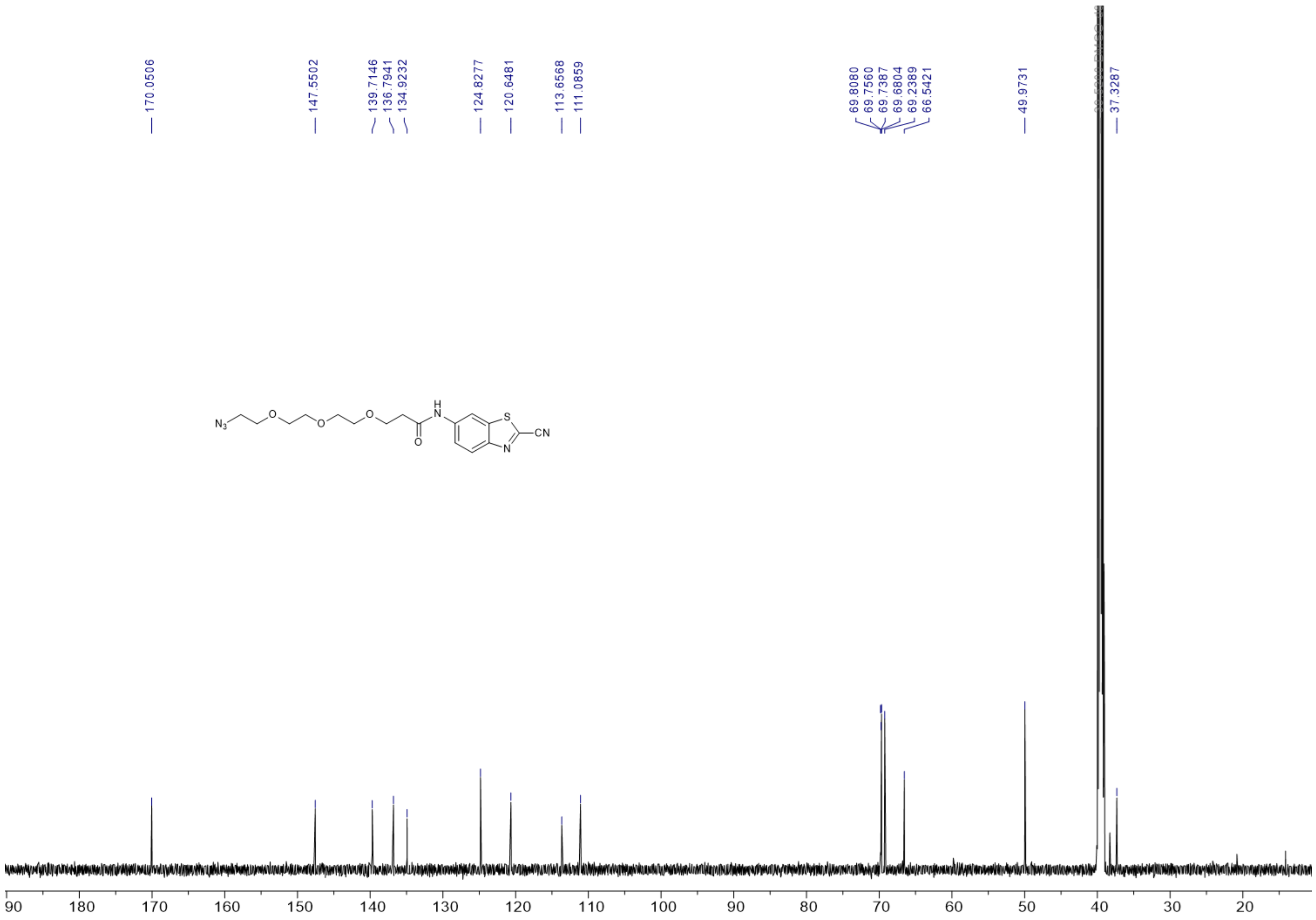

SUPPLEMENTARY REFERENCES:

1. Lattig-Tunnemann, G. et al. Backbone rigidity and static presentation of guanidinium groups increases cellular uptake of arginine-rich cell-penetrating peptides. *Nat Commun* **2** (2011).
2. Herce, H.D. et al. Cell-permeable nanobodies for targeted immunolabelling and antigen manipulation in living cells. *Nat. Chem.* **9**, 762-771 (2017).
3. Simamora, P., Alvarez, J.M. & Yalkowsky, S.H. Solubilization of rapamycin. *Int. J. Pharmaceut.* **213**, 25-29 (2001).
4. Banaszynski, L.A., Liu, C.W. & Wandless, T.J. Characterization of the FKBP.rapamycin.FRB ternary complex. *J. Am. Chem. Soc.* **128**, 15928-15928 (2006).
5. Bruce, V.J., Lopez-Islas, M. & McNaughton, B.R. Resurfaced cell-penetrating nanobodies: A potentially general scaffold for intracellularly targeted protein discovery. *Protein Sci.* **25**, 1129-1137 (2016).
