# Supplementary material for: Small molecule-nanobody conjugate induced proximity controls intracellular processes and modulates endogenous unligandable targets
Source: Nat Commun. 2023 Mar 24;14:1635. doi: 10.1038/s41467-023-37237-x (PMC10039045; doi:10.1038/s41467-023-37237-x)
Supplement: Supplementary file 2 — Description of Additional Supplementary Files [file 41467_2023_37237_MOESM2_ESM.pdf]

<u>Description of Additional Supplementary Files</u>

Title: Supplementary Data 1
Description: The Supplementary Data 1 file lists the primer sequences of the primers used fo r construction of plasmids in the study that involve PCR amplifications, or site-direct ed mutagenesis.

Title: Supplementary Software
Description: This Supplementary Software contains the "Manders_Coefficients.class" plugin for analysis of PCC value, and illustration of how to use it is also included.
