# Supplementary material for: Small molecule-nanobody conjugate induced proximity controls intracellular processes and modulates endogenous unligandable targets
Source: Nat Commun. 2023 Mar 24;14:1635. doi: 10.1038/s41467-023-37237-x (PMC10039045; doi:10.1038/s41467-023-37237-x)
Supplement: Supplementary file 5 — Reporting Summary [file 41467_2023_37237_MOESM5_ESM.pdf]

nature portfolio

Corresponding author(s): Xi Chen

Last updated by author(s): 2023-03-01

Reporting Summary

Nature Portfolio wishes to improve the reproducibility of the work that we publish. This form provides structure for consistency and transparency in reporting. For further information on Nature Portfolio policies, see our Editorial Policies and the Editorial Policy Checklist.

Statistics

For all statistical analyses, confirm that the following items are present in the figure legend, table legend, main text, or Methods section.

n/a	Confirmed	
☐	☒	The exact sample size (n) for each experimental group/condition, given as a discrete number and unit of measurement
☐	☒	A statement on whether measurements were taken from distinct samples or whether the same sample was measured repeatedly
☐	☒	The statistical test(s) used AND whether they are one- or two-sided<br>*Only common tests should be described solely by name; describe more complex techniques in the Methods section.*
☒	☐	A description of all covariates tested
☒	☐	A description of any assumptions or corrections, such as tests of normality and adjustment for multiple comparisons
☐	☒	A full description of the statistical parameters including central tendency (e.g. means) or other basic estimates (e.g. regression coefficient) AND variation (e.g. standard deviation) or associated estimates of uncertainty (e.g. confidence intervals)
☐	☒	For null hypothesis testing, the test statistic (e.g. F, t, r) with confidence intervals, effect sizes, degrees of freedom and P value noted<br>*Give P values as exact values whenever suitable.*
☒	☐	For Bayesian analysis, information on the choice of priors and Markov chain Monte Carlo settings
☒	☐	For hierarchical and complex designs, identification of the appropriate level for tests and full reporting of outcomes
☐	☒	Estimates of effect sizes (e.g. Cohen's d, Pearson's r), indicating how they were calculated

Our web collection on statistics for biologists contains articles on many of the points above.

Software and code

Policy information about availability of computer code

Data collection

Confocal Microscopy: ZEN Microscopy Software (Zeiss LSM880)
Fluorescence Spectra: SpectraMax® MiniMax™ 300 Imaging Cytometer (MD SpectraMax i3x Microplate Reader)
Isothermal Titration Calorimetry (ITC): MicroCal ITC200 Control Software v1.26 (MicroCal ITC200)
ÄKTA Pure: Unicorn 7.1 Build

Data analysis

Images: ImageJ/Fiji
Data Plotting: Microsolft Excel and Origin 2018
NMR: MestNova14.0 (Mestrelab Research)

For manuscripts utilizing custom algorithms or software that are central to the research but not yet described in published literature, software must be made available to editors and reviewers. We strongly encourage code deposition in a community repository (e.g. GitHub). See the Nature Portfolio guidelines for submitting code & software for further information.

Data

Policy information about availability of data

All manuscripts must include a data availability statement. This statement should provide the following information, where applicable:

- Accession codes, unique identifiers, or web links for publicly available datasets
- A description of any restrictions on data availability
- For clinical datasets or third party data, please ensure that the statement adheres to our policy

The data that support the finding of this study are available within the Article, Supplementary Information, or Source Data. Source data are provided with this paper.

Field-specific reporting

Please select the one below that is the best fit for your research. If you are not sure, read the appropriate sections before making your selection.

☒ Life sciences ☐ Behavioural & social sciences ☐ Ecological, evolutionary & environmental sciences

For a reference copy of the document with all sections, see nature.com/documents/nr-reporting-summary-flat.pdf

Life sciences study design

All studies must disclose on these points even when the disclosure is negative.

Sample size	No statistical method was used to pre-determine sample size; for all statistical analysis, representative results from no less than three groups of data were collected unless otherwise specified; for statistical Pearson's correlation coefficient analysis, typically 10 and more than 10 cells were analyzed.
Data exclusions	Typically, no data exclusions were performed, unless a single data biased too much from the average.
Replication	Unless otherwise specified, each experiment was repeated for at least times using independent cell preparations, and all are successful.
Randomization	No randomization was performed, due to the nature of these experiments.
Blinding	No blinding was applied, due to the nature of these experiments.

Reporting for specific materials, systems and methods

We require information from authors about some types of materials, experimental systems and methods used in many studies. Here, indicate whether each material, system or method listed is relevant to your study. If you are not sure if a list item applies to your research, read the appropriate section before selecting a response.

Materials & experimental systems

n/a	Involved in the study	
☐	☒	Antibodies
☐	☒	Eukaryotic cell lines
☒	☐	Palaeontology and archaeology
☐	☒	Animals and other organisms
☒	☐	Human research participants
☒	☐	Clinical data
☒	☐	Dual use research of concern

Methods

n/a	Involved in the study	
☒	☐	ChIP-seq
☒	☐	Flow cytometry
☒	☐	MRI-based neuroimaging

Antibodies

Antibodies used	GFP tag rabbit mAb: ZENBIO, #R24437<br>Goat anti rabbit IgG H&L (HRP): ZENBIO, #511203<br>TPX2 rabbit mAb: ZENBIO, #R27376<br>Alexa Fluor 488 labeled secondary antibody (Cat# 550037, ZENBIO)
Validation	The antibody was validated before use.

Eukaryotic cell lines

Policy information about cell lines

Cell line source(s)	HeLa cell (Cat#CL-0101) and HepG2 cell (Cat#CL-0103) are provided by Procell Life Science & Technology Co., Ltd.
Authentication	No additional authentication was performed; Procell Life Science & Technology authenticates the cell lines using STR analysis according to the production information.
Mycoplasma contamination	All cell lines were confirmed to be mycoplasma free before use.
Commonly misidentified lines (See ICLAC register)	No commonly misidentified lines were used.

Animals and other organisms

Policy information about studies involving animals; ARRIVE guidelines recommended for reporting animal research

Laboratory animals	The immunodeficient BALB/c nude female mice of over 4-6 weeks old. Sex was not considered in the study design because hepatocarcinoma is not a gender-based disease.
Wild animals	None
Field-collected samples	None
Ethics oversight	Regarding mice, animals were maintained under specific pathogen-free (SPF) conditions and handled based on the approval by the Institutional Animal Care and Use Committee of Harbin Institute of Technology (IACUC/HIT) with the permit number IACUC-2021052.

Note that full information on the approval of the study protocol must also be provided in the manuscript.
